# Automated Quantification Reveals Hyperglycemia Inhibits Endothelial Angiogenic Function

**DOI:** 10.1371/journal.pone.0094599

**Published:** 2014-04-09

**Authors:** Anthony R. Prisco, John D. Bukowy, Brian R. Hoffmann, Jamie R. Karcher, Eric C. Exner, Andrew S. Greene

**Affiliations:** 1 Biotechnology and Bioengineering Center, Medical College of Wisconsin, Milwaukee, Wisconsin, United States of America; 2 Department of Physiology, Medical College of Wisconsin, Milwaukee, Wisconsin, United States of America; Medical University Innsbruck, Austria

## Abstract

**Objective:**

Diabetes Mellitus (DM) has reached epidemic levels globally. A contributing factor to the development of DM is high blood glucose (hyperglycemia). One complication associated with DM is a decreased angiogenesis. The Matrigel tube formation assay (TFA) is the most widely utilized *in vitro* assay designed to assess angiogeneic factors and conditions. In spite of the widespread use of Matrigel TFAs, quantification is labor-intensive and subjective, often limiting experiential design and interpretation of results. This study describes the development and validation of an open source software tool for high throughput, morphometric analysis of TFA images and the validation of an *in vitro* hyperglycemic model of DM.

**Approach and Results:**

Endothelial cells mimic angiogenesis when placed onto a Matrigel coated surface by forming tube-like structures. The goal of this study was to develop an open-source software algorithm requiring minimal user input (Pipeline v1.3) to automatically quantify tubular metrics from TFA images. Using Pipeline, the ability of endothelial cells to form tubes was assessed after culture in normal or high glucose for 1 or 2 weeks. A significant decrease in the total tube length and number of branch points was found when comparing groups treated with high glucose for 2 weeks versus normal glucose or 1 week of high glucose.

**Conclusions:**

Using Pipeline, it was determined that hyperglycemia inhibits formation of endothelial tubes *in vitro*. Analysis using Pipeline was more accurate and significantly faster than manual analysis. The Pipeline algorithm was shown to have additional applications, such as detection of retinal vasculature.

## Introduction

The first demonstration of the ability of vascular endothelial cells to rapidly form tube-like structures on a gel composed of basement membrane proteins was published in 1988. [1] Since that time this phenomenon has been shown to be specific to endothelial cells as other types of cells form different structures when placed on a basement membrane substrate. [2]–[4] The most commonly used substrate for assessment of endothelial cell tube formation is growth factor reduced Matrigel. Matrigel is a gelatinous substance secreted from Engelbreth-Holm-Swarm (mouse sarcoma) cells containing many proteins found in the extracellular environment. [2] Due to similarities with *in vivo* angiogenesis, TFAs have been used as a model for studying the growth of new vessels *in vitro*.

**Figure 1:**
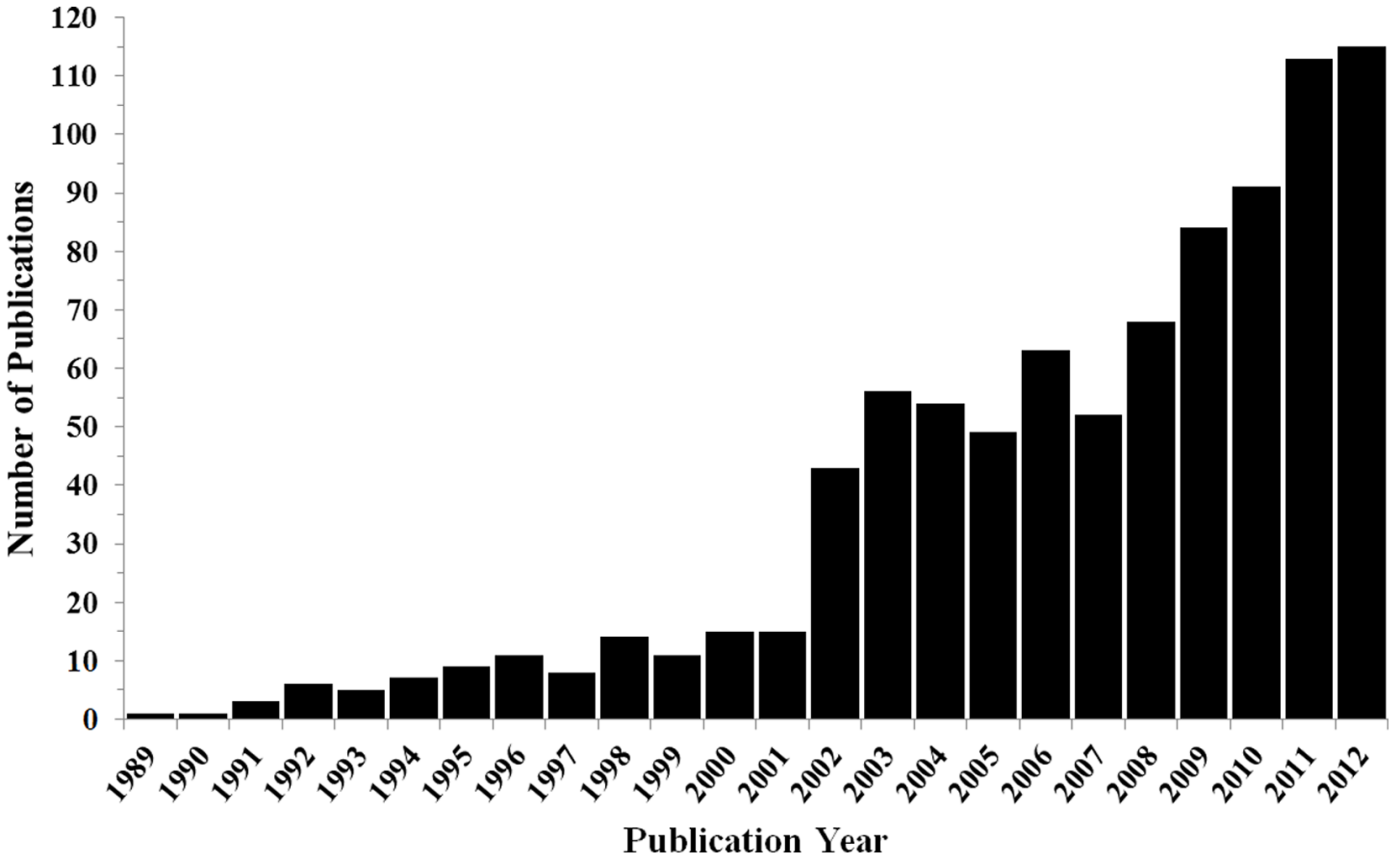
Tube formation publication trends are indicated. Pubmed was searched between 1989 and 2013 for “Journal Articles” containing the key words ‘matrigel’ and ‘tube formation’ returning a total of 949 matches. After further filtering, 894 articles were returned between 1989 and 2012; 319 (36%) since 2010. These results demonstrate the increasing use of the Matrigel TFA as a viable laboratory assessment of EC function within the literature. A complete list of publications is shown in **Table S1**.

Nearly 1000 publications using *in vitro* TFAs demonstrate the importance of this technique in the evaluation of endothelial cell function (**Figure 1**). Occasionally, differences observed between control and experimental conditions within a study are obvious, omitting the need for quantification. However, most studies have investigated non-obvious differences between groups that required a systematic quantification of features observed within the TFA. Different features of TFAs have been quantified depending on the study, the most common being the total tube length, tube area, and number of branch points in the tube network. Quantifying features in TFA images is labor intensive and subject to both inter- and intra- user variability. TFA analysis falls into four categories (citations refer to example studies, see **Table S1** for a complete list). 1) No quantification,[5]–[9] 2) Manual thresh holding for total tube area or intensity,[10]–[12] 3) Manual analysis of total tube length, area, or number of branch points,[13]–[18] 4) Automatic quantification of total tube length using fluorescent labels. [19]–[22] Current methods for automation quantification require significant constraints to both the experimental method and the range of analyses that can be performed, most notably, the addition of cytotoxic dyes.

There are several commercially available software tools that automatically analyze TFAs. These programs are powerful because they reduce the time required to complete an analysis and the results are easily reproducible. These algorithms all share the general feature that they identify patterns within an image that represents tubes and generate a quantitative report describing the quantified metrics of the tubes within an image. To be useful, an algorithm must not only have a sensitivity that maximally recognizes tubes, but it must also have a selectivity that excludes debris and tubes that are poorly formed. The most difficult obstacle to overcome in the development of a useful automated method is achieving sufficient tube selectivity while also maintaining sensitivity. Several methods attempt to achieve this goal by labeling cells prior to the TFA and then imaging the specifically labeled marker with the goal of excluding non-cellular material within an image. This technique provides a high contrast image that can be specific for a target cell of interest, however it requires a cellular stain [23], [24] which can be problematic as many stains exhibit cellular toxicity that interfere with cell function. [25] In addition, current software tools that automatically analyze TFAs are typically sold as add-on packages to existing image analysis software, which could be cost-prohibitive for many groups.

In this study, the development and validation of an open source software tool that can automatically import and analyze non-stained TFA images is described. The software, known as “Pipeline,” returns a report of total tube length, area, and nodal branch points for each TFA image and can operate either in a “sandbox” or fully automated mode. In the single analysis “sandbox” mode, a user can tweak analysis parameters on a single representative image. The parameter profile can then be saved and imported into the fully automated mode for analyzing a large group of images in a high throughput fashion. In the fully automated mode, the parameter profile is the only requirement needed to analyze an entire set of images generated in a single study. No additional user input is required. Additionally, the software is capable of generating output images showing how the features of each image were interpreted and quantified. This feature allows for retrospective evaluation of each image analyzed in the automated high throughput mode and helps a user evaluate the quality of analysis and further optimize the algorithm’s parameters to meet the needs of the study.

Type II diabetes, otherwise known as Diabetes Mellitus (DM), is a condition that occurs when a person becomes insensitive towards insulin causing an increase in bloodstream glucose levels (hyperglycemia). [26] DM has reached epidemic levels globally with an incidence of 250 million that is expected to double by 2030. [27] Hyperglycemia in DM causes the buildup of advanced glycosylated end products inducing disease related complications. Complications include heart disease, stroke and kidney failure. Blood vessel formation (angiogenesis) is also impaired in DM. In DM patients who also have Peripheral Artery Disease (PAD), impaired angiogenesis can limit the ability to recover from ischemic events ultimately leading to loss of extremities. [28]–[31] The exact mechanisms by which angiogenesis is impaired in DM are unknown. While excessive fructose intake has been shown to increase the risk of developing DM, it has not been shown if high blood glucose levels or the mechanisms of insulin insensitivity are responsible for decreased angiogenesis observed in DM patients. [32] In order to develop better treatments, it is important to elucidate the mechanisms by which angiogenesis is impaired in DM. There are many *in vitro* and *in vivo* models of angiogenesis utilized throughout the literature, including the chick chorioallantoic membrane assay, the retinal angiogenesis assay and the Matrigel tube formation assay (TFA). The focus of this study is primarily on a software method developed to automatically quantify Matrigel TFAs. This software was used to develop and validate an *in vitro* model of hyperglycemia. To show this, rat cardiac microvascular endothelial cells (RCMVECs) were cultured in normal or high glucose for 1 or 2 weeks and their ability to form tubes was measured. Pipeline was used to analyze TFA images of RCMVECs in a high throughput fashion. The images were also analyzed manually by two independent operators and the results compared to those returned by the Pipeline tool. All three analyses demonstrated significant differences in total tube length and nodal branch points during the TFA. Using this approach, it was demonstrated *in vitro* that hyperglycemia could be a causative mechanism of impaired angiogenesis.

## Materials and Methods

### Analysis of Matrigel Tube Formation Publication Trends

Original tube formation analyses are most oft attributed to Kubota et al. *J of Cell Bio.* 1988. In PubMed, journal articles were searched for ‘matrigel’ and ‘tube formation’ in combination from 1989 to 2012 (search May 6, 2013) where total publications per year were plotted (**Figure 1**). Supplemental data contains all article information resulting from the search parameters (**Table S1**).

### Cell Culture

RCMVECs (R1111; Cell Biologics; Chicago, IL) were cultured (P2–P4) on gelatin-coated 100 mm plates in EC specific media (MCDB131, E3000–01B; US Biological; Swampscott, MA) supplemented with 10% fetal bovine serum and the microvessel EGM-MV supplement pack (CC-4147; Lonza; Basel, Switzerland). Media was supplemented with normal glucose (5.6 mM) or switched to high glucose (25 mM) and cultured for one and two week intervals to determine the effects of simulated-hyperglycemia on tube formation.

### Tube Formation Assay

RCMVECs were washed three times with Dulbecco’s phosphate-buffered saline (DPBS, Invitrogen), lifted using Enzymatic Free Cell Dissociation Buffer (Millipore) for 15 minutes at 37°C, manually disrupted if necessary, collected, centrifuged at 100×g for 5 minutes, and washed twice with DPBS. RCMVECs were resuspended in 1 mL of the appropriate media per treatment group, counted on a Cell Countess, and diluted accordingly. 20,000 RMVECs were added accordingly to each chamber in 1 mL of media on a four-chamber slide (Nunc Lab-Tek). Chambers were coated with 250 μL of Growth Factor Reduced Matrigel (BD Biosciences) under chilled conditions followed by solidification at room temperature prior to RCMVEC addition. After 24 and 48 hours incubation at 37°C on the Matrigel, 4× and 10× magnification images were taken of the RCMVEC tube formation on a Nikon TS-100 Microscope with Flex camera (Nikon).

### Tube Formation Assay Analysis

TFA images collected on an inverted phase contrast microscope (Nikon, Tokyo, Japan) were analyzed in Metamorph (Molecular Devices, Sunnyvale, CA) by manually tracing connected tubes. Four 4× magnification images of tube formation per group were quantified at 24 and 48 hour time points. Tracings were analyzed for total tube length per field. A two way analysis of variance was used to compare differences between groups. Significance was set at p<0.05. Statistics were performed using SigmaPlot (Systat software, Chicago, IL) statistical software, version 12.0.

### Software Development

Software was developed in MATLAB R2012b using the Imaging Toolbox v27. Code was compiled in ‘C’ and will run on any computer with the MATLAB R2012b×64 runtime environment (http://www.mathworks.com/products/compiler/mcr/index.html). A screencast introducing Pipeline and explaining its capabilities is available online (http://www.youtube.com/watch?v=IwxtbGT1vDI) as well as a screencast demonstrating how to set up and run Pipeline on any computer (http://www.youtube.com/watch?v=FGIZzOASFNw).

## Algorithm Development

**Figure 2:**
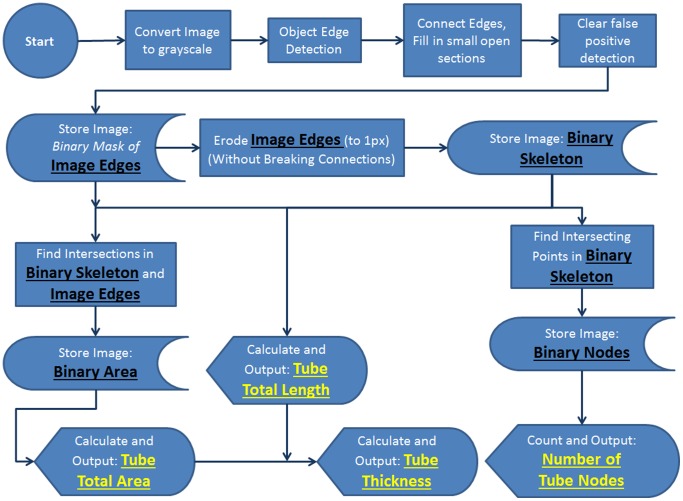
The overall analysis algorithm flowchart for Pipeline is displayed above. TIFF files are imported, converted to a grayscale image, edges are detected using the “Canny” method, and a binary mask called ‘tube edges’ is created. The mask is then skeletonized forming an additional binary mask called the ‘skeleton image’. The total tubular area, length, thickness, and number of branchpoints is then calculated from these two images.

Imaging algorithm was developed in MATLAB R2012b using the Imaging Toolbox v27. The algorithm summary flowchart can be found in **Figure 2**. The source code and compiled version can be found at sourceforge.net (http://sourceforge.net/projects/pipelinetfaanalysis/).

### Loading/Converting Original Image

The software imports a bright field TIFF image and converts it into (3) 2D matrices (Red, Green, Blue). In the Pipeline single analysis mode, only one image is loaded and processed, while the batch analysis mode a list of file names/locations is loaded and each file is processed one at a time. The (3) 2D matrices are averaged and at each location to form (1) matrix representing a black and white image. The luminosity of the grayscale image is then scaled to that of the original image.

### Tube Detection

The loaded black and white image is processed with an edge detection method known as the ‘Canny Method.’ The output of this step is a binary mask representing the locations of detected tube-like structures. This output is further processed by connecting pixels determined to be edges within a user-defined number of pixels (the parameter referred to as ‘Canny’ in **Table 1**). This mask is dilated and contracted to connect nearby objects. To further refine the mask, small areas of pixels (user-defined parameter ‘Fill’ in **Table 1**) determined not to be edges but surrounded by edge pixels (false negatives) are filled in and small areas of pixels determined to be edges (false positives), but surrounded by non-edges are cleared (user-defined parameter ‘Clean” in **Table 1**). These steps create a binary mask representing all areas of the image where a tube-like structure has been detected.

**Table 1:** User Adjustable Parameters in Pipeline.

Parameter	Function	TypicalRange	ImageGenerated	Units
Scale	Pixel scaling (ex. 1 pixel = 1.8 μm)	0.5–5	None, scales outputs	Microns
Canny	After applying the Canny method, creates mask by connecting pointsthat are within the specified number of pixels	2–12	Detected Edges	Pixels
Dilate	After connected points from the Canny method, the entire mask isexpanded by this many pixels to reduce noise	2–7	Selected Objects	Pixels
Fill	After creating a binary mask using the Canny method, non-maskedareas smaller than this parameter completely surrounded by maskedarea are filled in	200–5000+	Selected Objects	Pixels
Clean	After creating a binary mask using the Canny method, masked areassmaller than this are cleared	1000–10000+	Selected Objects	Pixels
Clip	Trims non-connected tubes down by the specified number of pixelsor two a branch intersection, whichever is first	50– Inf*	Tube Overlay	Pixels
Overlap	When calculating area, the each object within binary mask createdfrom the canny method must overlap the tube mask by this manypixels to be counted	10	Area Overlay	Pixels
Radius	To clean up redundant branchpoints, all detected branchpointswithin this radius will be averaged into a single branch point	30–150	Node Overlay	Pixels

The parameters listed within the table are user adjustible within the software to optimize an analysis depending upon the quality and magnification of the input image.

**Note:** The user adjustable parameters available in Pipeline, along with associated descriptions and typical ranges for utilization by the user are indicated.

*Inf refers to “Infinity” where the code performs iterations until there is no change between subsequent iterations.

From this binary mask, the detected tube-like structures are eroded to a structure with the width of a single pixel to generate a binary mask outlining the detected image backbone. The binary mask representing tube structures is scanned for distinct objects (groups of adjacent pixels). Each object is iteratively eroded by removing object edge pixels provided the removal of a pixel does not break a single object into multiple objects (the Euler characteristic, an index describing the shape of an object, is preserved). [33] The algorithm continues to erode the image objects until the mask is not changed in successive iterations.

To clean up artifacts introduced when generating the tube backbone, end points of the skeleton image are removed by either a user set number of pixels (‘Clip’ parameter, **Table 1**) or until a branch point is found (a single pixel connected to 3 or more pixels). This stage of analysis is necessary to exclude quantification of small, tube-like structures.

### Calculation of Metrics/Output Files

The total length of tubes generated in the assay is determined from the mask of the tube backbone. This metric is calculated by detecting the connectivity relationship of each connected pixel. Pixels connected horizontally/vertically are added as the length of a single pixel. Pixels connected diagonally are added as the length of a single pixel multiplied by 1.414. The tube backbone image is dilated by 10 pixels, overlaid onto the original loaded bright field image, and saved as a TIFF. This image represents how the total tube length was quantified.

The mask of detected image objects and the mask of the detected image backbone are overlaid and any object where there is not a minimum user-defined overlap number of pixels (‘Area’ parameter, **Table 1**) is cleared in the detected areas mask. Simply put, to be included in the area calculation, objects initially detected as tube-like structures must contribute to the total tube length calculation. This mask is inverted, overlaid onto the original loaded bright field image, and saved as a TIFF. This image represents how the total tube length was quantified.

To determine the number of tube branch points, the tube backbone mask is analyzed for connectivity. Any pixel connected to 3 or more pixels is considered a branch point. Pixels considered to be branch points are saved onto a separate binary mask. This mask is analyzed for redundant branch points in close proximity. A user defined search parameter (‘Radius,’ **Table 1**) sets a maximum radius to search for redundant branch points. The algorithm will search each detected branch point for additional branch points. All points detected within that radius will be spatially averaged and represented as a single branch point. The identified branch points are dilated by 10 pixels and overlaid onto the original bright field image. This image represents how the branch points in the original image were quantified.

The tube area, length, number of nodes, and average thickness is either displayed (single analysis mode) or saved to an excel spreadsheet along with the file name (batch analysis mode). For datasets processed using the batch analysis mode, the user has the option to save images generated during the analysis for retrospective validation of results.

#### Validation

**Figure 3:**
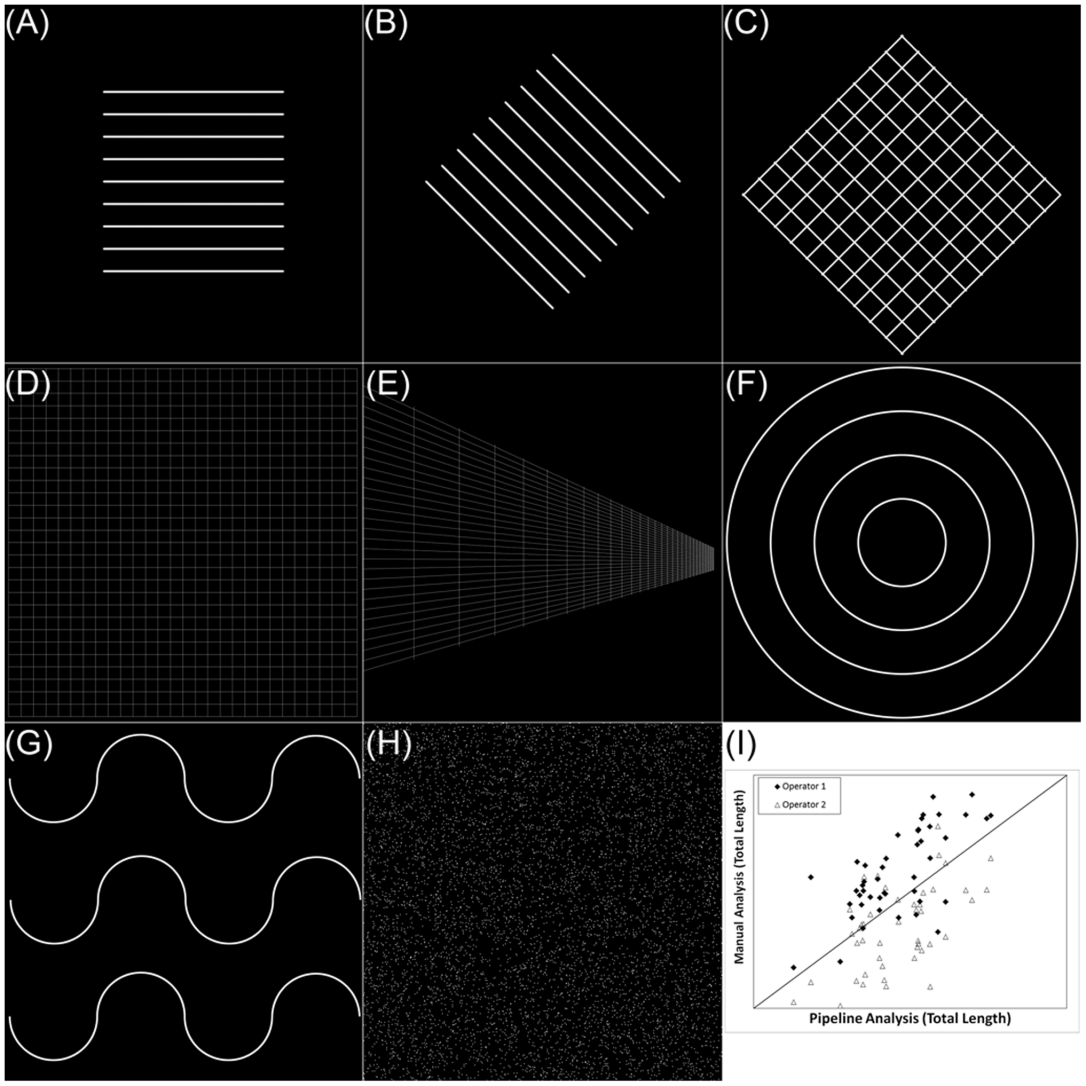
The Pipeline algorithm was validated using images generated *in silico*. This figure shows representative images used to develop and validate the portions of the code that quantify tube length and branch points. For example, (A) was the first test image used to develop the length measurement algorithm. That image was then rotated 45° (B) to ensure the algorithm returned the same length measurement. Additional complexity was added (C, D, E, F and G) to validate the length algorithm. The number of branch points was developed initially in (C) and was further tested for robustness in (D and E). To develop an algorithm that searched for and consolidated duplicate branch points located in close proximity, salt and pepper noise was analyzed (H). To validate the tool, two separate operators manually traced tubes in a single data set (I). The manual analysis was compared with the automatic analysis returned by pipeline.

The Pipeline algorithm was initially developed analyzing images generated *in silico* of known lengths, areas and branch points (**Figure 3**). The algorithm was adjusted until each feature within the image was identified within 99% of the actual value. A test set of 20 images was used to evaluate the algorithm. The algorithm was additionally adjusted and a new test set of 50 images was analyzed and compared to a manual analysis of total tube length (two users). All images used in the 2^nd^ test set in the Pipeline analysis were images of tubes grown in both high glucose conditions (1 week and 2 weeks). Analyses by both users and Pipeline all indicated a statistically significant decrease in total tube length from tubes grown in high glucose for two weeks versus one week indicating an accurate result.

## Results

### Tube Formation Assay Publication Trends

Upon review of the literature in PubMed, trends were observed for Matrigel tube formation assays in scientific journal articles. PubMed was searched on May 6, 2013 and a total of 949 publications between 1989 and 2013 containing ‘matrigel’ and ‘tube formation’ in the text were found. Publication numbers, 894 manuscripts between 1989 and 2012, indicate a growing trend in the use of tube formation as a viable model for analysis of EC function (**Figure 1**). The four most common observable methods of tube quantification in these assays were 1) qualitative conclusions drawn, no quantification, 2) manually thresh holding for total area occupied by tubes, 3) manual quantification for tube length or branch points, and 4) automatic quantification for tube length using fluorescence.

### Pipeline GUI and Used Adjustable Parameters

**Figure 4:**
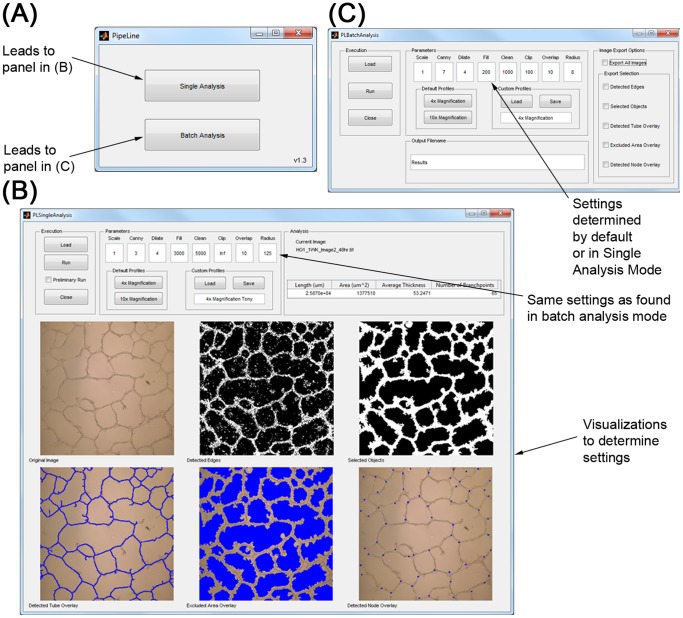
Pipeline graphical user interface components are as follows: (A) Opening Panel, allows user to select imaging processing mode. (B) Single Processing mode, allows user to manipulate and optimize (7) analysis parameters for a single image. Analysis parameters can then be used in Batch Analysis mode to process an entire group of images. (C) Batch Analysis processing mode, allows a user to select multiple images for analysis with a set of parameters optimized within the single processing mode. In Batch Analysis mode, the 6 images generated in the single processing mode can be exported as TIFFs to separate folders.

Pipeline was written in MATLAB using the image processing toolbox, complied in ‘C’ and will run on any computer with the freely available MATLAB R2012b×64 runtime environment (http://www.mathworks.com/supportfiles/MCR_Runtime/R2012b/MCR_R2012b_win64_installer.exe). Pipeline has two operational modes, a single analysis “sandbox” mode and a batch analysis mode (**Figure 4**). The software was designed for a user to first import a representative image into the single analysis mode to determine the optimal set of parameters to analyze an entire dataset (**Table 1**). After the optimal set of analysis parameters is determined the user can save the parameters, transfer those settings into the batch analysis mode, and import a large number of images for analysis under the same conditions. To determine if the analysis was performed correctly, a user can choose to “save” the analyzed images in separate directories to retrospectively ensure an accurate analysis was performed.

### Pipeline Analysis Algorithm

**Figure 5:**
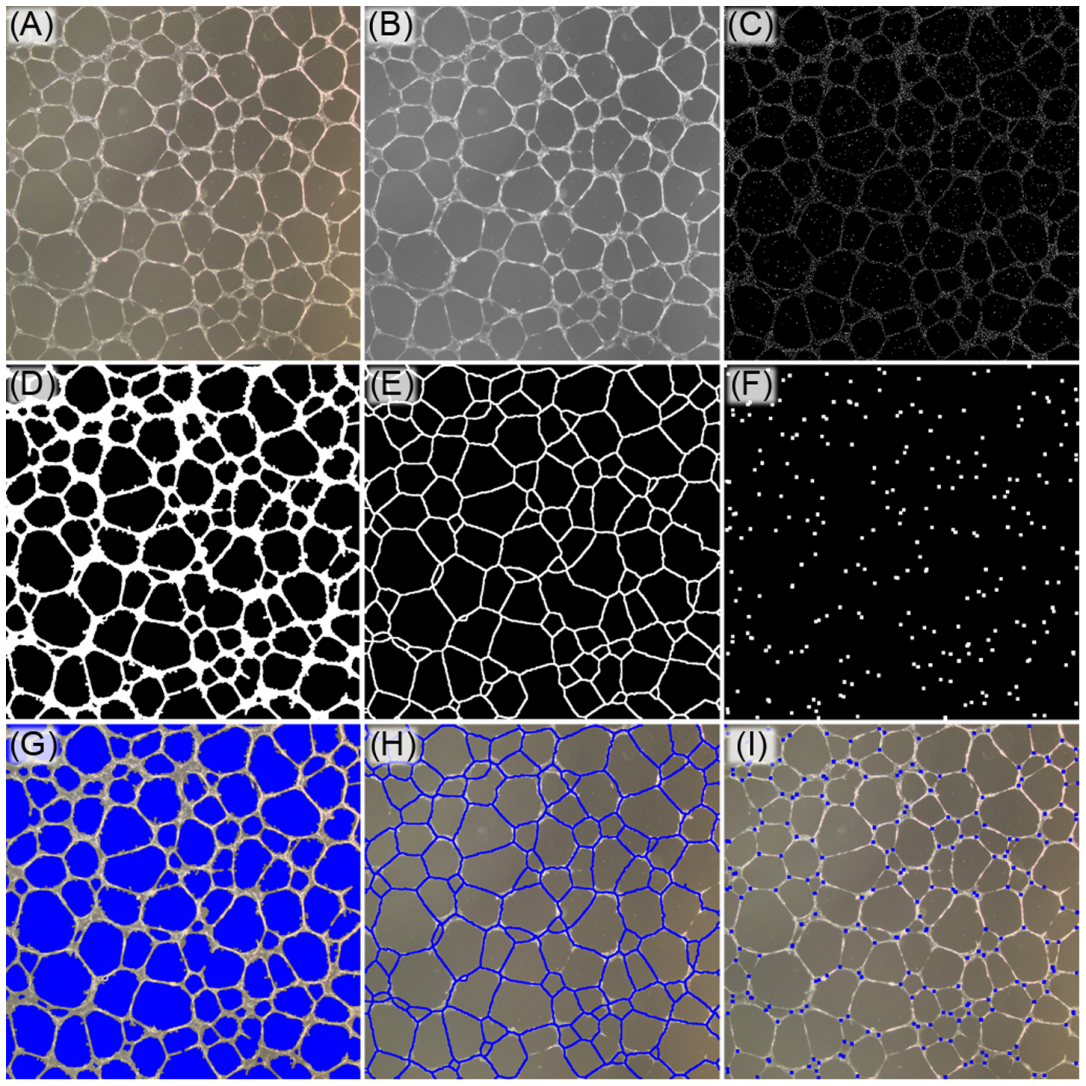
Indicated is a visual demonstration of the step-by-step analysis of the Pipeline algorithm. (A) The image is imported and (B) converted to grayscale. (C) Tube edges are detected using the Canny Method, (D) connected creating a binary mask, and (E) the binary mask is iteratively eroded without breaking connectivity to find total tube length. (F) Intersections within the skeleton are then detected for nodal branch points. (G–I) Original images are then displayed with the calculated area, length, and a branch point mask overlay to display the algorithm quantified image features.

To analyze a non-fluorescent bright-field image (**Figure 5** **, A**), an image is first converted to grayscale (**Figure 5** **, B**). The initial step in the analysis is to generate a binary mask indicating the coordinates of specific locations within an image containing tubes. To do this, the image is analyzed using the Canny Method that specifically searches an image for object edges by finding pixels with a high spatial derivative. [34] The Canny Method generates a binary mask at an identical resolution to the input image (**Figure 5** **, C**) where pixels with a value of ‘1’ indicate a detected ‘edge-point.’

Pipeline connects these points if they are within a user-defined number of pixels. Because the Canny method detects object edges, there is a user defined parameter that identifies small areas of pixels assigned to a value of ‘0’ that are located between tube edges. The algorithm fills these points in and deletes any small areas of pixels that may have been falsely identified as ‘edges.’ To filter out any remaining noise, additional pixels are connected by spatially dilating the positive pixels within the binary mask (**Figure 5** **, D**). From this binary mask, the Pipeline algorithm calculates total tube length, area and number of branch-points (**Figure 2** **,** **1**, - Image Edges mask).

Tube length is calculated by iteratively eroding the edges of the Binary Mask of Image Edges by a single pixel. The erosion is applied at each pixel location within the binary mask where single erosion iteration would not cause a single group of pixels to be separated into distinct group (preservation of the Euler characteristic). The erosion is carried out until there are no pixels in the image that can be successfully eroded without separating pixel groups (**Figure 5** **, E**). When eroded down to the width of a single pixel, a new binary mask is saved that represents the total tube length (**Figure 2**, - Binary Skeleton mask). From this mask, total tube length is quantified from the number of pixels contained in the Binary Skeleton and the connectivity relationship of each pixel. Pixels that are connected horizontally/vertically are weighted in the length calculation as the length of a single pixel. Pixels connected diagonally are weighted in the length calculation according to the equivalent of a single pixel multiplied by the Pythagorean relationship of right isosceles triangle (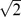). Tube branch points are determined from the Binary Skeleton by identifying pixels that are connected to 3 or more pixels (**Figure 2**, - Binary Nodes). This algorithm often identifies multiple nodes at a single branch point site, so a user can specify to reduce multiple nodes identified within close proximity to be reduced to a single pixel (**Figure 5** **, F**).

To calculate total tube area, the Image Edges mask is intersected with the Binary Skeleton mask. The steps between Image Edges and Binary Skeleton involve several steps of noise reduction and cleanup. More simply, for an object’s area to be included in the calculation, the cleanup between Image Edges and Binary Skeleton cannot completely reduce the object from the image (i.e. an object must contribute to the Binary Skeleton mask for its area to be considered). Average thickness is calculated by dividing the total tube area by total tube length. To complete the analysis, the masks Binary Nodes, Binary Skeleton and Image Edges are overlaid on the original input image and saved as a record of analysis (**Figure 5** **, G–I**). The four outputs (total tube length, total tube area, average tube thickness, and number of branch points) are saved in an excel spreadsheet (Summary in **Figure 2**).

### Pipeline Analysis Comparison to Manual Quantification

**Figure 6:**
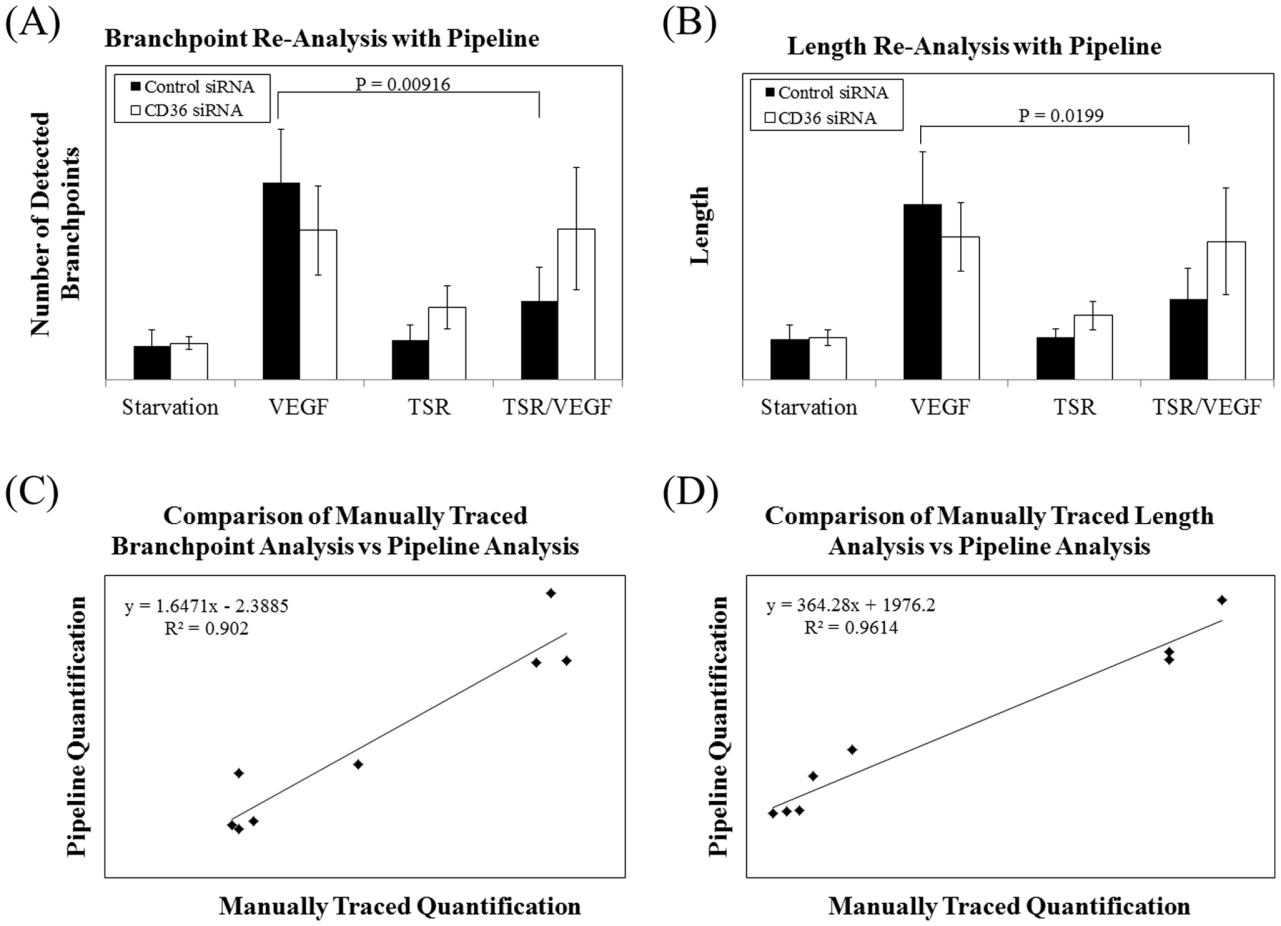
Comparison of Manual Quantification in Liu et Liu analyzed the number of branchpoints and relative tube length by manually tracing structures in ImageJ. After analyzing the same raw data in Pipeline, the same trends were observed when analyzing (A) branchpoints and (B) tube length. In both panels, statistical significance was maintained. When comparing manual tracing to Pipeline quantification, both metrics were found to be highly correlated. (C) Branchpoint analysis correlation: R^2^ = 0.902 and (D) length analysis correlation: R^2^ = 0.961.

To compare quantification using Pipeline to manual quantification by tracing, raw images were obtained from a study conducted by Chu et al [35] utilizing TFAs as a key study metric. To repeat the analysis conducted in Figure 5 by Chu et al, it took approximately 1 minute of user input to set up a complete analysis in Pipeline and 5 minutes to verify an accurate result whereas it took 180 minutes to manually quantify images by tracing (**Table 2**). Pipeline was able to reproduce the analysis trends for both branchpoints (**Figure 6** **, A**) and tube length (**Figure 6** **, B**) while preserving statistical significance between groups. Concordance of quantification obtained using Pipeline and by manual tracing and was found to be high with an R^2^ = 0.902 for branchpoint analysis (**Figure 6** **, C**) and R^2^ = 0.961 for length analysis (**Figure 6** **, D**).

**Table 2:** Comparison of Operator Required to Complete Total Length Analysis.

	Manual Analysis by Tracing (minutes)			Pipeline Analysis (minutes)	
	Operator 1, Glucose Study	Operator 2, Glucose Study	Analysis from Chu et al	Glucose Study	Analysis from Chu et al
Set Up	0	0	0	7	1
Analysis	240	240	180	Automated	Automated
Verification	0	0	0	10	5
**Total**	**240**	**240**	**180**	**17**	**6**

**Figure 7:**
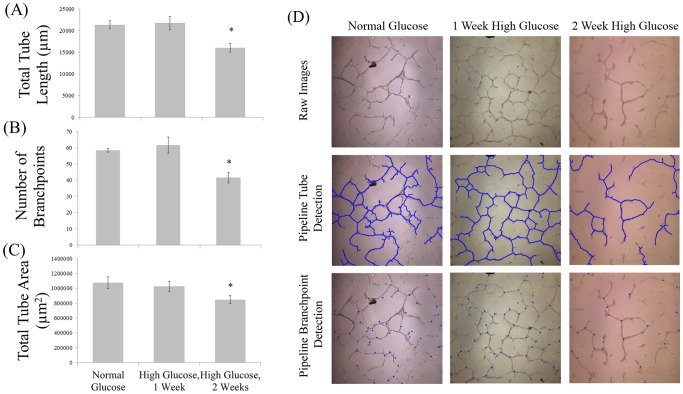
Pipeline analysis was demonstrated through evaluating the effects of simulated-hyperglycemia on RMVEC tube formation *in vitro*. RMVECs were cultured in normal glucose (5.6 mM) or high glucose (25 mM) for 1–2 weeks. Cells were then isolated and 20,000 RMVECs were incubated at 37°C for 24 and 48 hours on Matrigel in a four-well chamber. Two week exposure to high glucose significantly decreased both (A) total tube length and (B) the number of nodal branch points detected and (C) total tube area (p<0.05). (D) Representative input/output images from each condition.

Time required to complete the analysis by manually tracing or by Pipeline (all times in minutes). In the present study (data displayed in **Figure 7**) it took each operator approximately 240 minutes to complete the analysis by manually tracing tube-like structures. Completing the same analysis in Pipeline took approximately 7 minutes to set up the analysis and 10 minutes to examine the output images to verify an accurate result. When re-analyzing the data from Chu et al, it took only 1 minute to set up the analysis in Pipeline and 5 minutes to verify an accurate result as opposed to 180 minutes to complete the analysis through manual tracing.

### In vitro Model of RCMVEC Tube Formation during Simulated-hyperglycemia

RCMVECs were cultured in normal glucose (5.6 mM) or switched to high glucose (25 mM) media for 1 or 2 weeks. Cells were seeded onto cell culture chambers coated with growth factor reduced Matrigel and allowed to grow tubular networks for 24 or 48 hours at 37°C. During image analysis of TFAs with Pipeline, significantly less tube formation was detected (p<0.05) under conditions of high glucose for two weeks versus one week of high glucose or under normal glucose conditions (**Figure 7**). The differences observed using Pipeline to analyze for these conditions were a reduced total tube length, (**Figure 7A**) number of nodal branch points (**Figure 7B**) and overall area (**Figure 7C**).

### Inter-Operator Variability

Features observed in images of TFAs need to be quantified, especially when images from the assay are demonstrating subtle, but significant differences between treatments. In the hyperglycemia model analyzed in this study, there was an observed total tube length reduction of 25% when comparing cells grown in high glucose for 2 weeks versus cells grown in normal glucose (**Figure 7**). While this finding was statistically significant, this difference was suspected, [36] but not obvious prior to conducting a total length analysis. Two independent operators analyzed the 1 week high glucose vs 2 week high glucose data sets obtaining similar results (**Figure 3** **, I**). Interestingly in this validation set, mean inter-operator discrepancy when analyzing images with well-formed tubes was 38% (**Table 3**, Images with a total tube length greater than 15,000 μm). The inter-operator discrepancy was determined by calculating the relative percent difference (**Equation 1.1**):
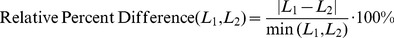
(1.1)Where ‘L_1_’ represents the total quantified length by operator 1 and ‘L_2_’ represents the total quantified length by operator 2. When comparing all images within this validation set, the maximum difference between operators was more than 17-fold. The majority of the discrepancy was attributed to whether each operator quantified poorly formed tube networks as debris or tubes. **Table 3** is suggestive of this because the variability within the validation set increases when images with poorly formed tubes are included in the analysis as poorly formed features were quantified on some images but not quantified on others.

**Table 3:** Inter-Operator Analysis Variability Expressed as Absolute Percent Difference.

Comparison of Manual Analysis Between Independent Operators			
Difference BetweenOperators	All Images	Images Greater than 10,000 μmin total length	Images Greater than 15,000 μmin total length
Minimum	2.47%	2.47%	2.47%
Maximum	1754.03%	234.12%	139.23%
Average	170.70%	66.57%	38.30%
Median	71.70%	59.84%	26.61%

When comparing total tube length results as analyzed between operators 1 and 2, a large amount of variability was observed between the two analyses. The relative percent difference between operators was calculated by dividing the absolute difference in quantitation of a single image with the value obtained by the operator that quantified the image a lower total tube length (**Equation 1.1**). The largest relative percent differences in quantification between operators occurred in images with poorly formed tubes which explains the increased concordance between operators as images with a lower total quantified length were removed (images where either operator quantified the total tube length within an image less than 10,000 μm or 15,000 μm).

An automated tool containing capabilities to save analysis parameters to a profile eliminates intra- and inter-user variability increasing reproducibility while decreasing user effort. When comparing the amount of operator time required to complete the analysis in **Figure 3** **, I** it took each operator approximately 240 minutes to manually analyze all 50 images (**Table 2**). When completing the analysis with Pipeline, it took the operator only 7 minutes to set up the analysis. The computer completed the analysis in 150 minutes and then an additional 10 minutes of operator time was required to review the output images ensuring an accurate analysis was conducted (**Table 2**).

### Analysis of the Retinal Vasculature

**Figure 8:**
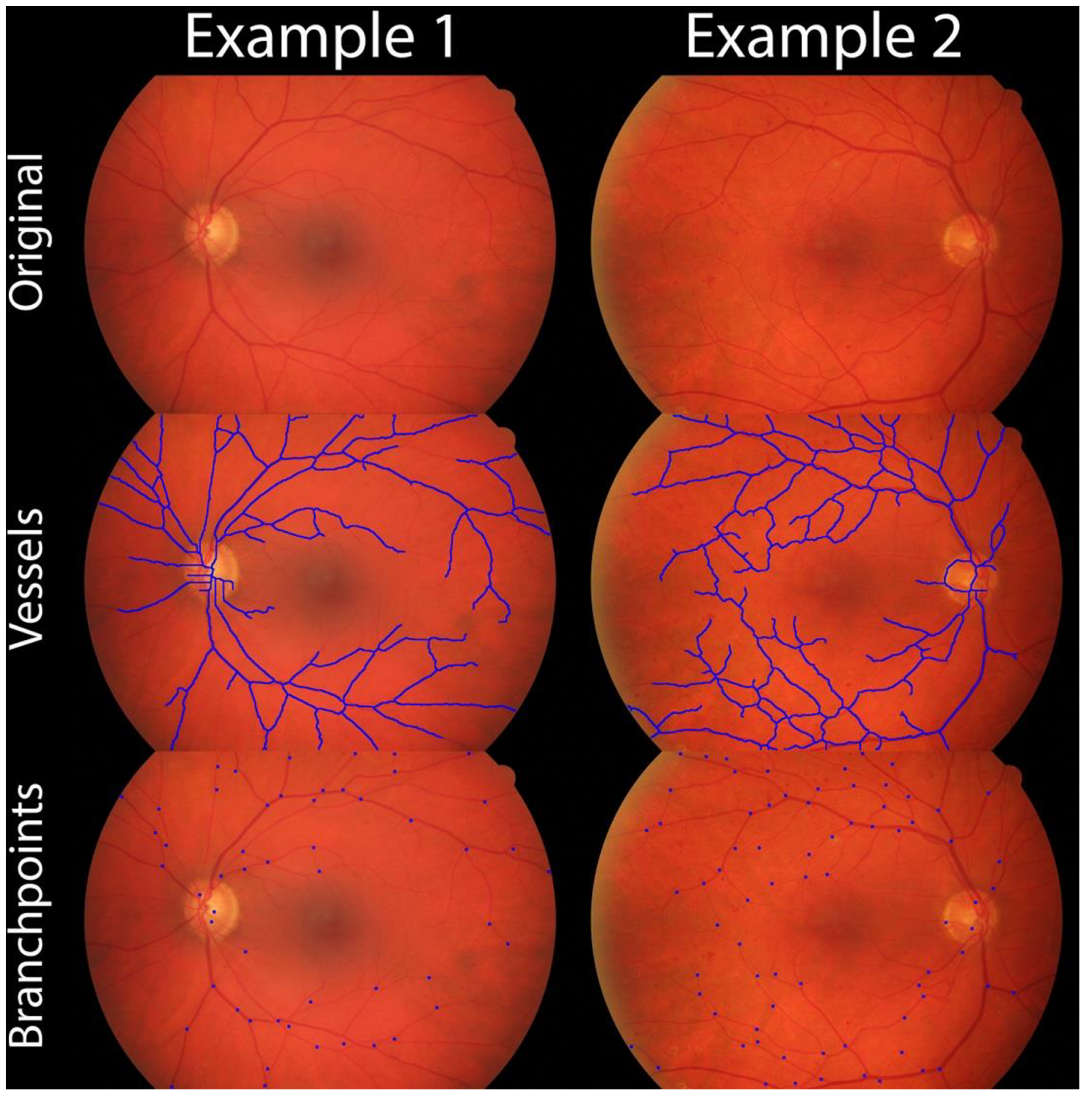
The Pipeline algorithm was used to identify vessels and vessel branch points contained within retinal fundus images. The algorithm used for identification was identical to that used when quantifying Matrigel tube formation images (**Figure 4**).

To analyze the retinal vasculature, images were obtained from Odstrcilik et al [31] and the Pipeline code was adapted to only search/detect vasculature in the non-black areas of the image. No other modifications were made to the detection algorithm. Two representative analyses are shown in **Figure 8**.

## Discussion

This study describes the development and validation of an open-source software tool for analyzing TFAs. This tool was used to validate a novel *in vitro* model of hyperglycemia. There is a strong need for this type of software as the use of TFA analysis is widespread but no method exists for standardization (**Figure 1**). Additionally, analyzing TFA images is time consuming; a typical analysis requires a user to manually trace the tube network in each image. Most TFAs are quantified by measuring an image feature representing tube length, area, thickness, or branch points. The purpose of Pipeline is twofold; (1) to act as a tool that can reproducibly analyze images of *in vitro* tube formation assays and (2) to greatly reduce the time required to analyze TFAs by automating the process. Pipeline was designed to provide an analysis method that can be exactly replicated between images by implementation of a profile system that will store a set of optimized analysis parameters for a specific data set that can be saved for later retrieval (**Figure 4**).

When determining how a treatment affects the ability of endothelial cells to form tubes *in vitro*, is it essential that an author reports (1) how the measurements were quantified (2) the results of the quantification and (3) if the groups were significantly different. Documentation of these items affects interpretation of data, so it is crucial a description of these parameters is provided to the extent that the results can be repeated. These descriptions are even more important when images are quantified manually as inter- and intra-user variability can vary greatly within a study and negatively impact the results (**Table 3**). The Pipeline algorithm was written so the analysis can be perfectly reproduced by reporting values for the 7 algorithm analysis parameters (**Figure 4**).

Pipeline is the first open-source software package that can perform an automated analysis of *in vitro* TFAs. To our knowledge, it is the only software package (commercial or open-source) that can perform an automated analysis without using a fluorescent dye. This is beneficial as it decreases the cost of an experiment (additional reagents and software licenses). It also removes any interference that may be introduced when staining cells. A common method of TFA analysis is to simply threshold images for total tube area in an image. Error can be introduced when manually thresholding TFA images because differences in background brightness of TFAs occurs across an image when a Matrigel surface is not perfectly flat. The tube detection method within Pipeline is more robust than manually thresholding images for total tube area because the detection algorithm analyzes differences in pixels that are directly connected rather than assigning a global threshold to analyze the image against. Finally, Pipeline greatly decreases the time required when compared to manually analyzing images.

There are two limitations of the Pipeline software. The first is that the Pipeline algorithm is built using the edge detection algorithm referred to as the “Canny Method.” [34] The Canny Method detects object edges by finding areas with a high spatial derivative. Typically, this is not a problem as the majority of edges found in each image are tubes. The Canny Method does not exclude for debris or bubbles within the Matrigel. The cleanup steps following edge detection can exclude for debris/bubbles (**Figure 2**), however, the algorithm does not correct well for manufacturing defects occasionally observed in cell culture dishes (cracks, scratches, etc.). Additionally, for the Canny method to work optimally, an analyzed image must be in the TIFF format as compressed images save space by blunting high frequency intensity changes in adjacent pixels. The second limitation is that saturated pixels limit edge detection. When a pixel within an image is either purely white or black, this indicates that the detection limits of the camera were saturated. The true value of a saturated pixel is outside the dynamic range of the recorded value, so the spatial derivative calculated will be artificially low causing a possible edge pixel to be ignored. This problem is not unique to the Canny method, as saturated pixels can introduce problems when quantifying any image. [37] The best results will be obtained when using Pipeline to analyze TFA images with small amounts of debris and no saturated pixels.

Pipeline was validated by analyzing images of tubes formed *in vitro* on a Matrigel surface following exposure to simulated hyperglycemia on RCMVECs. Using Pipeline to analyze RCMVEC TFA images demonstrated a significant decrease in tube formation following 2 weeks of hyperglycemia (**Figure 7**). Hyperglycemia is a major factor associated with diabetes mellitus. [36], [38]–[41] Recently, *in vitro* hyperglycemic models have demonstrated the ability of hyperglycemia to induce endothelial cell dysfunction consistent with pathologies observed in T2D patients including, NFkB activation, inflammation, and reduced NO production. [39], [42]–[44] The results of this study are the first demonstration of hyperglycemia as a causative role in inhibiting angiogenesis in T2D. These results are important in conducting subsequent follow up studies to determine the mechanisms by which hyperglycemia inhibits angiogenesis.

Pipeline was specifically developed to analyze Matrigel TFAs; however, the detection algorithm has many more applications than this assay. The detection assay is well suited to detect and analyze structures that branch and are relatively thin. To demonstrate additional applications, the Pipeline algorithm was used to detect retinal vessels within a fundus photograph (**Figure 8**). [31] This application would be useful when quantifying retinal vasculature within DM patients as proliferative diabetic retinopathy (PDR), is responsible for vision loss. In PDR, retinal ischemia induces angiogenesis leading to retinal hemorrhage and detachment. [45] Automated quantification of retinal vasculature in DM patients could be used as a metric of relative risk of developing vision associated complications. In **Figure 8**, detection was performed with a high sensitivity/specificity on all areas of the image, with the exception of vessels within the optic disc. As the optic disc represents a very small portion of the image, the algorithm could easily be adapted to exclude vessels within this area when performing a high throughput analysis.

## Supporting Information

Table S1 **PubMed was queried for primary research articles that utilized Matrigel based tube formation assays and the following articles were returned.** The articles in this table were binned by year and counted to create Figure 1.
(PDF)
