# Supplementary material for: Automated Quantification Reveals Hyperglycemia Inhibits Endothelial Angiogenic Function
Source: PLoS One. 2014 Apr 9;9(4):e94599. doi: 10.1371/journal.pone.0094599 (PMC3981811; doi:10.1371/journal.pone.0094599)
Supplement: Table S1 — PubMed was queried for primary research articles that utilized Matrigel based tube formation assays and the following articles were returned. The articles in this table were binned by year and counted to create Figure 1. (PDF) [file pone.0094599.s001.pdf]

Supplemental Table 1: Studies Utilizing In Vitro Tube Formation Assay

Manuscript	Authors	Reference	Identifier
Newly synthesized quinazolinone HMJ-38 suppresses angiogenetic responses and triggers human umbilical vein endothelial cell apoptosis through p53-modulated Fas/death receptor signaling.	Chiang JH, Yang JS, Lu CC, Hour MJ, Chang SJ, Lee TH, Chung JG.	Toxicol Appl Pharmacol. 2013	PMID:23523585
KRC-408, a novel c-Met inhibitor, suppresses cell proliferation and angiogenesis of gastric cancer.	Hong SW, Jung KH, Park BH, Zheng HM, Lee HS, Choi MJ, Yun JI, Kang NS, Lee J, Hong SS.	Cancer Lett. 2013	PMID:23348694
Magnolol suppresses hypoxia-induced angiogenesis via inhibition of HIF-1Î±/VEGF signaling pathway in human bladder cancer cells.	Chen MC, Lee CF, Huang WH, Chou TC.	Biochem Pharmacol. 2013	PMID:23416116
Synergistic anticancer activity of HS-173, a novel PI3K inhibitor in combination with Sorafenib against pancreatic cancer cells.	Yun SM, Jung KH, Lee H, Son MK, Seo JH, Yan HH, Park BH, Hong S, Hong SS.	Cancer Lett. 2013	PMID:23340175
Discovery of PTPRJ agonist peptides which effectively inhibit in vitro cancer cell proliferation and tube formation.	Ortuso F, Paduano F, Carotenuto A, Gomez-Monterrey I, Bilotta A, Gaudio E, Sala M, Artese A, Vernieri E, Dattilo V, Iuliano R, Brancaccio D, Bertamino A, Musella S, Alcaro S, Grieco P, Perrotti N, Croce CM, Novellino E, Fusco A, Campiglia P, Trapasso F.	ACS Chem Biol. 2013	PMID:23627474
Evaluation of the in vitro and in vivo angiogenic effects of exendin-4.	Kang HM, Kang Y, Chun HJ, Jeong JW, Park C.	Biochem Biophys Res Commun. 2013	PMID:23541581
Xylitol inhibits in vitro and in vivo angiogenesis by suppressing the NF-ÎºB and Akt signaling pathways.	Yi EY, Kim YJ.	Int J Oncol. 2013	PMID:23615861
VEGFR2-dependent Angiogenic Capacity of Pericyte-like Dental Pulp Stem Cells.	Janebodin K, Zeng Y, Buranaphatthana W, Ieronimakis N, Reyes M.	J Dent Res. 2013	PMID:23609159
MicroRNA-106b-25 cluster targets Î²-TRCP2, increases the expression of Snail and enhances cell migration and invasion in H1299 (non small cell lung cancer) cells.	Savita U, Karunagaran D.	Biochem Biophys Res Commun. 2013	PMID:23611780
12-Deoxyphorbol 13-palmitate inhibit VEGF-induced angiogenesis via suppression of VEGFR-2-signaling pathway.	Xu HY, Pan YM, Chen ZW, Lin Y, Wang LH, Chen YH, Jie TT, Lu YY, Liu JC.	J Ethnopharmacol. 2013	PMID:23434607
Toluquinol, a marine fungus metabolite, is a new angiossuppresor that interferes the AKT pathway.	GarcÃa-Caballero M, Beffa MM, CaÃ±edo L, Medina MA, Quesada AR.	Biochem Pharmacol. 2013	PMID:23603293
Early Vessel Destabilization Mediated by Angiopoietin-2 and Subsequent Vessel Maturation via Angiopoietin-1 Induce Functional Neovasculature after Ischemia.	Qin D, Trenkwalder T, Lee S, Chillo O, Deindl E, Kupatt C, Hinkel R.	PLoS One. 2013	PMID:23613948 | PMCID:PMC3628915
Inherent phenotypic plasticity facilitates progression of head and neck cancer: endotheliod characteristics enable angiogenesis and invasion.	Tong M, Han BB, Holpuch AS, Pei P, He L, Mallery SR.	Exp Cell Res. 2013	PMID:23370231 | PMCID:PMC3602379
Endothelin-1 promotes vascular endothelial growth factor-dependent angiogenesis in human chondrosarcoma cells.	Wu MH, Huang CY, Lin JA, Wang SW, Peng CY, Cheng HC, Tang CH.	Oncogene. 2013	PMID:23584483
Anti-cancer effect of HS-345, a new tropomyosin-related kinase A inhibitor, on human pancreatic cancer.	Seo JH, Jung KH, Son MK, Yan HH, Ryu YL, Kim J, Lee JK, Hong S, Hong SS.	Cancer Lett. 2013	PMID:23587795
Characterization of in vivo chemoresistant human hepatocellular carcinoma cells with transendothelial differentiation capacities.	Marfels C, Hoehn M, Wagner E, GÃ¼nther M.	BMC Cancer. 2013	PMID:23547746 | PMCID:PMC3626554
Identification of human-selective analogues of the vascular-disrupting agent 5,6-dimethylxanthenone-4-acetic acid (DMXAA).	Tijono SM, Guo K, Henare K, Palmer BD, Wang LC, Albelda SM, Ching LM.	Br J Cancer. 2013	PMID:23481185 | PMCID:PMC3619269
ASS1 as a Novel Tumor Suppressor Gene in Myxofibrosarcoma: Aberrant loss via epigenetic DNA methylation confers aggressive phenotypes, negative prognostic impact, and therapeutic relevance.	Huang HY, Wu WR, Wang YH, Wang JW, Fang FM, Tsai JW, Li SH, Hung HC, Yu SC, Lan J, Shiue YL, Hsing CH, Chen LT, Li CF.	Clin Cancer Res. 2013	PMID:23549872
Dual Targeting of the Type 1 Insulin-like Growth Factor Receptor and its Ligands as an Effective Anti-angiogenic Strategy.	Bid HK, London CA, Gao J, Zhong H, Hollingsworth RE, Fernandez S, Mo X, Houghton PJ.	Clin Cancer Res. 2013	PMID:23549869
CHIP functions as a novel suppressor of tumour angiogenesis with prognostic significance in human gastric cancer.	Wang S, Wu X, Zhang J, Chen Y, Xu J, Xia X, He S, Qiang F, Li A, Shu Y, RÃ¸e OD, Li G, Zhou JW.	Gut. 2013	PMID:22535373
Antiangiogenesis Effects of Endostatin in Retinal Neovascularization.	Bai YJ, Huang LZ, Zhou AY, Zhao M, Yu WZ, Li XX.	J Ocul Pharmacol Ther. 2013	PMID:23545016

The biological effects and mechanisms of calcitonin gene-related peptide on human endothelial cell.	Tuo Y, Guo X, Zhang X, Wang Z, Zhou J, Xia L, Zhang Y, Wen J, Jin D.	J Recept Signal Transduct Res. 2013	PMID:23461295
Southern Brazilian autumnal propolis shows anti-angiogenic activity: An in vitro and in vivo study.	Meneghelli C, Joaquim LS, FÃ©lix GL, Somensi A, Tomazzoli M, da Silva DA, Berti FV, Veleirinho MB, Recouvreux DD, de Mattos Zeri AC, Dias PF, Maraschin M.	Microvasc Res. 2013	PMID:23538317
The C2238 Atrial Natriuretic Peptide Molecular Variant is Associated with Endothelial Damage and Dysfunction Through Natriuretic Peptide Receptor C Signaling.	Sciarretta S, Marchitti SS, Bianchi F, Moyes A, Barbato E, Di Castro S, Stanzione R, Cotugno M, Castello L, Calvieri C, Eberini I, Sadoshima J, Hobbs AJ, Volpe M, Rubattu S.	Circ Res. 2013	PMID:23529183
CXCR7/CXCR4/CXCL12 Axis Regulates the Proliferation, Migration, Survival and Tube Formation of Choroid-Retinal Endothelial Cells.	Jin J, Zhao WC, Yuan F.	Ophthalmic Res. 2013	PMID:23549077
Multicellular self-assembled spheroidal model of the blood brain barrier.	Urich E, Patsch C, Aigner S, Graf M, Iacone R, FreskgÃ¥rd PO.	Sci Rep. 2013	PMID:23511305 | PMCID:PMC3603320
Antiangiogenic activity and direct antitumor effect from a sulfated polysaccharide isolated from seaweed.	Guerra Dore CM, Faustino Alves MG, Santos ND, Cruz AK, CÃ¢mara RB, Castro AJ, GuimarÃ£es Alves L, Nader HB, Lisboa Leite E.	Microvasc Res. 2013	PMID:23507505
Juxtacrine and paracrine interactions of rat marrow-derived mesenchymal stem cells, muscle-derived satellite cells, and neonatal cardiomyocytes with endothelial cells in angiogenesis dynamics.	Rahbarghazi R, Nassiri SM, Khazraiinia P, Kajbafzadeh AM, Ahmadi SH, Mohammadi E, Molazem M, Zamani-Ahmadmahmudi M.	Stem Cells Dev. 2013	PMID:23072248 | PMCID:PMC3585743
Histone deacetylase 9 promotes angiogenesis by targeting the antiangiogenic microRNA-17-92 cluster in endothelial cells.	Kaluza D, Kroll J, Gesierich S, Manavski Y, Boeckel JN, Doebele C, Zelent A, RÃ¶ssig L, Zeiher AM, Augustin HG, Urbich C, Dimmeler S.	Arterioscler Thromb Vasc Biol. 2013	PMID:23288173
Proangiogenic features of Wharton's jelly-derived mesenchymal stromal/stem cells and their ability to form functional vessels.	Choi M, Lee HS, Naidansaren P, Kim HK, O E, Cha JH, Ahn HY, Yang PI, Shin JC, Joe YA.	Int J Biochem Cell Biol. 2013	PMID:23246593
Ceramide-1-phosphate regulates migration of multipotent stromal cells and endothelial progenitor cells--implications for tissue regeneration.	Kim C, Schneider G, Abdel-Latif A, Mierzejewska K, Sunkara M, Borkowska S, Ratajczak J, Morris AJ, Kucia M, Ratajczak MZ.	Stem Cells. 2013	PMID:23193025 | PMCID:PMC3582849
H-RN, a peptide derived from hepatocyte growth factor, inhibits corneal neovascularization by inducing endothelial apoptosis and arresting the cell cycle.	Sun Y, Su L, Wang Z, Xu Y, Xu X.	BMC Cell Biol. 2013	PMID:23433118 | PMCID:PMC3598793
S-nitrosoglutathione reductase (GSNOR) enhances vasculogenesis by mesenchymal stem cells.	Gomes SA, Rangel EB, Premer C, Dulce RA, Cao Y, Florea V, Balkan W, Rodrigues CO, Schally AV, Hare JM.	Proc Natl Acad Sci U S A. 2013	PMID:23288904 | PMCID:PMC3581904
The 106b~25 microRNA cluster is essential for neovascularization after hindlimb ischaemia in mice.	Semo J, Sharir R, Afek A, Avivi C, Barshack I, Maysel-Auslender S, Krelin Y, Kain D, Entin-Meer M, Keren G, George J.	Eur Heart J. 2013	PMID:23420866
Methyl jasmonate abolishes the migration, invasion and angiogenesis of gastric cancer cells through down-regulation of matrix metalloproteinase 14.	Zheng L, Li D, Xiang X, Tong L, Qi M, Pu J, Huang K, Tong Q.	BMC Cancer. 2013	PMID:23394613 | PMCID:PMC3576238
Inhibitory Effect of Adenoviral Vector-Mediated Delivery of p21(WAF1/CIP1) on Retinal Vascular Endothelial Cell Proliferation and Tube Formation in Cultured Rhesus Monkey Cells (RF/6A).	Han J, Yuan Z, Yan H.	Curr Eye Res. 2013	PMID:23383964
Synthesis and evaluation of the aldolase antibody-derived chemical-antibodies targeting Î±5Î²1 integrin.	Goswami RK, Liu Y, Liu C, Lerner RA, Sinha SC.	Mol Pharm. 2013	PMID:23102054 | PMCID:PMC3596504
Mesh-loosening quantification of inhibition of angiogenic tube formation through image analysis.	Minamikawa-Tachino R, Ogura K, Gotoh T.	Assay Drug Dev Technol. 2013	PMID:22994969
IPD-196, a novel phosphatidylinositol 3-kinase inhibitor with potent anticancer activity against hepatocellular carcinoma.	Lee JH, Lee H, Yun SM, Jung KH, Jeong Y, Yan HH, Hong S, Hong SS.	Cancer Lett. 2013	PMID:23142281
A novel imidazopyridine derivative, HS-106, induces apoptosis of breast cancer cells and represses angiogenesis by targeting the PI3K/mTOR pathway.	Li GY, Jung KH, Lee H, Son MK, Seo J, Hong SW, Jeong Y, Hong S, Hong SS.	Cancer Lett. 2013	PMID:23085493
CAPE suppresses VEGFR-2 activation, and tumor neovascularization and growth.	Chung TW, Kim SJ, Choi HJ, Kwak CH, Song KH, Suh SJ, Kim KJ, Ha KT, Park YG, Chang YC, Chang HW, Lee YC, Kim CH.	J Mol Med (Berl). 2013	PMID:22935775
Anti-angiogenic effects of lycopene through immunomodualtion of cytokine secretion in human peripheral blood mononuclear cells.	Huang CS, Chuang CH, Lo TF, Hu ML.	J Nutr Biochem. 2013	PMID:22704783
SMND-309 promotes angiogenesis in human umbilical vein endothelial cells through activating erythropoietin receptor/STAT3/VEGF pathways.	Du G, Zhu H, Yu P, Wang H, He J, Ye L, Fu F, Zhang J, Tian J.	Eur J Pharmacol. 2013	PMID:23276662

STAT1 activation by venous malformations mutant Tie2-R849W antagonizes VEGF-A-mediated angiogenic response partly via reduced bFGF production.	Huang YH, Wu MP, Pan SC, Su WC, Chen YW, Wu LW.	Angiogenesis. 2013	PMID:23086340
A novel role for inducible Fut2 in angiogenesis.	Tsou PS, Ruth JH, Campbell PL, Isozaki T, Lee S, Marotte H, Domino SE, Koch AE, Amin MA.	Angiogenesis. 2013	PMID:23065099
Interleukin-8 reduces post-surgical lymphedema formation by promoting lymphatic vessel regeneration.	Choi I, Lee YS, Chung HK, Choi D, Ecoiffier T, Lee HN, Kim KE, Lee S, Park EK, Maeng YS, Kim NY, Ladner RD, Petasis NA, Koh CJ, Chen L, Lenz HJ, Hong YK.	Angiogenesis. 2013	PMID:22945845
ADAM-10 is overexpressed in rheumatoid arthritis synovial tissue and mediates angiogenesis.	Isozaki T, Rabquer BJ, Ruth JH, Haines GK 3rd, Koch AE.	Arthritis Rheum. 2013	PMID:23124962
Differential roles of angiogenic chemokines in endothelial progenitor cell-induced angiogenesis.	Kanzler I, Tuchscheerer N, Steffens G, Simsekyilmaz S, Konschalla S, Kroh A, Simons D, Asare Y, Schober A, Bucala R, Weber C, Bernhagen J, Liehn EA.	Basic Res Cardiol. 2013	PMID:23184390
Combination of vinblastine and oncolytic herpes simplex virus vector expressing IL-12 therapy increases antitumor and antiangiogenic effects in prostate cancer models.	Passer BJ, Cheema T, Wu S, Wu CL, Rabkin SD, Martuza RL.	Cancer Gene Ther. 2013	PMID:23138870
HS-173, a novel phosphatidylinositol 3-kinase (PI3K) inhibitor, has anti-tumor activity through promoting apoptosis and inhibiting angiogenesis.	Lee H, Jung KH, Jeong Y, Hong S, Hong SS.	Cancer Lett. 2013	PMID:22929971
Title	Description	ShortDetails	Identifiers
Suppression of tumor proliferation and angiogenesis of hepatocellular carcinoma by HS-104, a novel phosphoinositide 3-kinase inhibitor.	Jung KH, Zheng HM, Jeong Y, Choi MJ, Lee H, Hong SW, Lee HS, Son MK, Lee S, Hong S, Hong SS.	Cancer Lett. 2013	PMID:22902995
Vitexicarpin acts as a novel angiogenesis inhibitor and its target network.	Zhang B, Liu L, Zhao S, Wang X, Liu L, Li S.	Evid Based Complement Alternat Med. 2013	PMID:23476684 | PMCID:PMC3583114
In Vitro Inhibition of Angiogenesis by Antibodies Directed against the 37kDa/67kDa Laminin Receptor.	Khusal R, Da Costa Dias B, Moodley K, Penny C, Reusch U, Knackmuss S, Little M, Weiss SF.	PLoS One. 2013	PMID:23554951 | PMCID:PMC3595224
Secretome of peripheral blood mononuclear cells enhances wound healing.	Mildner M, Hacker S, Haider T, Gschwandtner M, Werba G, Barresi C, Zimmermann M, Golabi B, Tschachler E, Ankersmit HJ.	PLoS One. 2013	PMID:23533667 | PMCID:PMC3606336
A dominant-negative FGF1 mutant (the R50E mutant) suppresses tumorigenesis and angiogenesis.	Mori S, Tran V, Nishikawa K, Kaneda T, Hamada Y, Kawaguchi N, Fujita M, Takada YK, Matsuura N, Zhao M, Takada Y.	PLoS One. 2013	PMID:23469107 | PMCID:PMC3585250
Three-dimensional neuroepithelial culture from human embryonic stem cells and its use for quantitative conversion to retinal pigment epithelium.	Zhu Y, Carido M, Meinhardt A, Kurth T, Karl MO, Ader M, Tanaka EM.	PLoS One. 2013	PMID:23358448 | PMCID:PMC3554725
Citicoline induces angiogenesis improving survival of vascular/human brain microvessel endothelial cells through pathways involving ERK1/2 and insulin receptor substrate-1.	Krupinski J, Abudawood M, Matou-Nasri S, Al-Baradie R, Petcu EB, Justicia C, Planas A, Liu D, Rovira N, Grau-Slevin M, Secades J, Slevin M.	Vasc Cell. 2012	PMID:23227823 | PMCID:PMC3554547
Folic acid inhibits endothelial cell proliferation through activating the cSrc/ERK 2/NF-Î°B/p53 pathway mediated by folic acid receptor.	Lin SY, Lee WR, Su YF, Hsu SP, Lin HC, Ho PY, Hou TC, Chou YP, Kuo CT, Lee WS.	Angiogenesis. 2012	PMID:22843228
Lactate stimulates angiogenesis and accelerates the healing of superficial and ischemic wounds in mice.	Porporato PE, Payen VL, De Saedeleer CJ, PrÃ©at V, Thissen JP, Feron O, Sonveaux P.	Angiogenesis. 2012	PMID:22660894
Effects of AFP-172 on COX-2-induced angiogenic activities on human umbilical vein endothelial cells.	Roh YJ, Park YG, Kang S, Kim SY, Moon JI.	Graefes Arch Clin Exp Ophthalmol. 2012	PMID:22910791
[Differentiation of human embryonic stem cells to endothelial cells via improved three-dimension approach].	Su WJ, Wang BY, Song XH, Wang LN, Liu YH, Zhou MQ, Tong LL, Li ZJ.	Zhongguo Yi Xue Ke Xue Yuan Xue Bao. 2012	PMID:23286395
Notch signalling pathways mediate synovial angiogenesis in response to vascular endothelial growth factor and angiopoietin 2.	Gao W, Sweeney C, Walsh C, Rooney P, McCormick J, Veale DJ, Fearon U.	Ann Rheum Dis. 2012	PMID:23161900
Mediterranean diet polyphenols reduce inflammatory angiogenesis through MMP-9 and COX-2 inhibition in human vascular endothelial cells: a potentially protective mechanism in atherosclerotic vascular disease and cancer.	Scoditti E, Calabriso N, Massaro M, Pellegrino M, Storelli C, Martines G, De Caterina R, Carluccio MA.	Arch Biochem Biophys. 2012	PMID:22595400
Antiangiogenic effects and mechanisms of trans-ethyl p-methoxycinnamate from Kaempferia galanga L.	He ZH, Yue GG, Lau CB, Ge W, But PP.	J Agric Food Chem. 2012	PMID:23106130
Orally active desulfated low molecular weight heparin and deoxycholic acid conjugate, 6ODS-	Hwang SR, Seo DH, Al-Hilal TA, Jeon OC, Kang JH, Kim	J Control Release.	PMID:23041275

LHbD, suppresses neovascularization and bone destruction in arthritis.	SH, Kim HS, Chang YT, Kang YM, Yang VC, Byun Y.	2012	
Antiangiogenesis and antioxidant activity of ethanol extracts of Pithecellobium jiringa.	Muslim NS, Nassar ZD, Aisha AF, Shafaei A, Idris N, Majid AM, Ismail Z.	BMC Complement Altern Med. 2012	PMID:23126282 | PMCID:PMC3522529
Inhibition of ocular neovascularization by a novel peptide derived from human placenta growth factor-1.	Zheng Y, Gu Q, Xu X.	Acta Ophthalmol. 2012	PMID:22994140
SB365 inhibits angiogenesis and induces apoptosis of hepatocellular carcinoma through modulation of PI3K/Akt/mTOR signaling pathway.	Hong SW, Jung KH, Lee HS, Choi MJ, Son MK, Zheng HM, Hong SS.	Cancer Sci. 2012	PMID:22909393
Monocyte chemotactic protein-induced protein (MCPIP) promotes inflammatory angiogenesis via sequential induction of oxidative stress, endoplasmic reticulum stress and autophagy.	Roy A, Kolattukudy PE.	Cell Signal. 2012	PMID:22820500
Inhibitory effects of Yangzheng Xiaoji on angiogenesis and the role of the focal adhesion kinase pathway.	Jiang WG, Ye L, Ji K, Frewer N, Ji J, Mason MD.	Int J Oncol. 2012	PMID:22971748
Betaine inhibits inÂ vitro and inÂ vivo angiogenesis through suppression of the NF-ÎºB and Akt signaling pathways.	Yi EY, Kim YJ.	Int J Oncol. 2012	PMID:22940742
Suppression of lymphangiogenesis in human lymphatic endothelial cells by simultaneously blocking VEGF-C and VEGF-D/VEGFR-3 with norcantharidin.	Liu ZY, Qiu HO, Yuan XJ, Ni YY, Sun JJ, Jing W, Fan YZ.	Int J Oncol. 2012	PMID:22922710
Effects of high glucose on human cavernous endothelial cells.	Ning H, Qiu X, Baine L, Lin G, Lue TF, Lin CS.	Urology. 2012	PMID:22951001
[Oleanolic acid inhibits proliferation of HUVECs, and inhibits migration and tube formation via VEGF pathway].	Wei JT, Liu M, Liu HZ, Zhao J, Xiao L, Han LJ, Lin XK.	Yao Xue Xue Bao. 2012	PMID:23387077
Endothelin-1 cooperates with hypoxia to induce vascular-like structures through vascular endothelial growth factor-C, -D and -A in lymphatic endothelial cells.	Garrafa E, Caprara V, Di Castro V, RosanÃ² L, Bagnato A, Spinella F.	Life Sci. 2012	PMID:22552325
Revascularization of Pancreatic Islet Allografts is Enhanced by Alpha-1-Antitrypsin Under Anti-inflammatory Conditions.	Bellacen K, Kalay N, Ozeri E, Shahaf G, Lewis EC.	Cell Transplant. 2012	PMID:23050776
Proangiogenic cell colonies grown in vitro from human peripheral blood mononuclear cells.	Mavromatis K, Sutcliffe DJ, Joseph G, Alexander RW, Waller EK, Quyyumi AA, Taylor WR.	J Biomol Screen. 2012	PMID:22904201
Up-regulation of miR-210 by vascular endothelial growth factor in ex vivo expanded CD34+ cells enhances cell-mediated angiogenesis.	Alaiti MA, Ishikawa M, Masuda H, Simon DI, Jain MK, Asahara T, Costa MA.	J Cell Mol Med. 2012	PMID:22360314
Peptides binding to prostate-specific antigen enhance its antiangiogenic activity.	Mattsson JM, NÃ¤rvÃ¤nen A, Stenman UH, Koistinen H.	Prostate. 2012	PMID:22430591
Umbilical cord blood-derived aldehyde dehydrogenase-expressing progenitor cells promote recovery from acute ischemic injury.	Putman DM, Liu KY, Broughton HC, Bell GI, Hess DA.	Stem Cells. 2012	PMID:22899443
Vixapatin (VP12), a c-type lectin-protein from Vipera xantina palestinae venom: characterization as a novel anti-angiogenic compound.	Momic T, Cohen G, Reich R, Arlinghaus FT, Eble JA, Marcinkiewicz C, Lazarovici P.	Toxins (Basel). 2012	PMID:23162702 | PMCID:PMC3496993
Notoginsenoside Ft1 promotes angiogenesis via HIF-1Î± mediated VEGF secretion and the regulation of PI3K/AKT and Raf/MEK/ERK signaling pathways.	Shen K, Ji L, Gong C, Ma Y, Yang L, Fan Y, Hou M, Wang Z.	Biochem Pharmacol. 2012	PMID:22771629
Mechanisms in decorin regulation of vascular endothelial growth factor-induced human trophoblast migration and acquisition of endothelial phenotype.	Lala N, Girish GV, Cloutier-Bosworth A, Lala PK.	Biol Reprod. 2012	PMID:22699486
c-Jun N-terminal kinase mediated VEGFR2 sustained phosphorylation is critical for VEGFA-induced angiogenesis in vitro and in vivo.	Shen K, Ji L, Lu B, Wang Z.	Cell Biochem Biophys. 2012	PMID:22592917
Functional consequences of prolactin signalling in endothelial cells: a potential link with angiogenesis in pathophysiology?	Reuwer AQ, Nowak-Sliwinska P, Mans LA, van der Loos CM, von der ThÃ¼sen JH, Twickler MT, Spek CA, Goffin V, Griffioen AW, Borensztajn KS.	J Cell Mol Med. 2012	PMID:22128761
Activation of AMP-activated protein kinase inhibits the proliferation of human endothelial cells.	Peyton KJ, Liu XM, Yu Y, Yates B, Durante W.	J Pharmacol Exp Ther. 2012	PMID:22700432 | PMCID:PMC3422516
Tissue factor-Akt signaling triggers microvessel formation.	Arderiu G, PeÃ±a E, Aledo R, Badimon L.	J Thromb Haemost. 2012	PMID:22784289
Characterization and comparison of embryonic stem cell-derived KDR+ cells with endothelial cells.	Sun X, Cheng L, Duan H, Lin G, Lu G.	Microvasc Res. 2012	PMID:22706170
The radiosensitivity of endothelial cells isolated from human breast cancer and normal tissue in vitro.	Park MT, Oh ET, Song MJ, Kim WJ, Cho YU, Kim SJ, Han JY, Suh JK, Choi EK, Lim BU, Song CW, Park HJ.	Microvasc Res. 2012	PMID:22705362
ADAMTS13 promotes angiogenesis and modulates VEGF-induced angiogenesis.	Lee M, Rodansky ES, Smith JK, Rodgers GM.	Microvasc Res. 2012	PMID:22626948

ADAMTS9 is a functional tumor suppressor through inhibiting AKT/mTOR pathway and associated with poor survival in gastric cancer.	Du W, Wang S, Zhou Q, Li X, Chu J, Chang Z, Tao Q, Ng EK, Fang J, Sung JJ, Yu J.	Oncogene. 2012	PMID:22907434
Role of the CCL21 and CCR7 pathways in rheumatoid arthritis angiogenesis.	Pickens SR, Chamberlain ND, Volin MV, Pope RM, Talarico NE, Mandelin AM 2nd, Shahrara S.	Arthritis Rheum. 2012	PMID:22392503 | PMCID:PMC3409328
HIF-1Î± Overexpression Induces Angiogenesis in Mesenchymal Stem Cells.	Razban V, Lotfi AS, Soleimani M, Ahmadi H, Massumi M, Khajeh S, Ghaedi M, Arjmand S, Najavand S, Khoshdel A.	Biores Open Access. 2012	PMID:23514846 | PMCID:PMC3559201
A novel vasculo-angiogenic effect of cilostazol mediated by cross-talk between multiple signalling pathways including the ERK/p38 MAPK signalling transduction cascade.	Chao TH, Tseng SY, Li YH, Liu PY, Cho CL, Shi GY, Wu HL, Chen JH.	Clin Sci (Lond). 2012	PMID:22339730
AMP-activated protein kinase mediates erythropoietin-induced activation of endothelial nitric oxide synthase.	Su KH, Yu YB, Hou HH, Zhao JF, Kou YR, Cheng LC, Shyue SK, Lee TS.	J Cell Physiol. 2012	PMID:22021095
Notch-1 mediates hypoxia-induced angiogenesis in rheumatoid arthritis.	Gao W, Sweeney C, Connolly M, Kennedy A, Ng CT, McCormick J, Veale DJ, Fearon U.	Arthritis Rheum. 2012	PMID:22275240
Radio-sensitivities and angiogenic signaling pathways of irradiated normal endothelial cells derived from diverse human organs.	Park MT, Oh ET, Song MJ, Lee H, Park HJ.	J Radiat Res. 2012	PMID:22843622 | PMCID:PMC3393354
Matrigel-based sprouting endothelial cell culture system from mouse corpus cavernosum is potentially useful for the study of endothelial and erectile dysfunction related to high-glucose exposure.	Yin GN, Ryu JK, Kwon MH, Shin SH, Jin HR, Song KM, Choi MJ, Kang DY, Kim WJ, Suh JK.	J Sex Med. 2012	PMID:22548733
Importance of interaction between nerve growth factor and Î±9Î²1 integrin in glial tumor angiogenesis.	Walsh EM, Kim R, Del Valle L, Weaver M, Sheffield J, Lazarovici P, Marcinkiewicz C.	Neuro Oncol. 2012	PMID:22611032 | PMCID:PMC3379805
Angiogenesis potential of human limbal stromal niche cells.	Li GG, Chen SY, Xie HT, Zhu YT, Tseng SC.	Invest Ophthalmol Vis Sci. 2012	PMID:22538425 | PMCID:PMC3374622
Title	Description	ShortDetails	Identifiers
Homocysteine-impaired angiogenesis is associated with VEGF/VEGFR inhibition.	Zhang Q, Li Q, Chen Y, Huang X, Yang IH, Cao L, Wu WK, Tan HM.	Front Biosci (Elite Ed). 2012	PMID:22652658
Effect of transient TCDD exposure on immortalized human trophoblast-derived cell lines.	Fukushima K, Tsukimori K, Li D, Takao T, Morokuma S, Kato K, Seki H, Takeda S, Matsumura S, Wake N.	Hum Exp Toxicol. 2012	PMID:22027506
Anti-angiogenic effect of furanodiene on HUVECs in vitro and on zebrafish in vivo.	Zhong ZF, Hoi PM, Wu GS, Xu ZT, Tan W, Chen XP, Cui L, Wu T, Wang YT.	J Ethnopharmacol. 2012	PMID:21911050
Lycopene inhibits angiogenesis both in vitro and in vivo by inhibiting MMP-2/uPA system through VEGFR2-mediated PI3K-Akt and ERK/p38 signaling pathways.	Chen ML, Lin YH, Yang CM, Hu ML.	Mol Nutr Food Res. 2012	PMID:22707264
Androgens inhibit tumor necrosis factor-Î±-induced cell adhesion and promote tube formation of human coronary artery endothelial cells.	Liao CH, Lin FY, Wu YN, Chiang HS.	Steroids. 2012	PMID:22504554
Enhanced endothelial differentiation of adipose-derived stem cells by substrate nanotopography.	Shi Z, Neoh KG, Kang ET, Poh CK, Wang W.	J Tissue Eng Regen Med. 2012	PMID:22628362
Blocking ephrinB2 with highly specific antibodies inhibits angiogenesis, lymphangiogenesis, and tumor growth.	AbÃ©ngozar MA, de Frutos S, Ferreiro S, Soriano J, Perez-Martinez M, Olmeda D, Marenchino M, CaÃ±amero M, Ortega S, Megias D, Rodriguez A, MartÃnez-Torrecuadrada JL.	Blood. 2012	PMID:22446484
Interleukin-1 assembles a proangiogenic signaling module consisting of caveolin-1, tumor necrosis factor receptor-associated factor 6, p38-mitogen-activated protein kinase (MAPK), and MAPK-activated protein kinase 2 in endothelial cells.	Jagielska J, Kapopara PR, Salguero G, Scherr M, SchÃ¼tt H, Grote K, Schieffer B, Bavendiek U.	Arterioscler Thromb Vasc Biol. 2012	PMID:22345171
Arenobufagin, a bufadienolide compound from toad venom, inhibits VEGF-mediated angiogenesis through suppression of VEGFR-2 signaling pathway.	Li M, Wu S, Liu Z, Zhang W, Xu J, Wang Y, Liu J, Zhang D, Tian H, Li Y, Ye W.	Biochem Pharmacol. 2012	PMID:22305746
Antiangiogenic mechanisms of PJ-8, a novel inhibitor of vascular endothelial growth factor receptor signaling.	Huang SW, Lien JC, Kuo SC, Huang TF.	Carcinogenesis. 2012	PMID:22436611
Crosstalk between oligodendrocytes and cerebral endothelium contributes to vascular remodeling after white matter injury.	Pham LD, Hayakawa K, Seo JH, Nguyen MN, Som AT, Lee BJ, Guo S, Kim KW, Lo EH, Arai K.	Glia. 2012	PMID:22392631 | PMCID:PMC3325331
Norcantharidin: a potential antiangiogenic agent for gallbladder cancers in vitro and in vivo.	Zhang JT, Fan YZ, Chen CQ, Zhao ZM, Sun W.	Int J Oncol. 2012	PMID:22200632
Pro-angiogenic effects of resveratrol in brain endothelial cells: nitric oxide-mediated regulation of vascular endothelial growth factor and metalloproteinases.	SimÃ£o F, Pagnussat AS, Seo JH, Navaratna D, Leung W, Lok J, Guo S, Waeber C, Salbego CG, Lo EH.	J Cereb Blood Flow Metab. 2012	PMID:22314268 | PMCID:PMC3345913
Identification of IGPR-1 as a novel adhesion molecule involved in angiogenesis.	Rahimi N, Rezazadeh K, Mahoney JE, Hartsough E, Meyer RD.	Mol Biol Cell. 2012	PMID:22419821 | PMCID:PMC3338432

Enhancement of radiation response with bevacizumab.	Hoang T, Huang S, Armstrong E, Eickhoff JC, Harari PM.	J Exp Clin Cancer Res. 2012	PMID:22538017 | PMCID:PMC3537546
Suppression of endothelial cell activity by inhibition of TNFÎ±.	Shu Q, Amin MA, Ruth JH, Campbell PL, Koch AE.	Arthritis Res Ther. 2012	PMID:22534470 | PMCID:PMC3446462
In vitro Anti-Angiogenesis Effects and Active Constituents of the Saponin Fraction From Gleditsia sinensis.	Lu D, Xia Y, Tong B, Zhang C, Pan R, Xu H, Yang X, Dai Y.	Integr Cancer Ther. 2012	PMID:22505594
Rudhira/BCAS3 is a cytoskeletal protein that controls Cdc42 activation and directional cell migration during angiogenesis.	Jain M, Bhat GP, Vijayraghavan K, Inamdar MS.	Exp Cell Res. 2012	PMID:22300583
Aldosterone inhibits endothelial morphogenesis and angiogenesis through the downregulation of vascular endothelial growth factor receptor-2 expression subsequent to peroxisome proliferator-activated receptor gamma.	Fujii M, Inoki I, Saga M, Morikawa N, Arakawa K, Inaba S, Yoshioka K, Konoshita T, Miyamori I.	J Steroid Biochem Mol Biol. 2012	PMID:22212769
In vitro and in vivo antiangiogenic properties of the serpin protease nexin-1.	Selbonne S, Azibani F, Iatmanen S, Boulaftali Y, Richard B, Jandrot-Perrus M, Bouton MC, Arocas V.	Mol Cell Biol. 2012	PMID:22331468 | PMCID:PMC3318585
HS-116, a novel phosphatidylinositol 3-kinase inhibitor induces apoptosis and suppresses angiogenesis of hepatocellular carcinoma through inhibition of the PI3K/AKT/mTOR pathway.	Jung KH, Choi MJ, Hong S, Lee H, Hong SW, Zheng HM, Lee HS, Hong S, Hong SS.	Cancer Lett. 2012	PMID:22182943
Implication of AMP-activated protein kinase in transient receptor potential vanilloid type 1-mediated activation of endothelial nitric oxide synthase.	Ching LC, Chen CY, Su KH, Hou HH, Shyue SK, Kou YR, Lee TS.	Mol Med. 2012	PMID:22451268
Methyl tert butyl ether is anti-angiogenic in both in vitro and in vivo mammalian model systems.	Kozlosky J, Bonventre J, Cooper K.	J Appl Toxicol. 2012	PMID:22407988
Exosomes released by K562 chronic myeloid leukemia cells promote angiogenesis in a Src-dependent fashion.	Mineo M, Garfield SH, Taverna S, Flugy A, De Leo G, Alessandro R, Kohn EC.	Angiogenesis. 2012	PMID:22203239 | PMCID:PMC3595015
An indirubin derivative, E804, exhibits potent angiosuppressive activity.	Chan YK, Kwok HH, Chan LS, Leung KS, Shi J, Mak NK, Wong RN, Yue PY.	Biochem Pharmacol. 2012	PMID:22178720
Amniotic mesenchymal stem cells have robust angiogenic properties and are effective in treating hindlimb ischaemia.	Kim SW, Zhang HZ, Kim CE, An HS, Kim JM, Kim MH.	Cardiovasc Res. 2012	PMID:22155484
Inhibition of mini-TyrRS-induced angiogenesis response in endothelial cells by VE-cadherin-dependent mini-TrpRS.	Zeng R, Chen YC, Zeng Z, Liu XX, Liu R, Qiang O, Li X.	Heart Vessels. 2012	PMID:21442253
An experimental study of a modified dahuang zhechong pill on the--angiogenesis of RF/6A cells in vitro.	Luo XS, Wu XW, Gu Q.	J Tradit Chin Med. 2012	PMID:22594107
HMGB1 promotes lymphangiogenesis of human lymphatic endothelial cells in vitro.	Qiu Y, Chen Y, Fu X, Zhang L, Tian J, Hao Q.	Med Oncol. 2012	PMID:21181308
CREB-binding protein silencing inhibits thrombin-induced endothelial progenitor cells angiogenesis.	Jiang H, Chen SS, Yang J, Chen J, He B, Zhu LH, Wang L.	Mol Biol Rep. 2012	PMID:21670961
Potent inhibition of angiogenesis by the IGF-1 receptor-targeting antibody SCH717454 is reversed by IGF-2.	Bid HK, Zhan J, Phelps DA, Kurmasheva RT, Houghton PJ.	Mol Cancer Ther. 2012	PMID:22188815 | PMCID:PMC3421238
Pro-angiogenic activity of astragaloside IV in HUVECs in vitro and zebrafish in vivo.	Zhang Y, Hu G, Li S, Li ZH, Lam CO, Hong SJ, Kwan YW, Chan SW, Leung GP, Lee SM.	Mol Med Rep. 2012	PMID:22179585
hESC derived neuro-epithelial rosettes recapitulate early mammalian neurulation events; an in vitro model.	Curchoe CL, Russo J, Terskikh AV.	Stem Cell Res. 2012	PMID:22265743
Effects of resistin-like molecule Î² over-expression on gastric cancer cells in vitro.	Zheng LD, Yang CL, Qi T, Qi M, Tong L, Tong QS.	World J Gastroenterol. 2012	PMID:22371635 | PMCID:PMC3286138
Angiogenesis induced by signal transducer and activator of transcription 5A (STAT5A) is dependent on autocrine activity of proliferin.	Yang X, Qiao D, Meyer K, Pier T, Keles S, Friedl A.	J Biol Chem. 2012	PMID:22199350 | PMCID:PMC3307309
9-cis retinoic acid promotes lymphangiogenesis and enhances lymphatic vessel regeneration: therapeutic implications of 9-cis retinoic acid for secondary lymphedema.	Choi I, Lee S, Kyoung Chung H, Suk Lee Y, Eui Kim K, Choi D, Park EK, Yang D, Ecoiffier T, Monahan J, Chen W, Aguilar B, Lee HN, Yoo J, Koh CJ, Chen L, Wong AK, Hong YK.	Circulation. 2012	PMID:22275501 | PMCID:PMC3327127
Transient receptor potential canonical channels are required for in vitro endothelial tube formation.	Antigny F, Girardin N, Frieden M.	J Biol Chem. 2012	PMID:22203682 | PMCID:PMC3285360
The recombinant lectin-like domain of thrombomodulin inhibits angiogenesis through interaction with Lewis Y antigen.	Kuo CH, Chen PK, Chang BI, Sung MC, Shi CS, Lee JS, Chang CF, Shi GY, Wu HL.	Blood. 2012	PMID:22101897
Fisetin inhibits various attributes of angiogenesis in vitro and in vivo--implications for angioprevention.	Bhat TA, Nambiar D, Pal A, Agarwal R, Singh RP.	Carcinogenesis. 2012	PMID:22139440

Title	Description	ShortDetails	Identifiers
Synthesis and biological evaluation of novel indolocarbazoles with anti-angiogenic activity.	Acero N, BraÃ±a MF, AÃ±orbe L, DomÃnguez G, MuÃ±oz-Mingarro D, Mitjans F, Piulats J.	Eur J Med Chem. 2012	PMID:22182929
Apoptotic and anti-angiogenic effects of Pulsatilla koreana extract on hepatocellular carcinoma.	Hong SW, Jung KH, Lee HS, Choi MJ, Zheng HM, Son MK, Lee GY, Hong SS.	Int J Oncol. 2012	PMID:21935571
Adenovirus-mediated delivery of CALR and MAGE-A3 inhibits invasion and angiogenesis of glioblastoma cell line U87.	Liu XL, Zhao D, Sun DP, Wang Y, Li Y, Qiu FQ, Ma P.	J Exp Clin Cancer Res. 2012	PMID:22293781 | PMCID:PMC3337230
ADAM17 promotes glioma cell malignant phenotype.	Zheng X, Jiang F, Katakowski M, Lu Y, Chopp M.	Mol Carcinog. 2012	PMID:21480393 | PMCID:PMC3234333
In vitro differentiation of human skin-derived multipotent stromal cells into putative endothelial-like cells.	Vishnubalaji R, Manikandan M, Al-Nbaheen M, Kadalmani B, Aldahmash A, Alajez NM.	BMC Dev Biol. 2012	PMID:22280443 | PMCID:PMC3280173
Induction of vascular progenitor cells from endothelial cells stimulates coronary collateral growth.	Yin L, Ohanyan V, Pung YF, Delucia A, Bailey E, Enrick M, Stevanov K, Kolz CL, Guarini G, Chilian WM.	Circ Res. 2012	PMID:22095729
The expression and function of vascular endothelial growth factor in retinal pigment epithelial (RPE) cells is regulated by 4-hydroxynonenal (HNE) and glutathione S-transferaseA4-4.	Vatsyayan R, Lelsani PC, Chaudhary P, Kumar S, Awasthi S, Awasthi YC.	Biochem Biophys Res Commun. 2012	PMID:22155253 | PMCID:PMC3259230
N-terminal domain of soluble epoxide hydrolase negatively regulates the VEGF-mediated activation of endothelial nitric oxide synthase.	Hou HH, Hammock BD, Su KH, Morisseau C, Kou YR, Imaoka S, Oguro A, Shyue SK, Zhao JF, Lee TS.	Cardiovasc Res. 2012	PMID:22072631 | PMCID:PMC3243038
Identification of cancer stem cells in vincristine preconditioned SGC7901 gastric cancer cell line.	Xue Z, Yan H, Li J, Liang S, Cai X, Chen X, Wu Q, Gao L, Wu K, Nie Y, Fan D.	J Cell Biochem. 2012	PMID:21913215
Protective actions of globular and full-length adiponectin on human endothelial cells: novel insights into adiponectin-induced angiogenesis.	Adya R, Tan BK, Chen J, Randeva HS.	J Vasc Res. 2012	PMID:22964477
Low concentration of S100A8/9 promotes angiogenesis-related activity of vascular endothelial cells: bridges among inflammation, angiogenesis, and tumorigenesis?	Li C, Li S, Jia C, Yang L, Song Z, Wang Y.	Mediators Inflamm. 2012	PMID:22685372 | PMCID:PMC3363068
Title	Description	ShortDetails	Identifiers
Bp5250 inhibits vascular endothelial growth factor-induced angiogenesis and HIF-1Î± expression on endothelial cells.	Lin KT, Lien JC, Chung CH, Kuo SC, Huang TF.	Naunyn Schmiedebergs Arch Pharmacol. 2012	PMID:21947252
Evaluation of antiangiogenic and antoxidant properties of Parkia speciosa Hassk extracts.	Aisha AF, Abu-Salah KM, Alrokayan SA, Ismail Z, Abdulmajid AM.	Pak J Pharm Sci. 2012	PMID:22186303
Effects of urotensin II on functional activity of late endothelial progenitor cells.	Yi K, Yu M, Wu L, Tan X.	Peptides. 2012	PMID:22123628
Luteolin inhibits human prostate tumor growth by suppressing vascular endothelial growth factor receptor 2-mediated angiogenesis.	Pratheeshkumar P, Son YO, Budhraja A, Wang X, Ding S, Wang L, Hitron A, Lee JC, Kim D, Divya SP, Chen G, Zhang Z, Luo J, Shi X.	PLoS One. 2012	PMID:23300633 | PMCID:PMC3534088
The epidermal growth factor-like domain of CD93 is a potent angiogenic factor.	Kao YC, Jiang SJ, Pan WA, Wang KC, Chen PK, Wei HJ, Chen WS, Chang BI, Shi GY, Wu HL.	PLoS One. 2012	PMID:23272129 | PMCID:PMC3525571
Effect of lumican on the migration of human mesenchymal stem cells and endothelial progenitor cells: involvement of matrix metalloproteinase-14.	Malinowski M, Pietraszek K, Perreau C, Boguslawski M, Decot V, Stoltz JF, Vallar L, Niewiarowska J, Cierniewski C, Maquart FX, Wegrowski Y, BrÃ©zillon S.	PLoS One. 2012	PMID:23236386 | PMCID:PMC3517548
Development of a novel DNA aptamer ligand targeting to primary cultured tumor endothelial cells by a cell-based SELEX method.	Ara MN, Hyodo M, Ohga N, Hida K, Harashima H.	PLoS One. 2012	PMID:23226512 | PMCID:PMC3514264
TLR2 and TLR4 mediate differential responses to limb ischemia through MyD88-dependent and independent pathways.	Sachdev U, Cui X, McEnaney R, Wang T, Benabou K, Tzeng E.	PLoS One. 2012	PMID:23209800 | PMCID:PMC3510193
ARTEMIN promotes de novo angiogenesis in ER negative mammary carcinoma through activation of TWIST1-VEGF-A signalling.	Banerjee A, Wu ZS, Qian PX, Kang J, Liu DX, Zhu T, Lobie PE.	PLoS One. 2012	PMID:23185544 | PMCID:PMC3503764
Quercetin inhibits angiogenesis mediated human prostate tumor growth by targeting VEGFR- 2 regulated AKT/mTOR/P70S6K signaling pathways.	Pratheeshkumar P, Budhraja A, Son YO, Wang X, Zhang Z, Ding S, Wang L, Hitron A, Lee JC, Xu M, Chen G, Luo J, Shi X.	PLoS One. 2012	PMID:23094058 | PMCID:PMC3475699
Calpain/SHP-1 interaction by honokiol dampening peritoneal dissemination of gastric cancer in nu/nu mice.	Liu SH, Wang KB, Lan KH, Lee WJ, Pan HC, Wu SM, Peng YC, Chen YC, Shen CC, Cheng HC, Liao KK, Sheu ML.	PLoS One. 2012	PMID:22937084 | PMCID:PMC3427156
n-Butyl benzyl phthalate promotes breast cancer progression by inducing expression of lymphoid enhancer factor 1.	Hsieh TH, Tsai CF, Hsu CY, Kuo PL, Hsi E, Suen JL, Hung CH, Lee JN, Chai CY, Wang SC, Tsai EM.	PLoS One. 2012	PMID:22905168 | PMCID:PMC3414447

An IP-10 (CXCL10)-derived peptide inhibits angiogenesis.	Yates-Binder CC, Rodgers M, Jaynes J, Wells A, Bodnar RJ, Turner T.	PLoS One. 2012	PMID:22815829 | PMCID:PMC3397949
Effects of simulated microgravity on human umbilical vein endothelial cell angiogenesis and role of the PI3K-Akt-eNOS signal pathway.	Shi F, Wang YC, Zhao TZ, Zhang S, Du TY, Yang CB, Li YH, Sun XQ.	PLoS One. 2012	PMID:22808143 | PMCID:PMC3396652
In vitro and in vivo anti-angiogenic activities of Panduratin A.	Lai SL, Cheah SC, Wong PF, Noor SM, Mustafa MR.	PLoS One. 2012	PMID:22666456 | PMCID:PMC3364190
The EYA tyrosine phosphatase activity is pro-angiogenic and is inhibited by benzbromarone.	Tadjuidje E, Wang TS, Pandey RN, Sumanas S, Lang RA, Hegde RS.	PLoS One. 2012	PMID:22545090 | PMCID:PMC3335822
Anti-angiogenic activity of a small molecule STAT3 inhibitor LLL12.	Bid HK, Oswald D, Li C, London CA, Lin J, Houghton PJ.	PLoS One. 2012	PMID:22530037 | PMCID:PMC3328460
Phloroglucinol inhibits the bioactivities of endothelial progenitor cells and suppresses tumor angiogenesis in LLC-tumor-bearing mice.	Kwon YH, Jung SY, Kim JW, Lee SH, Lee JH, Lee BY, Kwon SM.	PLoS One. 2012	PMID:22496756 | PMCID:PMC3322124
Dehydrocostuslactone suppresses angiogenesis in vitro and in vivo through inhibition of Akt/GSK-3Î² and mTOR signaling pathways.	Wang CY, Tsai AC, Peng CY, Chang YL, Lee KH, Teng CM, Pan SL.	PLoS One. 2012	PMID:22359572 | PMCID:PMC3281050
[Ghrelin stimulates in vitro angiogenic capacity of rat cardiac microvascular endothelial cells].	Wang L, Chen QW, Li GQ, Ke DZ.	Zhonghua Xin Xue Guan Bing Za Zhi. 2012	PMID:22490634
Nerve growth factor induces cord formation of mesenchymal stem cell by promoting proliferation and activating the PI3K/Akt signaling pathway.	Wang WX, Hu XY, Xie XJ, Liu XB, Wu RR, Wang YP, Gao F, Wang JA.	Acta Pharmacol Sin. 2011	PMID:22139028
Examining the role of Rac1 in tumor angiogenesis and growth: a clinically relevant RNAi-mediated approach.	Vader P, van der Meel R, Symons MH, Fens MH, Pieters E, Wilschut KJ, Storm G, Jarzabek M, Gallagher WM, Schiffelers RM, Byrne AT.	Angiogenesis. 2011	PMID:21789714
Suppression of neovascularization and experimental arthritis by D-form of anti-flt-1 peptide conjugated with mini-PEG(â„¢).	Kong JS, Yoo SA, Kang JH, Ko W, Jeon S, Chae CB, Cho CS, Kim WU.	Angiogenesis. 2011	PMID:21751011
Î² Common receptor integrates the erythropoietin signaling in activation of endothelial nitric oxide synthase.	Su KH, Shyue SK, Kou YR, Ching LC, Chiang AN, Yu YB, Chen CY, Pan CC, Lee TS.	J Cell Physiol. 2011	PMID:21321940
Anti-angiogenic action of PPARÎ³ ligand in human umbilical vein endothelial cells is mediated by PTEN upregulation and VEGFR-2 downregulation.	Kim KY, Ahn JH, Cheon HG.	Mol Cell Biochem. 2011	PMID:21769449
Effects of plasminogen activator inhibitor-1-specific RNA aptamers on cell adhesion, motility, and tube formation.	Brandal S, Blake CM, Sullenger BA, Fortenberry YM.	Nucleic Acid Ther. 2011	PMID:22103403 | PMCID:PMC3279719
Increased hyaluronan fragmentation during pulmonary ischemia.	Eldridge L, Moldobaeva A, Wagner EM.	Am J Physiol Lung Cell Mol Physiol. 2011	PMID:21821727 | PMCID:PMC3213986
Interleukin-17A induction of angiogenesis, cell migration, and cytoskeletal rearrangement.	Moran EM, Connolly M, Gao W, McCormick J, Fearon U, Veale DJ.	Arthritis Rheum. 2011	PMID:21834066
C086, a novel analog of curcumin, induces growth inhibition and down-regulation of NFÎºB in colon cancer cells and xenograft tumors.	Chen C, Liu Y, Chen Y, Xu J.	Cancer Biol Ther. 2011	PMID:21900746
Xiongshao capsule promotes angiogenesis of HUVEC via enhancing cell proliferation and up-regulating the expression of bFGF and VEGF.	Lin JM, Zhao JY, Zhuang QC, Hong ZF, Peng J.	Chin J Integr Med. 2011	PMID:22057413
A novel in vitro angiogenesis model based on a microfluidic device.	Xiaozhen D, Shaoxi C, Qunfang Y, Jiahuan J, Xiaoqing Y, Xin X, Qifeng J, Albert Chih-Lueh W, Yi T.	Chin Sci Bull. 2011	PMID:22247609 | PMCID:PMC3254117
Modulation of endothelial cell proliferation and capillary network formation by the ox-LDL component: 1-palmitoyl-2-archidonoyl-sn-glycero-3-phosphocholine (ox-PAPC).	Kiec-Wilk B, Polus A, Razny U, Cialowicz U, Dembinska-Kiec A.	Genes Nutr. 2011	PMID:21484165 | PMCID:PMC3197846
MicroRNA-29b suppresses tumor angiogenesis, invasion, and metastasis by regulating matrix metalloproteinase 2 expression.	Fang JH, Zhou HC, Zeng C, Yang J, Liu Y, Huang X, Zhang JP, Guan XY, Zhuang SM.	Hepatology. 2011	PMID:21793034
Overexpression of the oncostatin M receptor in cervical squamous cell carcinoma cells is associated with a pro-angiogenic phenotype and increased cell motility and invasiveness.	Winder DM, Chattopadhyay A, Muralidhar B, Bauer J, English WR, Zhang X, Karagavriilidou K, Roberts I, Pett MR, Murphy G, Coleman N.	J Pathol. 2011	PMID:21952923
Enzymatic activity of free-prostate-specific antigen (f-PSA) is not required for some of its physiological activities.	Chadha KC, Nair BB, Chakravarthi S, Zhou R, Godoy A, Mohler JL, Aalinkeel R, Schwartz SA, Smith GJ.	Prostate. 2011	PMID:21446007
Regeneration of three layers vascular wall by using BMP2-treated MSC involving HIF-1Î± and Id1 expressions through JAK/STAT pathways.	Belmokhtar K, Bourguignon T, Worou ME, Khamis G, Bonnet P, Domenech J, Eder V.	Stem Cell Rev. 2011	PMID:21472453
Lumican inhibits angiogenesis by interfering with Î±2Î²1 receptor activity and downregulating	Niewiarowska J, BrÃ©zillon S, Sacewicz-Hofman I,	Thromb Res. 2011	PMID:21752432

MMP-14 expression.	Bednarek R, Maquart FX, Malinowski M, Wiktorska M, Wegrowski Y, Cierniewski CS.		
Hydrogen sulphide and angiogenesis: mechanisms and applications.	SzabÃ³ C, Papapetropoulos A.	Br J Pharmacol. 2011	PMID:21198548 | PMCID:PMC3195910
A disalicylic acid-furanyl derivative inhibits ephrin binding to a subset of Eph receptors.	Noberini R, De SK, Zhang Z, Wu B, Raveendra-Panickar D, Chen V, Vazquez J, Qin H, Song J, Cosford ND, Pellecchia M, Pasquale EB.	Chem Biol Drug Des. 2011	PMID:21791013 | PMCID:PMC3196665
Inhibitory effects of SY0916, a platelet-activating factor receptor antagonist, on the angiogenesis of human umbilical vascular endothelial cells.	Wu LQ, Ouyang XY, Liu Y, Peng SY, Wang L, Wang WJ.	J Asian Nat Prod Res. 2011	PMID:21985181
P-450-dependent epoxygenase pathway of arachidonic acid is involved in myeloma-induced angiogenesis of endothelial cells.	Shao J, Li Q, Wang H, Liu F, Jiang J, Zhu X, Chen Z, Zou P.	J Huazhong Univ Sci Technolog Med Sci. 2011	PMID:22038346
Glioblastoma-dependent differentiation and angiogenic potential of human mesenchymal stem cells in vitro.	Birnbaum T, Hildebrandt J, Nuebling G, Sostak P, Straube A.	J Neurooncol. 2011	PMID:21547397
Over-expression of sphingosine kinase-1 enhances a progenitor phenotype in human endothelial cells.	Barrett JM, Parham KA, Pippal JB, Cockshell MP, Moretti PA, Brice SL, Pitson SM, Bonder CS.	Microcirculation. 2011	PMID:21672077
The inhibitory effects of 5-hydroxy-3,6,7,8,3',4'-hexamethoxyflavone on human colon cancer cells.	Qiu P, Guan H, Dong P, Guo S, Zheng J, Li S, Chen Y, Ho CT, Pan MH, McClements DJ, Xiao H.	Mol Nutr Food Res. 2011	PMID:21648071 | PMCID:PMC3449327
[Effect of endothelium-specific deletion of PTEN on hemangioblast development in mouse embryo AGM region].	Gao J, Yao HY, Liang XL, Wang XY, Wu Y, Liu YL, Mao N.	Zhongguo Shi Yan Xue Ye Xue Za Zhi. 2011	PMID:22040977
Inhibition of angiogenesis involves in anticancer activity of riccardin D, a macrocyclic bisbibenzyl, in human lung carcinoma.	Sun CC, Zhang YS, Xue X, Cheng YN, Liu HP, Zhao CR, Lou HX, Qu XJ.	Eur J Pharmacol. 2011	PMID:21704029
Exosomes from human CD34(+) stem cells mediate their proangiogenic paracrine activity.	Sahoo S, Klychko E, Thorne T, Misener S, Schultz KM, Millay M, Ito A, Liu T, Kamide C, Agrawal H, Perlman H, Qin G, Kishore R, Losordo DW.	Circ Res. 2011	PMID:21835908 | PMCID:PMC3201702
Vascular tube formation and angiogenesis induced by polyvinylpyrrolidone-coated silver nanoparticles.	Kang K, Lim DH, Choi IH, Kang T, Lee K, Moon EY, Yang Y, Lee MS, Lim JS.	Toxicol Lett. 2011	PMID:21729742
A Matrigel-based tube formation assay to assess the vasculogenic activity of tumor cells.	Francescone RA 3rd, Faibish M, Shao R.	J Vis Exp. 2011	PMID:21931289
Nuclear translocation of phosphorylated STAT3 regulates VEGF-A-induced lymphatic endothelial cell migration and tube formation.	Okazaki H, Tokumaru S, Hanakawa Y, Shiraishi K, Shirakata Y, Dai X, Yang L, Tohyama M, Hashimoto K, Sayama K.	Biochem Biophys Res Commun. 2011	PMID:21835168
Title	Description	ShortDetails	Identifiers
Opposing effects of monomeric and pentameric C-reactive protein on endothelial progenitor cells.	Ahrens I, Domeij H, Eisenhardt SU, Topcic D, Albrecht M, Leitner E, Viitaniemi K, Jowett JB, Lappas M, Bode C, Haviv I, Peter K.	Basic Res Cardiol. 2011	PMID:21562922 | PMCID:PMC3149664
18Î±-glycyrrhetinic acid targets prostate cancer cells by down-regulating inflammation-related genes.	Shetty AV, Thirugnanam S, Dakshinamoorthy G, Samykutty A, Zheng G, Chen A, Bosland MC, Kajdacsy-Balla A, Gnanasekar M.	Int J Oncol. 2011	PMID:21637916
Antiangiogenesis agents avastin and erbitux enhance the efficacy of photodynamic therapy in a murine bladder tumor model.	Bhuvaneswari R, Yuen GY, Chee SK, Olivo M.	Lasers Surg Med. 2011	PMID:22057493
LYP, a bestatin dimethylaminoethyl ester, inhibited cancer angiogenesis both in vitro and in vivo.	Gao JJ, Xue X, Gao ZH, Cui SX, Cheng YN, Xu WF, Tang W, Qu XJ.	Microvasc Res. 2011	PMID:21664364
Docosahexaenoic acid stimulates tube formation in first trimester trophoblast cells, HTR8/SVneo.	Johnsen GM, Basak S, Weedon-FekjÃ¦r MS, Staff AC, Duttaroy AK.	Placenta. 2011	PMID:21741084
Antagonistic VEGF variants engineered to simultaneously bind to and inhibit VEGFR2 and alphavbeta3 integrin.	Papo N, Silverman AP, Lahti JL, Cochran JR.	Proc Natl Acad Sci U S A. 2011	PMID:21825147 | PMCID:PMC3161552
Spatiotemporal expression of SERPINE2 in the human placenta and its role in extravillous trophoblast migration and invasion.	Chern SR, Li SH, Chiu CL, Chang HH, Chen CP, Tsuen Chen EI.	Reprod Biol Endocrinol. 2011	PMID:21806836 | PMCID:PMC3161939
Tissue-engineered bone formation using periosteal-derived cells and polydioxanone/pluronic F127 scaffold with pre-seeded adipose tissue-derived CD146 positive endothelial-like cells.	Lee JH, Kim JH, Oh SH, Kim SJ, Hah YS, Park BW, Kim DR, Rho GJ, Maeng GH, Jeon RH, Lee HC, Kim JR, Kim GC, Kim UK, Byun JH.	Biomaterials. 2011	PMID:21543114
Prolyl oligopeptidase induces angiogenesis both in vitro and in vivo in a novel regulatory manner.	MyÃ¶hÃ¤nen TT, Tenorio-Laranga J, Jokinen B, VÃ¡zquez-	Br J Pharmacol.	PMID:21133893 |

	SÃ¡nchez R, Moreno-Baylach MJ, GarcÃa-Horsman JA, MÃ¤nnistÃ¶ PT.	2011	PMCID:PMC3166694
The ECM protein LTBP-2 is a suppressor of esophageal squamous cell carcinoma tumor formation but higher tumor expression associates with poor patient outcome.	Chan SH, Yee Ko JM, Chan KW, Chan YP, Tao Q, Hyytiainen M, Keski-Oja J, Law S, Srivastava G, Tang J, Tsao SW, Chen H, Stanbridge EJ, Lung ML.	Int J Cancer. 2011	PMID:20878956
Tumor-derived endothelial cells evade apoptotic activity of the interferon-inducible IFI16 gene.	Gugliesi F, Dell'oste V, De Andrea M, Baggetta R, Mondini M, Zannetti C, Bussolati B, Camussi G, Gariglio M, Landolfo S.	J Interferon Cytokine Res. 2011	PMID:21488755
The cytochrome P450 4A/F-20-hydroxyeicosatetraenoic acid system: a regulator of endothelial precursor cells derived from human umbilical cord blood.	Guo AM, Janic B, Sheng J, Falck JR, Roman RJ, Edwards PA, Arbab AS, Scicli AG.	J Pharmacol Exp Ther. 2011	PMID:21527533 | PMCID:PMC3141901
Tongxinluo promotes mesenchymal stem cell tube formation in vitro.	Hu XY, Wang WX, Yu MJ, Liu XB, Wu RR, Gao F, Huang X, Cao J, Xie XJ, Wang JA.	J Zhejiang Univ Sci B. 2011	PMID:21796805 | PMCID:PMC3150718
Immortalization of swine umbilical vein endothelial cells (SUVECs) with the simian virus 40 large-T antigen.	Chrusciel M, Bodek G, Kirtiklis L, Lewczuk B, Hyder CL, Blitek A, Kaczmarek MM, Ziecik AJ, Andronowska A.	Mol Reprod Dev. 2011	PMID:21786362
Autocrine activity of soluble Flt-1 controls endothelial cell function and angiogenesis.	Ahmad S, Hewett PW, Al-Ani B, Sissaoui S, Fujisawa T, Cudmore MJ, Ahmed A.	Vasc Cell. 2011	PMID:21752276 | PMCID:PMC3173355
CD36-mediated activation of endothelial cell apoptosis by an N-terminal recombinant fragment of thrombospondin-2 inhibits breast cancer growth and metastasis in vivo.	Koch M, Hussein F, Woeste A, GrÃ¼ndker C, Frontzek K, Emons G, Hawighorst T.	Breast Cancer Res Treat. 2011	PMID:20714802 | PMCID:PMC3291836
Repair of senescent myocardium by mesenchymal stem cells is dependent on the age of donor mice.	Khan M, Mohsin S, Khan SN, Riazuddin S.	J Cell Mol Med. 2011	PMID:20041970
The inhibition of angiogenesis and tumor growth by denbinobin is associated with the blocking of insulin-like growth factor-1 receptor signaling.	Tsai AC, Pan SL, Lai CY, Wang CY, Chen CC, Shen CC, Teng CM.	J Nutr Biochem. 2011	PMID:20951021
Ds-echinoside A, a new triterpene glycoside derived from sea cucumber, exhibits antimetastatic activity via the inhibition of NF-Î°B-dependent MMP-9 and VEGF expressions.	Zhao Q, Liu ZD, Xue Y, Wang JF, Li H, Tang QJ, Wang YM, Dong P, Xue CH.	J Zhejiang Univ Sci B. 2011	PMID:21726060 | PMCID:PMC3134607
Regulation of vascular endothelial growth factor signaling by miR-200b.	Choi YC, Yoon S, Jeong Y, Yoon J, Baek K.	Mol Cells. 2011	PMID:21544626
Nitric oxide synthase gene transfer restores activity of circulating angiogenic cells from patients with coronary artery disease.	Ward MR, Thompson KA, Isaac K, Vecchiarelli J, Zhang Q, Stewart DJ, Kutryk MJ.	Mol Ther. 2011	PMID:21522135 | PMCID:PMC3129566
Dual targeting of Bcl-2 and VEGF: a potential strategy to improve therapy for prostate cancer.	Anai S, Sakamoto N, Sakai Y, Tanaka M, Porvasnik S, Urbanek C, Cao W, Goodison S, Rosser CJ.	Urol Oncol. 2011	PMID:19576799
EGFL6 promotes endothelial cell migration and angiogenesis through the activation of extracellular signal-regulated kinase.	Chim SM, Qin A, Tickner J, Pavlos N, Davey T, Wang H, Guo Y, Zheng MH, Xu J.	J Biol Chem. 2011	PMID:21531721 | PMCID:PMC3121348
Alginate hydrogels for three-dimensional organ culture of ovaries and oviducts.	King SM, Quartuccio S, Hilliard TS, Inoue K, Burdette JE.	J Vis Exp. 2011	PMID:21712801
Nitric oxide counters the inhibitory effects of uremic toxin indoxyl sulfate on endothelial cells by governing ERK MAP kinase and myosin light chain activation.	Kharait S, Haddad DJ, Springer ML.	Biochem Biophys Res Commun. 2011	PMID:21621512 | PMCID:PMC3121259
In vitro anti-angiogenic properties of LGD1069, a selective retinoid X-receptor agonist through down-regulating Runx2 expression on Human endothelial cells.	Fu J, Wang W, Liu YH, Lu H, Luo Y.	BMC Cancer. 2011	PMID:21649908 | PMCID:PMC3120806
Lysophosphatidic acid suppresses endothelial cell CD36 expression and promotes angiogenesis via a PKD-1-dependent signaling pathway.	Ren B, Hale J, Srikanthan S, Silverstein RL.	Blood. 2011	PMID:21441463 | PMCID:PMC3112047
AMP-activated protein kinase rescues the angiogenic functions of endothelial progenitor cells via manganese superoxide dismutase induction in type 1 diabetes.	Wang XR, Zhang MW, Chen DD, Zhang Y, Chen AF.	Am J Physiol Endocrinol Metab. 2011	PMID:21427411 | PMCID:PMC3118597
Rottlerin exhibits antiangiogenic effects in vitro.	Valacchi G, Pecorelli A, Sticozzi C, Torricelli C, Muscettola M, Aldinucci C, Maioli E.	Chem Biol Drug Des. 2011	PMID:21435184
Trimodal glioblastoma treatment consisting of concurrent radiotherapy, temozolomide, and the novel TGF-Î² receptor I kinase inhibitor LY2109761.	Zhang M, Herion TW, Timke C, Han N, Hauser K, Weber KJ, Peschke P, Wirkner U, Lahn M, Huber PE.	Neoplasia. 2011	PMID:21677877 | PMCID:PMC3114247
(-)-Epigallocatechin-3-gallate inhibits VEGF expression induced by IL-6 via Stat3 in gastric cancer.	Zhu BH, Chen HY, Zhan WH, Wang CY, Cai SR, Wang Z, Zhang CH, He YL.	World J Gastroenterol. 2011	PMID:21633597 | PMCID:PMC3098399
The integrin coactivator kindlin-2 plays a critical role in angiogenesis in mice and zebrafish.	Pluskota E, Dowling JJ, Gordon N, Golden JA, Szpak D, West XZ, Nestor C, Ma YQ, Bialkowska K, Byzova T, Plow EF.	Blood. 2011	PMID:21378273 | PMCID:PMC3100704
A deficiency of uPAR alters endothelial angiogenic function and cell morphology.	Balsara RD, Merryman R, Virjee F, Northway C, Castellino FJ, Ploplis VA.	Vasc Cell. 2011	PMID:21535874 | PMCID:PMC3105951

The natural compound n-butylidenephthalide derived from the volatile oil of Radix Angelica sinensis inhibits angiogenesis in vitro and in vivo.	Yeh JC, Cindrova-Davies T, Belleri M, Morbidelli L, Miller N, Cho CW, Chan K, Wang YT, Luo GA, Ziche M, Presta M, Charnock-Jones DS, Fan TP.	Angiogenesis. 2011	PMID:21327473
Endothelial Grb2-associated binder 1 is crucial for postnatal angiogenesis.	Zhao J, Wang W, Ha CH, Kim JY, Wong C, Redmond EM, Hamik A, Jain MK, Feng GS, Jin ZG.	Arterioscler Thromb Vasc Biol. 2011	PMID:21372298 | PMCID:PMC3094153
Lactoferrin inhibits the inflammatory and angiogenic activation of bovine aortic endothelial cells.	Yeom M, Park J, Lee B, Choi SY, Kim KS, Lee H, Hahm DH.	Inflamm Res. 2011	PMID:21161563
Detection of prostate-specific membrane antigen on HUVECs in response to breast tumor-conditioned medium.	Liu T, Jabbes M, Nedrow-Byers JR, Wu LY, Bryan JN, Berkman CE.	Int J Oncol. 2011	PMID:21331445
Indirubin-3'-monoxime, a derivative of a chinese antileukemia medicine, inhibits angiogenesis.	Kim JK, Shin EK, Kang YH, Park JH.	J Cell Biochem. 2011	PMID:21337385
Pitavastatin-induced angiogenesis and arteriogenesis is mediated by Notch1 in a murine hindlimb ischemia model without induction of VEGF.	Kikuchi R, Takeshita K, Uchida Y, Kondo M, Cheng XW, Nakayama T, Yamamoto K, Matsushita T, Liao JK, Murohara T.	Lab Invest. 2011	PMID:21301413
A YKL-40-neutralizing antibody blocks tumor angiogenesis and progression: a potential therapeutic agent in cancers.	Faibish M, Francescone R, Bentley B, Yan W, Shao R.	Mol Cancer Ther. 2011	PMID:21357475 | PMCID:PMC3091949
[The effects of stem cell factor on proliferation, transmigration, capillary tube formation of endothelial cells and on the chemotaxis of CD133(+) cells].	Yan X, Liu B, Lu SH, Ge ML, Li XX, Zheng YZ.	Zhonghua Xue Ye Xue Za Zhi. 2011	PMID:21729602
Ethyl pyruvate, an anti-inflammatory agent, inhibits tumor angiogenesis through inhibition of the NF-ÎºB signaling pathway.	Park SY, Yi EY, Jung M, Lee YM, Kim YJ.	Cancer Lett. 2011	PMID:21333439
Vascular endothelial growth factor receptor-2 couples cyclo-oxygenase-2 with pro-angiogenic actions of leptin on human endothelial cells.	Garonna E, Botham KM, Birdsey GM, Randi AM, Gonzalez-Perez RR, Wheeler-Jones CP.	PLoS One. 2011	PMID:21533119 | PMCID:PMC3078934
Insulin-like growth factor binding protein-related protein 1 mediates VEGF-induced proliferation, migration and tube formation of retinal endothelial cells.	Sun T, Cao H, Xu L, Zhu B, Gu Q, Xu X.	Curr Eye Res. 2011	PMID:21405955
Requirement of the nuclear localization of transcription enhancer factor 3 for proliferation, migration, tube formation, and angiogenesis induced by vascular endothelial growth factor.	Liu X, Zhao D, James L, Li J, Zeng H.	FASEB J. 2011	PMID:21169383
Stimulation of tube formation mediated through the prostaglandin EP2 receptor in rat luteal endothelial cells.	Sakurai T, Suzuki K, Yoshie M, Hashimoto K, Tachikawa E, Tamura K.	J Endocrinol. 2011	PMID:21273371
Role of axonal guidance factor netrin-1 in human placental vascular growth.	Wang Q, Zhu J, Zou L, Yang Y.	J Huazhong Univ Sci Technolog Med Sci. 2011	PMID:21505994
[Effect of RNA interference targeting HIF-1Î± gene on biological behavior of human esophageal squamous cell carcinoma and gastric adenocarcinoma cells in vitro].	Zeng KF, Jin HL, Zhang WF, Xiao B, Zhu H, Hao B, Shi RH.	Zhonghua Zhong Liu Za Zhi. 2011	PMID:21575495
SR16388: a steroidal antiangiogenic agent with potent inhibitory effect on tumor growth in vivo.	Chao WR, Amin K, Shi Y, Hobbs P, Tanabe M, Tanga M, Jong L, Collins N, Peters R, Laderoute K, Dinh D, Yean D, Hou C, Sato B, Alt C, Sambucetti L.	Angiogenesis. 2011	PMID:21104121
CXCL8 enhances the angiogenic activity of umbilical cord blood-derived outgrowth endothelial cells in vitro.	Kimura T, Kohno H, Matsuoka Y, Murakami M, Nakatsuka R, Hase M, Yasuda K, Uemura Y, Sasaki Y, Fukuhara S, Sonoda Y.	Cell Biol Int. 2011	PMID:20958269
Title	Description	ShortDetails	Identifiers
Suppression of NHE1 by small interfering RNA inhibits HIF-1Î±-induced angiogenesis in vitro via modulation of calpain activity.	Mo XG, Chen QW, Li XS, Zheng MM, Ke DZ, Deng W, Li GQ, Jiang J, Wu ZQ, Wang L, Wang P, Yang Y, Cao GY.	Microvasc Res. 2011	PMID:21185840
Discovery of a pyrazole derivative promoting angiogenesis through modulating reactive oxygen species and interferon-inducible protein 10 levels.	Wang M, Zhang J, Wu X, Jin X, Zhao B, Zhang L, Yuan H, Zhou H, Gao B, Lv W, Kong X, Miao J.	Mol Biol Rep. 2011	PMID:20842451
Galbanic acid isolated from Ferula assafoetida exerts in vivo anti-tumor activity in association with anti-angiogenesis and anti-proliferation.	Kim KH, Lee HJ, Jeong SJ, Lee HJ, Lee EO, Kim HS, Zhang Y, Ryu SY, Lee MH, LÃ¼ J, Kim SH.	Pharm Res. 2011	PMID:21063754
Far-infrared radiation promotes angiogenesis in human microvascular endothelial cells via extracellular signal-regulated kinase activation.	Rau CS, Yang JC, Jeng SF, Chen YC, Lin CJ, Wu CJ, Lu TH, Hsieh CH.	Photochem Photobiol. 2011	PMID:21143604
Glioma stem/progenitor cells contribute to neovascularization via transdifferentiation.	Dong J, Zhao Y, Huang Q, Fei X, Diao Y, Shen Y, Xiao H, Zhang T, Lan Q, Gu X.	Stem Cell Rev. 2011	PMID:20697979
Prognostic significance of BRMS1 expression in human melanoma and its role in tumor angiogenesis.	Li J, Cheng Y, Tai D, Martinka M, Welch DR, Li G.	Oncogene. 2011	PMID:20935672 | PMCID:PMC3235331

Grb-2-associated binder 1 (Gab1) regulates postnatal ischemic and VEGF-induced angiogenesis through the protein kinase A-endothelial NOS pathway.	Lu Y, Xiong Y, Huo Y, Han J, Yang X, Zhang R, Zhu DS, Klein-Hessling S, Li J, Zhang X, Han X, Li Y, Shen B, He Y, Shibuya M, Feng GS, Luo J.	Proc Natl Acad Sci U S A. 2011	PMID:21282639 | PMCID:PMC3041066
Targeted delivery of antisense inhibitor of miRNA for antiangiogenesis therapy using cRGD-functionalized nanoparticles.	Liu XQ, Song WJ, Sun TM, Zhang PZ, Wang J.	Mol Pharm. 2011	PMID:21138272
Complementary effects of extracellular nucleotides and platelet-derived extracts on angiogenesis of vasa vasorum endothelial cells in vitro and subcutaneous Matrigel plugs in vivo.	Roedersheimer M, Nijmeh H, Burns N, Sidiakova AA, Stenmark KR, Gerasimovskaya EV.	Vasc Cell. 2011	PMID:21349161 | PMCID:PMC3045351
N-benzyl-5-phenyl-1H-pyrazole-3-carboxamide promotes vascular endothelial cell angiogenesis and migration in the absence of serum and FGF-2.	Zhang HY, Su L, Huang B, Zhao J, Zhao BX, Zhang SL, Miao JY.	Acta Pharmacol Sin. 2011	PMID:21293473
LMWH bemiparin and ULMWH RO-14 reduce the endothelial angiogenic features elicited by leukemia, lung cancer, or breast cancer cells.	Vignoli A, Marchetti M, Russo L, Cantalino E, Diani E, Bonacina G, Falanga A.	Cancer Invest. 2011	PMID:21261475
[Experimental study on culture method of human umbilical vein endothelial cells].	Cai W, Liang L, Ji P, Zhang W, Zhang Z, Zhang Y, Xiao X, Bai X, Zhu H, Hu D, Han H.	Zhongguo Xiu Fu Chong Jian Wai Ke Za Zhi. 2011	PMID:21427839
miR-200b targets Ets-1 and is down-regulated by hypoxia to induce angiogenic response of endothelial cells.	Chan YC, Khanna S, Roy S, Sen CK.	J Biol Chem. 2011	PMID:21081489 | PMCID:PMC3023502
Angiogenic properties of aged adipose derived mesenchymal stem cells after hypoxic conditioning.	Efimenko A, Starostina E, Kalinina N, Stolzing A.	J Transl Med. 2011	PMID:21244679 | PMCID:PMC3033332
Novel glycosaminoglycan biosynthetic inhibitors affect tumor-associated angiogenesis.	Raman K, Ninomiya M, Nguyen TK, Tsuzuki Y, Koketsu M, Kuberan B.	Biochem Biophys Res Commun. 2011	PMID:21094131 | PMCID:PMC3031167
The anti-inflammatory cytokine interleukin 19 is expressed by and angiogenic for human endothelial cells.	Jain S, Gabunia K, Kelemen SE, Panetti TS, Autieri MV.	Arterioscler Thromb Vasc Biol. 2011	PMID:20966397 | PMCID:PMC3005139
SKLB610: a novel potential inhibitor of vascular endothelial growth factor receptor tyrosine kinases inhibits angiogenesis and tumor growth in vivo.	Cao ZX, Zheng RL, Lin HJ, Luo SD, Zhou Y, Xu YZ, Zeng XX, Wang Z, Zhou LN, Mao YQ, Yang L, Wei YQ, Yu LT, Yang SY, Zhao YL.	Cell Physiol Biochem. 2011	PMID:21691074
Functional endothelial cells derived from embryonic stem cells labeled with HIV transactivator peptide-conjugated superparamagnetic nanoparticles.	Gao B, Fu WG, Dong ZH, Fang ZD, Liu ZJ, Si Y, Zhang XM, Wang YQ.	Chin Med J (Engl). 2011	PMID:21362384
Angiogenesis: the role of PDGF-BB on adipose-tissue derived stem cells (ASCs).	Gehmert S, Gehmert S, Hidayat M, Sultan M, Berner A, Klein S, Zellner J, MÃ¼ller M, Prantl L.	Clin Hemorheol Microcirc. 2011	PMID:21876230
CD44 mediates oligosaccharides of hyaluronan-induced proliferation, tube formation and signal transduction in endothelial cells.	Wang YZ, Cao ML, Liu YW, He YQ, Yang CX, Gao F.	Exp Biol Med (Maywood). 2011	PMID:21239738
Modulation of endothelial cell migration and angiogenesis: a novel function for the tandem-repeat" lectin galectin-8."	Delgado VM, Nugnes LG, Colombo LL, Troncoso MF, FernÃ¡ndez MM, Malchiodi EL, Frahm I, Croci DO, Compagno D, Rabinovich GA, Wolfenstein-Todel C, Elola MT.	FASEB J. 2011	PMID:20876211
CTGF expression is up-regulated by PROK1 in early pregnancy and influences HTR-8/Svneo cell adhesion and network formation.	Waddell JM, Evans J, Jabbour HN, Denison FC.	Hum Reprod. 2011	PMID:21098624 | PMCID:PMC3005999
Berberine inhibits angiogenic potential of Hep G2 cell line through VEGF down-regulation in vitro.	Jie S, Li H, Tian Y, Guo D, Zhu J, Gao S, Jiang L.	J Gastroenterol Hepatol. 2011	PMID:21175812
Coenzyme Q10 decreases basic fibroblast growth factor (bFGF)-induced angiogenesis by blocking ERK activation.	Choi JS, Park SY, Yi EY, Kim YJ, Jeong JW.	Oncol Res. 2011	PMID:22715588
Synergistic inhibition of endothelial cell proliferation, tube formation, and sprouting by cyclosporin A and itraconazole.	Nacev BA, Liu JO.	PLoS One. 2011	PMID:21969860 | PMCID:PMC3182171
Heterogeneity in SDF-1 expression defines the vasculogenic potential of adult cardiac progenitor cells.	Rodrigues CO, Shehadeh LA, Hoosien M, Otero V, Chopra I, Tsinoremas NF, Bishopric NH.	PLoS One. 2011	PMID:21887363 | PMCID:PMC3161114
Toll-like receptor 2 induced angiogenesis and invasion is mediated through the Tie2 signalling pathway in rheumatoid arthritis.	Saber T, Veale DJ, Balogh E, McCormick J, NicAnUltaigh S, Connolly M, Fearon U.	PLoS One. 2011	PMID:21858161 | PMCID:PMC3157402
Anti-angiogenic and anti-inflammatory properties of kahweol, a coffee diterpene.	CÃ¡rdenas C, Quesada AR, Medina MA.	PLoS One. 2011	PMID:21858104 | PMCID:PMC3153489
Asiatic acid inhibits pro-angiogenic effects of VEGF and human gliomas in endothelial cell culture models.	Kavitha CV, Agarwal C, Agarwal R, Deep G.	PLoS One. 2011	PMID:21826202 | PMCID:PMC3149605

Inhibition of APE1/Ref-1 redox activity with APX3330 blocks retinal angiogenesis in vitro and in vivo.	Jiang A, Gao H, Kelley MR, Qiao X.	Vision Res. 2011	PMID:20937296 | PMCID:PMC3010438
Glucocorticoid-mediated inhibition of angiogenic changes in human endothelial cells is not caused by reductions in cell proliferation or migration.	Logie JJ, Ali S, Marshall KM, Heck MM, Walker BR, Hadoke PW.	PLoS One. 2010	PMID:21217824 | PMCID:PMC3013101
Effect of nitroxoline on angiogenesis and growth of human bladder cancer.	Shim JS, Matsui Y, Bhat S, Nacev BA, Xu J, Bhang HE, Dhara S, Han KC, Chong CR, Pomper MG, So A, Liu JO.	J Natl Cancer Inst. 2010	PMID:21088277 | PMCID:PMC3001967
Elevated AKR1C3 expression promotes prostate cancer cell survival and prostate cell-mediated endothelial cell tube formation: implications for prostate cancer progression.	Dozmorov MG, Azzarello JT, Wren JD, Fung KM, Yang Q, Davis JS, Hurst RE, Culkin DJ, Penning TM, Lin HK.	BMC Cancer. 2010	PMID:21134280 | PMCID:PMC3013086
Angiogenic role of orexin-A via the activation of extracellular signal-regulated kinase in endothelial cells.	Kim MK, Park HJ, Kim SR, Choi YK, Shin HK, Jeon JH, Jang HO, Yun I, Bae SK, Bae MK.	Biochem Biophys Res Commun. 2010	PMID:21040705
Interleukin 18 induces angiogenesis in vitro and in vivo via Src and Jnk kinases.	Amin MA, Rabquer BJ, Mansfield PJ, Ruth JH, Marotte H, Haas CS, Reamer EN, Koch AE.	Ann Rheum Dis. 2010	PMID:20679476
Metformin treatment may increase omentin-1 levels in women with polycystic ovary syndrome.	Tan BK, Adya R, Farhatullah S, Chen J, Lehnert H, Randeva HS.	Diabetes. 2010	PMID:20852028 | PMCID:PMC2992762
Kisspeptin-10 inhibits angiogenesis in human placental vessels ex vivo and endothelial cells in vitro.	Ramaesh T, Logie JJ, Roseweir AK, Millar RP, Walker BR, Hadoke PW, Reynolds RM.	Endocrinology. 2010	PMID:20926586
Enhancement of angiogenic potential of endothelial cells by contact with retinal pigment epithelial cells in a model simulating pathological conditions.	Dardik R, Livnat T, Nisgav Y, Weinberger D.	Invest Ophthalmol Vis Sci. 2010	PMID:20702828
Antiangiogenic and antimitotic effects of aspirin in hypoxiaâ€"reoxygenation modulation of the LOX-1-NADPH oxidase axis as a potential mechanism.	Khaidakov M, Szwedo J, Mitra S, Ayyadevara S, Dobretsov M, Lu J, Mehta JL.	J Cardiovasc Pharmacol. 2010	PMID:20881612
The indazole derivative YD-3 specifically inhibits thrombin-induced angiogenesis in vitro and in vivo.	Peng CY, Pan SL, Pai HC, Tsai AC, Guh JH, Chang YL, Kuo SC, Lee FY, Teng CM.	Shock. 2010	PMID:20351626
The neuropeptide catestatin acts as a novel angiogenic cytokine via a basic fibroblast growth factor-dependent mechanism.	Theurl M, Schgoer W, Albrecht K, Jeschke J, Egger M, Beer AG, Vasiljevic D, Rong S, Wolf AM, Bahlmann FH, Patsch JR, Wolf D, Schratzberger P, Mahata SK, Kirchmair R.	Circ Res. 2010	PMID:20930149
Adipose tissue-derived stem cells secrete CXCL5 cytokine with chemoattractant and angiogenic properties.	Zhang H, Ning H, Banie L, Wang G, Lin G, Lue TF, Lin CS.	Biochem Biophys Res Commun. 2010	PMID:21034724 | PMCID:PMC3530412
Deguelin--an inhibitor to tumor lymphangiogenesis and lymphatic metastasis by downregulation of vascular endothelial cell growth factor-D in lung tumor model.	Hu J, Ye H, Fu A, Chen X, Wang Y, Chen X, Ye X, Xiao W, Duan X, Wei Y, Chen L.	Int J Cancer. 2010	PMID:20162567
Nobiletin, a citrus polymethoxyflavonoid, suppresses multiple angiogenesis-related endothelial cell functions and angiogenesis in vivo.	Kunimasa K, Ikekita M, Sato M, Ohta T, Yamori Y, Ikeda M, Kuranuki S, Oikawa T.	Cancer Sci. 2010	PMID:20670297
Rac1-dependent intracellular superoxide formation mediates vascular endothelial growth factor-induced placental angiogenesis in vitro.	Li SM, Zeng LW, Feng L, Chen DB.	Endocrinology. 2010	PMID:20844008 | PMCID:PMC2954717
Reversible transdifferentiation of blood vascular endothelial cells to a lymphatic-like phenotype in vitro.	Cooley LS, Handsley MM, Zhou Z, Lafleur MA, Pennington CJ, Thompson EW, PÃ¶schl E, Edwards DR.	J Cell Sci. 2010	PMID:20940254
Differential effects of sulfated triterpene glycosides, holothurin A1, and 24-dehydroechinoside A, on antimetastasic activity via regulation of the MMP-9 signal pathway.	Zhao Q, Xue Y, Liu ZD, Li H, Wang JF, Li ZJ, Wang YM, Dong P, Xue CH.	J Food Sci. 2010	PMID:21535601
WSS25 inhibits growth of xenografted hepatocellular cancer cells in nude mice by disrupting angiogenesis via blocking bone morphogenetic protein (BMP)/Smad/Id1 signaling.	Qiu H, Yang B, Pei ZC, Zhang Z, Ding K.	J Biol Chem. 2010	PMID:20679340 | PMCID:PMC2952266
A novel antiangiogenic peptide derived from hepatocyte growth factor inhibits neovascularization in vitro and in vivo.	Xu Y, Zhao H, Zheng Y, Gu Q, Ma J, Xu X.	Mol Vis. 2010	PMID:21031024 | PMCID:PMC2956696
MRP4 knockdown enhances migration, suppresses apoptosis, and produces aggregated morphology in human retinal vascular endothelial cells.	Tagami M, Kusuhara S, Imai H, Uemura A, Honda S, Tsukahara Y, Negi A.	Biochem Biophys Res Commun. 2010	PMID:20804728
Title	Description	ShortDetails	Identifiers
The soluble fragment of VE-cadherin inhibits angiogenesis by reducing endothelial cell proliferation and tube capillary formation.	Li H, Shi X, Liu J, Hu C, Zhang X, Liu H, Jin J, Opolon P, Vannier JP, Perricaudet M, Janin A, Soria C, Lu H.	Cancer Gene Ther. 2010	PMID:20559333
Evaluation of endothelial cell culture as a model system of vascular ageing.	Boisen L, Drasbek KR, Pedersen AS, Kristensen P.	Exp Gerontol. 2010	PMID:20600781
Effects of transfection of ICAP-1Î± and its mutants on adhesion and migration of 2H-11 cells.	Zhang J, Luo W, Liu Z, Lin J, Cheng Z.	J Huazhong Univ Sci Technolog Med	PMID:21063836

		Sci. 2010	
Role of nitric oxide signaling in endothelial differentiation of embryonic stem cells.	Huang NF, Fleissner F, Sun J, Cooke JP.	Stem Cells Dev. 2010	PMID:20064011 | PMCID:PMC3121801
[Inhibitory effect of ginsenoside Rg3 on the tube-like structure formation in human nasopharyngeal carcinoma HNE-1 cell line in vitro].	Wang HB, Lin YC, Zeng D, Lin W, Hong CQ, Lin WZ, Chen JY.	Zhonghua Zhong Liu Za Zhi. 2010	PMID:21163062
Dynamics of bone marrow-derived endothelial progenitor cell/mesenchymal stem cell interaction in co-culture and its implications in angiogenesis.	Aguirre A, Planell JA, Engel E.	Biochem Biophys Res Commun. 2010	PMID:20732306
Scutellarin promotes in vitro angiogenesis in human umbilical vein endothelial cells.	Gao ZX, Huang DY, Li HX, Zhang LN, Lv YH, Cui HD, Zheng JH.	Biochem Biophys Res Commun. 2010	PMID:20709020
Influence of non-toxic doses of bevacizumab and ranibizumab on endothelial functions and inhibition of angiogenesis.	Barzelay A, Lowenstein A, George J, Barak A.	Curr Eye Res. 2010	PMID:20795866
MMP-2 alters VEGF expression via alphaVbeta3 integrin-mediated PI3K/AKT signaling in A549 lung cancer cells.	Chetty C, Lakka SS, Bhoopathi P, Rao JS.	Int J Cancer. 2010	PMID:20027628 | PMCID:PMC2891576
Expression profiling of ETS and MMP factors in VEGF-activated endothelial cells: role of MMP-10 in VEGF-induced angiogenesis.	Heo SH, Choi YJ, Ryoo HM, Cho JY.	J Cell Physiol. 2010	PMID:20432469
Sonic hedgehog induces angiogenesis via Rho kinase-dependent signaling in endothelial cells.	Renault MA, Roncalli J, Tongers J, Thorne T, Klyachko E, Misener S, Volpert OV, Mehta S, Burg A, Luedemann C, Qin G, Kishore R, Losordo DW.	J Mol Cell Cardiol. 2010	PMID:20478312 | PMCID:PMC2917529
Comparative study on circulating endothelial progenitor cells in systemic lupus erythematosus patients at active stage.	Deng XL, Li XX, Liu XY, Sun L, Liu R.	Rheumatol Int. 2010	PMID:19847436
Anti-angiogenic activity of methanol extract of Phellinus linteus and its fractions.	Lee YS, Kim YH, Shin EK, Kim DH, Lim SS, Lee JY, Kim JK.	J Ethnopharmacol. 2010	PMID:20554007
alpha6-integrin subunit plays a major role in the proangiogenic properties of endothelial progenitor cells.	Bouvard C, Gafsou B, Dizier B, Galy-Fauroux I, Lokajczyk A, Boisson-Vidal C, Fischer AM, Helley D.	Arterioscler Thromb Vasc Biol. 2010	PMID:20508204
Modulation of the angiogenic phenotype of normal and systemic sclerosis endothelial cells by gain-loss of function of pentraxin 3 and matrix metalloproteinase 12.	Margheri F, SerratÃ¬ S, Lapucci A, ChillÃ A, Bazzichi L, Bombardieri S, Kahaleh B, Calorini L, Bianchini F, Fibbi G, Del Rosso M.	Arthritis Rheum. 2010	PMID:20506099
Mechanical stimulation of the pro-angiogenic capacity of human fracture haematoma: involvement of VEGF mechano-regulation.	Groothuis A, Duda GN, Wilson CJ, Thompson MS, Hunter MR, Simon P, Bail HJ, van Scherpenzeel KM, Kasper G.	Bone. 2010	PMID:20580871
Sphingosine kinase 1 is critically involved in nitric oxide-mediated human endothelial cell migration and tube formation.	Schwalm S, Pfeilschifter J, Huwiler A.	Br J Pharmacol. 2010	PMID:20649568 | PMCID:PMC2936837
Antiangiogenetic effects of 4 varieties of grapes in vitro.	Liu M, Liu RH, Song BB, Li CF, Lin LQ, Zhang CP, Zhao JL, Liu JR.	J Food Sci. 2010	PMID:20722961
Junctional adhesion molecule-C is a soluble mediator of angiogenesis.	Rabquer BJ, Amin MA, Teegala N, Shaheen MK, Tsou PS, Ruth JH, Lesch CA, Imhof BA, Koch AE.	J Immunol. 2010	PMID:20592283 | PMCID:PMC3003428
Identification of a metalloprotease-chemokine signaling system in the ovarian cancer microenvironment: implications for antiangiogenic therapy.	Agarwal A, Tressel SL, Kaimal R, Balla M, Lam FH, Covic L, Kuliopulos A.	Cancer Res. 2010	PMID:20570895 | PMCID:PMC2917243
Activation of the ERK signaling pathway is involved in CD151-induced angiogenic effects on the formation of CD151-integrin complexes.	Zuo HJ, Lin JY, Liu ZY, Liu WF, Liu T, Yang J, Liu Y, Wang DW, Liu ZX.	Acta Pharmacol Sin. 2010	PMID:20581856
Comparative proteomics profiling reveals role of smooth muscle progenitors in extracellular matrix production.	Simper D, Mayr U, Urbich C, Zampetaki A, Prokopi M, Didangelos A, Saje A, Mueller M, Benbow U, Newby AC, Apweiler R, Rahman S, Dimmeler S, Xu Q, Mayr M.	Arterioscler Thromb Vasc Biol. 2010	PMID:20431068
Extracellular protease ADAMTS9 suppresses esophageal and nasopharyngeal carcinoma tumor formation by inhibiting angiogenesis.	Lo PH, Lung HL, Cheung AK, Apte SS, Chan KW, Kwong FM, Ko JM, Cheng Y, Law S, Srivastava G, Zabarovsky ER, Tsao SW, Tang JC, Stanbridge EJ, Lung ML.	Cancer Res. 2010	PMID:20551050 | PMCID:PMC2896444
Hyperbaric oxygen induces a cytoprotective and angiogenic response in human microvascular endothelial cells.	Godman CA, Chheda KP, Hightower LE, Perdrizet G, Shin DG, Giardina C.	Cell Stress Chaperones. 2010	PMID:19949909 | PMCID:PMC3082642
Mechanisms of angiogenesis: role of hydrogen sulphide.	Wang MJ, Cai WJ, Zhu YC.	Clin Exp Pharmacol Physiol. 2010	PMID:20148917
Effects on in vitro and in vivo angiogenesis induced by small peptides carrying adhesion	Conconi MT, Ghezzo F, Dettin M, Urbani L, Grandi C, Guidolin D, Nico B, Di Bello C, Ribatti D, Parnigotto PP.	J Pept Sci. 2010	PMID:20552562

sequences.			
[Effect of soluble epoxide hydrolase inhibitor on the function of endothelial progenitor cells in patients with coronary heart disease].	Xu D, Chen C, Jiang Y, Zhao S, Liu Z, Xie X, Liu L.	Zhong Nan Da Xue Xue Bao Yi Xue Ban. 2010	PMID:20693709
The integrin co-activator Kindlin-3 is expressed and functional in a non-hematopoietic cell, the endothelial cell.	Bialkowska K, Ma YQ, Bledzka K, Sossey-Alaoui K, Izem L, Zhang X, Malinin N, Qin J, Byzova T, Plow EF.	J Biol Chem. 2010	PMID:20378539 | PMCID:PMC2881789
Bee venom inhibits tumor angiogenesis and metastasis by inhibiting tyrosine phosphorylation of VEGFR-2 in LLC-tumor-bearing mice.	Huh JE, Baek YH, Lee MH, Choi DY, Park DS, Lee JD.	Cancer Lett. 2010	PMID:20188461
Adipocyte-derived microvesicles are associated with multiple angiogenic factors and induce angiogenesis in vivo and in vitro.	Aoki N, Yokoyama R, Asai N, Ohki M, Ohki Y, Kusubata K, Heissig B, Hattori K, Nakagawa Y, Matsuda T.	Endocrinology. 2010	PMID:20382694
In vitro evaluation of the bioactive factors preserved in porcine small intestinal submucosa through cellular biological approaches.	Yang B, Zhou L, Sun Z, Yang R, Chen Y, Dai Y.	J Biomed Mater Res A. 2010	PMID:19768788
Angiostatic effects of K252a, a Trk inhibitor, in murine brain capillary endothelial cells.	Lecht S, Arien-Zakay H, Kohan M, Lelkes PI, Lazarovici P.	Mol Cell Biochem. 2010	PMID:20148355
Soluble epithin/PRSS14 secreted from cancer cells contains active angiogenic potential.	Kim SB, Lee D, Jeong JW, Kim C, Park D, Kim MG.	Mol Cells. 2010	PMID:20652801
Anti-angiogenic and cytotoxicity studies of some medicinal plants.	Ng KW, Salhimi SM, Majid AM, Chan KL.	Planta Med. 2010	PMID:20112179
AP-1 (Fra-1/c-Jun)-mediated induction of expression of matrix metalloproteinase-2 is required for 15S-hydroxyeicosatetraenoic acid-induced angiogenesis.	Singh NK, Quyen DV, Kundumani-Sridharan V, Brooks PC, Rao GN.	J Biol Chem. 2010	PMID:20353950 | PMCID:PMC2878006
CYLD regulates angiogenesis by mediating vascular endothelial cell migration.	Gao J, Sun L, Huo L, Liu M, Li D, Zhou J.	Blood. 2010	PMID:20194890
DLC2 modulates angiogenic responses in vascular endothelial cells by regulating cell attachment and migration.	Lin Y, Chen NT, Shih YP, Liao YC, Xue L, Lo SH.	Oncogene. 2010	PMID:20208559 | PMCID:PMC2874629
BMP-9 induces proliferation of multiple types of endothelial cells in vitro and in vivo.	Suzuki Y, Ohga N, Morishita Y, Hida K, Miyazono K, Watabe T.	J Cell Sci. 2010	PMID:20406889
Statins inhibit cyclooxygenase-2 and matrix metalloproteinase-9 in human endothelial cells: anti-angiogenic actions possibly contributing to plaque stability.	Massaro M, Zampolli A, Scoditti E, Carluccio MA, Storelli C, Distante A, De Caterina R.	Cardiovasc Res. 2010	PMID:19946014
Sprouty1 inhibits angiogenesis in association with up-regulation of p21 and p27.	Lee S, Bui Nguyen TM, Kovalenko D, Adhikari N, Grindle S, Polster SP, Friesel R, Ramakrishnan S, Hall JL.	Mol Cell Biochem. 2010	PMID:20054616 | PMCID:PMC3334870
Arsenic promotes angiogenesis in vitro via a heme oxygenase-1-dependent mechanism.	Meng D, Wang X, Chang Q, Hitron A, Zhang Z, Xu M, Chen G, Luo J, Jiang B, Fang J, Shi X.	Toxicol Appl Pharmacol. 2010	PMID:20083128
Inhibition of metastasis, angiogenesis, and tumor growth by Chinese herbal cocktail Tien-Hsien Liquid.	Chia JS, Du JL, Hsu WB, Sun A, Chiang CP, Wang WB.	BMC Cancer. 2010	PMID:20429953 | PMCID:PMC2880989
Activation of AMP-activated protein kinase by vascular endothelial growth factor mediates endothelial angiogenesis independently of nitric-oxide synthase.	Stahmann N, Woods A, Spengler K, Heslegrave A, Bauer R, Krause S, Viollet B, Carling D, Heller R.	J Biol Chem. 2010	PMID:20129920 | PMCID:PMC2856272
PDGF and bFGF modulate tube formation in adipose tissue-derived stem cells.	Keerl S, Gehmert S, Gehmert S, Song YH, Alt E.	Ann Plast Surg. 2010	PMID:20224347
Discovery of a benzoxazine derivative promoting angiogenesis in vitro and in vivo.	Dong Z, Cheng Y, Zhao J, Su L, Zhao B, Zhang Y, Zhang S, Miao J.	J Cell Physiol. 2010	PMID:20049873
Antiangiogenic effect of a selective 5-HT4 receptor agonist.	Nishikawa T, Tsuno NH, Shuno Y, Sasaki K, Hongo K, Okaji Y, Sunami E, Kitayama J, Takahashi K, Nagawa H.	J Surg Res. 2010	PMID:19515383
Isolation and characterization of stem cells derived from human third molar tooth germs of young adults: implications in neo-vascularization, osteo-, adipo- and neurogenesis.	Yalvac ME, Ramazanoglu M, Rizvanov AA, Sahin F, Bayrak OF, Salli U, PalotÃ¡s A, Kose GT.	Pharmacogenomics J. 2010	PMID:19721467
Vascular endothelial growth factor activation of endothelial cells is mediated by early growth response-3.	Suehiro J, Hamakubo T, Kodama T, Aird WC, Minami T.	Blood. 2010	PMID:19965691 | PMCID:PMC2845904
A novel compound, NP-184, inhibits the vascular endothelial growth factor induced angiogenesis.	Lin KT, Lien JC, Chung CH, Kuo SC, Huang TF.	Eur J Pharmacol. 2010	PMID:20067787
IL-17 contributes to angiogenesis in rheumatoid arthritis.	Pickens SR, Volin MV, Mandelin AM 2nd, Kolls JK, Pope RM, Shahrara S.	J Immunol. 2010	PMID:20173024 | PMCID:PMC2857761
Title	Description	ShortDetails	Identifiers
Regulation of vascular endothelial growth factor-induced endothelial cell migration by LIM kinase 1-mediated phosphorylation of annexin 1.	CÃ´tÃ© MC, Lavoie JR, Houle F, Poirier A, Rousseau S, Huot J.	J Biol Chem. 2010	PMID:20061392 | PMCID:PMC2832952

15(S)-hydroxyeicosatetraenoic acid-induced angiogenesis requires Src-mediated Egr-1-dependent rapid induction of FGF-2 expression.	Kundumani-Sridharan V, Niu J, Wang D, Van Quyen D, Zhang Q, Singh NK, Subramani J, Karri S, Rao GN.	Blood. 2010	PMID:20053757 | PMCID:PMC2837334
Tetrahydrohyperforin and octahydrohyperforin are two new potent inhibitors of angiogenesis.	MartÃnez-Poveda B, Verotta L, Bombardelli E, Quesada AR, Medina MA.	PLoS One. 2010	PMID:20224821 | PMCID:PMC2835552
ADAMTS9 is a cell-autonomously acting, anti-angiogenic metalloprotease expressed by microvascular endothelial cells.	Koo BH, Coe DM, Dixon LJ, Somerville RP, Nelson CM, Wang LW, Young ME, Lindner DJ, Apte SS.	Am J Pathol. 2010	PMID:20093484 | PMCID:PMC2832168
Angiogenesis and blood vessel stability in inflammatory arthritis.	Kennedy A, Ng CT, Biniecka M, Saber T, Taylor C, O'Sullivan J, Veale DJ, Fearon U.	Arthritis Rheum. 2010	PMID:20187131
Novel angiogenesis inhibitory activity in cinnamon extract blocks VEGFR2 kinase and downstream signaling.	Lu J, Zhang K, Nam S, Anderson RA, Jove R, Wen W.	Carcinogenesis. 2010	PMID:19969552 | PMCID:PMC3105590
Human embryonic stem cell-derived vascular progenitor cells capable of endothelial and smooth muscle cell function.	Hill KL, Obrtlikova P, Alvarez DF, King JA, Keirstead SA, Allred JR, Kaufman DS.	Exp Hematol. 2010	PMID:20067819 | PMCID:PMC2838385
Angiogenic effects of stromal cell-derived factor-1 (SDF-1/CXCL12) variants in vitro and the in vivo expressions of CXCL12 variants and CXCR4 in human critical leg ischemia.	Ho TK, Tsui J, Xu S, Leoni P, Abraham DJ, Baker DM.	J Vasc Surg. 2010	PMID:20206813
Increased angiogenesis induced by chronic lymphocytic leukemia B cells is mediated by leukemia-derived Ang2 and VEGF.	Maffei R, Martinelli S, Castelli I, Santachiara R, Zucchini P, Fontana M, Fiorcari S, Bonacorsi G, Ilariucci F, Torelli G, Marasca R.	Leuk Res. 2010	PMID:19616847
Decidual NK cell-derived conditioned medium enhances capillary tube and network organization in an extravillous cytotrophoblast cell line.	Hu Y, Eastabrook G, Tan R, MacCalman CD, Dutz JP, von Dadelszen P.	Placenta. 2010	PMID:20080299
Targeting retinal and choroid neovascularization using the small molecule inhibitor carboxyamidotriazole.	Afzal A, Caballero S, Palii SS, Jurczyk S, Pardue M, Geroski D, Edelhauser H, Hochhaus G, Kim M, Franklin A, Shapiro G, Grant MB.	Brain Res Bull. 2010	PMID:19679174 | PMCID:PMC3635673
Small RNA interference-mediated gene silencing of heparanase abolishes the invasion, metastasis and angiogenesis of gastric cancer cells.	Zheng L, Jiang G, Mei H, Pu J, Dong J, Hou X, Tong Q.	BMC Cancer. 2010	PMID:20137078 | PMCID:PMC2834619
Anti-neuropilin-1 peptide inhibition of synoviocyte survival, angiogenesis, and experimental arthritis.	Kong JS, Yoo SA, Kim JW, Yang SP, Chae CB, Tarallo V, De Falco S, Ryu SH, Cho CS, Kim WU.	Arthritis Rheum. 2010	PMID:20039409
Evidence that tumor necrosis factor-related apoptosis inducing ligand (TRAIL) inhibits angiogenesis by inducing vascular endothelial cell apoptosis.	Chen PL, Easton AS.	Biochem Biophys Res Commun. 2010	PMID:19962958
DGDA, a local sequence of the kringle 2 domain, is a functional motif of the tissue-type plasminogen activator's antiangiogenic kringle domain.	Kim HK, Joe YA.	Biochem Biophys Res Commun. 2010	PMID:19903452
Compound K inhibits basic fibroblast growth factor-induced angiogenesis via regulation of p38 mitogen activated protein kinase and AKT in human umbilical vein endothelial cells.	Jeong A, Lee HJ, Jeong SJ, Lee HJ, Lee EO, Bae H, Kim SH.	Biol Pharm Bull. 2010	PMID:20522957
Modified C-reactive protein is expressed by stroke neovessels and is a potent activator of angiogenesis in vitro.	Slevin M, Matou-Nasri S, Turu M, Luque A, Rovira N, Badimon L, Boluda S, Potempa L, Sanfeliu C, de Vera N, Krupinski J.	Brain Pathol. 2010	PMID:19170684
Generation of human inflammation-resistant endothelial progenitor cells by A20 gene transfer.	Liu JW, Dunoyer-Geindre S, Blot-Chabaud M, Sabatier F, Fish RJ, Bounameaux H, Dignat-George F, Kruithof EK.	J Vasc Res. 2010	PMID:19851077
Inhibition of neuropilin-1 by RNA-interference and its angiostatic potential in the treatment of hepatocellular carcinoma.	Raskopf E, Vogt A, Standop J, Sauerbruch T, Schmitz V.	Z Gastroenterol. 2010	PMID:20072992
Prorenin induces ERK activation in endothelial cells to enhance neovascularization independently of the renin-angiotensin system.	Uraoka M, Ikeda K, Nakagawa Y, Koide M, Akakabe Y, Nakano-Kurimoto R, Takahashi T, Matoba S, Yamada H, Okigaki M, Matsubara H.	Biochem Biophys Res Commun. 2009	PMID:19879243
Aspergillus fumigatus inhibits angiogenesis through the production of gliotoxin and other secondary metabolites.	Ben-Ami R, Lewis RE, Leventakos K, Kontoyiannis DP.	Blood. 2009	PMID:19843884 | PMCID:PMC2925388
Astrocyte elevated gene-1 (AEG-1) functions as an oncogene and regulates angiogenesis.	Emdad L, Lee SG, Su ZZ, Jeon HY, Boukerche H, Sarkar D, Fisher PB.	Proc Natl Acad Sci U S A. 2009	PMID:19940250 | PMCID:PMC2795510
Functionally defining the endothelial transcriptome, from Robo4 to ECSCR.	Verissimo AR, Herbert JM, Heath VL, Legg JA, Sheldon H, Andre M, Swain RK, Bicknell R.	Biochem Soc Trans. 2009	PMID:19909249
Vascular endothelial growth factor-C (VEGF-C) promotes angiogenesis by induction of COX-2 in leukemic cells via the VEGF-R3/JNK/AP-1 pathway.	Chien MH, Ku CC, Johansson G, Chen MW, Hsiao M, Su JL, Inoue H, Hua KT, Wei LH, Kuo ML.	Carcinogenesis. 2009	PMID:19825968
Robo1: a potential role in ocular angiogenesis.	Huang L, Xu Y, Yu W, Li X, Liqun C, He X, Peiying H.	Curr Eye Res. 2009	PMID:19958120

The fungal secondary metabolite trichodimerol inhibits TGF-Î² dependent cellular effects and tube formation of MDA-MB-231 cells.	Serwe A, Anke T, Erkel G.	Invest New Drugs. 2009	PMID:19009233
Age-dependent vascular endothelial growth factor expression and angiogenic capability of bladder smooth muscle cells: implications for cell-seeded technology in bladder tissue engineering.	Azzarello J, Kropp BP, Fung KM, Lin HK.	J Tissue Eng Regen Med. 2009	PMID:19685443
Cyclic strain disrupts endothelial network formation on Matrigel.	Wilson CJ, Kasper G, SchÃ¼tz MA, Duda GN.	Microvasc Res. 2009	PMID:19695270
Stable knockdown of heparanase expression in gastric cancer cells in vitro.	Zheng LD, Jiang GS, Pu JR, Mei H, Dong JH, Hou XH, Tong QS.	World J Gastroenterol. 2009	PMID:19916174 | PMCID:PMC2778100
Neuromedin B induces angiogenesis via activation of ERK and Akt in endothelial cells.	Park HJ, Kim SR, Bae SK, Choi YK, Bae YH, Kim EC, Kim WJ, Jang HO, Yun I, Kim YM, Bae MK.	Exp Cell Res. 2009	PMID:19703440
PI3K, Rho, and ROCK play a key role in hypoxia-induced ATP release and ATP-stimulated angiogenic responses in pulmonary artery vasa vasorum endothelial cells.	Woodward HN, Anwar A, Riddle S, Taraseviciene-Stewart L, Fragoso M, Stenmark KR, Gerasimovskaya EV.	Am J Physiol Lung Cell Mol Physiol. 2009	PMID:19684203 | PMCID:PMC2777489
Genetic engineering with endothelial nitric oxide synthase improves functional properties of endothelial progenitor cells from patients with coronary artery disease: an in vitro study.	Kaur S, Kumar TR, Uruno A, Sugawara A, Jayakumar K, Kartha CC.	Basic Res Cardiol. 2009	PMID:19479297
Visfatin through STAT3 activation enhances IL-6 expression that promotes endothelial angiogenesis.	Kim JY, Bae YH, Bae MK, Kim SR, Park HJ, Wee HJ, Bae SK.	Biochim Biophys Acta. 2009	PMID:19751774
Effects of an endothelial cell-conditioned medium on the hematopoietic and endothelial differentiation of embryonic stem cells.	Sun X, Cheng L, Duan H, Lu G.	Cell Biol Int. 2009	PMID:19715766
Variable effects of alpha v suppression on VEGFR-2 expression in endothelial cells of different vascular beds.	Dardik R, Livnat T, Seligsohn U.	Thromb Haemost. 2009	PMID:19888537
The Hedgehog transcription factor Gli3 modulates angiogenesis.	Renault MA, Roncalli J, Tongers J, Misener S, Thorne T, Jujo K, Ito A, Clarke T, Fung C, Millay M, Kamide C, Scarpelli A, Klyachko E, Losordo DW.	Circ Res. 2009	PMID:19729595 | PMCID:PMC3175353
Enhancement of angiogenic and vasculogenic potential of endothelial progenitor cells by haptoglobin.	Park SJ, Baek SH, Oh MK, Choi SH, Park EH, Kim NH, Shin JC, Kim IS.	FEBS Lett. 2009	PMID:19751729
Antiangiogenic properties of silver nanoparticles.	Gurunathan S, Lee KJ, Kalishwaralal K, Sheikpranbabu S, Vaidyanathan R, Eom SH.	Biomaterials. 2009	PMID:19698986
Oligosaccharides of hyaluronan induce angiogenesis through distinct CD44 and RHAMM-mediated signalling pathways involving Cdc2 and gamma-adducin.	Matou-Nasri S, Gaffney J, Kumar S, Slevin M.	Int J Oncol. 2009	PMID:19724912
Lack of inhibitory effects of the anti-fibrotic drug imatinib on endothelial cell functions in vitro and in vivo.	Venalis P, Maurer B, Akhmetshina A, Busch N, Dees C, StÃ¼rzl M, Zwerina J, JÃ¼ngel A, Gay S, Schett G, Distler O, Distler JH.	J Cell Mol Med. 2009	PMID:18774958
Regulatory T cells negatively regulate neovasculature of airway remodeling via DLL4-Notch signaling.	Huang MT, Dai YS, Chou YB, Juan YH, Wang CC, Chiang BL.	J Immunol. 2009	PMID:19752226
Calpain inhibitor SNJ-1945 attenuates events prior to angiogenesis in cultured human retinal endothelial cells.	Ma H, Tochigi A, Shearer TR, Azuma M.	J Ocul Pharmacol Ther. 2009	PMID:19857102 | PMCID:PMC2958445
Systemic sclerosis-endothelial cell antiangiogenic pentraxin 3 and matrix metalloprotease 12 control human breast cancer tumor vascularization and development in mice.	Margheri F, SerratÃ¬ S, Lapucci A, Anastasia C, Giusti B, Pucci M, Torre E, Bianchini F, Calorini L, Albini A, Ventura A, Fibbi G, Del Rosso M.	Neoplasia. 2009	PMID:19794969 | PMCID:PMC2745676
Statins and angiogenesis: is it about connections?	Khaidakov M, Wang W, Khan JA, Kang BY, Hermonat PL, Mehta JL.	Biochem Biophys Res Commun. 2009	PMID:19615978
Inhibitory effects of polysaccharide extract from Spirulina platensis on corneal neovascularization.	Yang L, Wang Y, Zhou Q, Chen P, Wang Y, Wang Y, Liu T, Xie L.	Mol Vis. 2009	PMID:19784394 | PMCID:PMC2751803
The Monascus metabolite monacolin K reduces tumor progression and metastasis of Lewis lung carcinoma cells.	Ho BY, Pan TM.	J Agric Food Chem. 2009	PMID:19754167
delta-Tocotrienol suppresses VEGF induced angiogenesis whereas alpha-tocopherol does not.	Shibata A, Nakagawa K, Sookwong P, Tsuduki T, Oikawa S, Miyazawa T.	J Agric Food Chem. 2009	PMID:19702331
The preadipocyte factor" DLK1 marks adult mouse adipose tissue residing vascular cells that lack in vitro adipogenic differentiation potential."	Andersen DC, Jensen L, SchrÃ¸der HD, Jensen CH.	FEBS Lett. 2009	PMID:19665021
Intermedin is a new angiogenic growth factor.	Smith RS Jr, Gao L, Bledsoe G, Chao L, Chao J.	Am J Physiol Heart	PMID:19592612 |

		Circ Physiol. 2009	PMCID:PMC2755985
Benzene metabolite hydroquinone up-regulates chondromodulin-I and inhibits tube formation in human bone marrow endothelial cells.	Zhou H, Kepa JK, Siegel D, Miura S, Hiraki Y, Ross D.	Mol Pharmacol. 2009	PMID:19525446 | PMCID:PMC2730389
Title	Description	ShortDetails	Identifiers
B-type natriuretic peptide enhances vasculogenesis by promoting number and functional properties of early endothelial progenitor cells.	Shmilovich H, Ben-Shoshan J, Tal R, Afek A, Barshack I, Maysel-Auslander S, Harats D, Keren G, George J.	Tissue Eng Part A. 2009	PMID:19275472
Ligustrazine inhibits B16F10 melanoma metastasis and suppresses angiogenesis induced by Vascular Endothelial Growth Factor.	Chen L, Lu Y, Wu JM, Xu B, Zhang LJ, Gao M, Zheng SZ, Wang AY, Zhang CB, Zhang WW, Lei N.	Biochem Biophys Res Commun. 2009	PMID:19523924
[Antiangiogenic Effect of Oyster Polypeptide (OPP).].	Wang Z, Liu J, Su A, Sun M, Wang C.	Zhongguo Fei Ai Za Zhi. 2009	PMID:20719168
Acidic mucopolysaccharide from Holothuria leucospilota has antitumor effect by inhibiting angiogenesis and tumor cell invasion in vivo and in vitro.	Zhang W, Lu Y, Xu B, Wu J, Zhang L, Gao M, Zheng S, Wang A, Zhang C, Chen L, Lei N.	Cancer Biol Ther. 2009	PMID:19483477
EPOX inhibits angiogenesis by degradation of Mcl-1 through ERK inactivation.	Sun HL, Tsai AC, Pan SL, Ding Q, Yamaguchi H, Lin CN, Hung MC, Teng CM.	Clin Cancer Res. 2009	PMID:19622586
Bone morphogenetic protein 4 is induced in hepatocellular carcinoma by hypoxia and promotes tumour progression.	Maegdefrau U, Amann T, Winklmeier A, Braig S, Schubert T, Weiss TS, Schardt K, Warnecke C, Hellerbrand C, Bosserhoff AK.	J Pathol. 2009	PMID:19431154
[Study on antiangiogenesis effect of Vaccaria segetalis].	Feng L, Hua H, Qiu LY, Zhang LF, Jin J.	Zhong Yao Cai. 2009	PMID:19960951
Antiangiogenic effect of celastrol on the growth of human glioma: an in vitro and in vivo study.	Zhou YX, Huang YL.	Chin Med J (Engl). 2009	PMID:19719969
Decursin and decursinol inhibit VEGF-induced angiogenesis by blocking the activation of extracellular signal-regulated kinase and c-Jun N-terminal kinase.	Son SH, Kim MJ, Chung WY, Son JA, Kim YS, Kim YC, Kang SS, Lee SK, Park KK.	Cancer Lett. 2009	PMID:19307054
Breast cancer cells stimulate osteoprotegerin (OPG) production by endothelial cells through direct cell contact.	Reid PE, Brown NJ, Holen I.	Mol Cancer. 2009	PMID:19604388 | PMCID:PMC2719583
Paeonol exerts anti-angiogenic and anti-metastatic activities through downmodulation of Akt activation and inactivation of matrix metalloproteinases.	Kim SA, Lee HJ, Ahn KS, Lee HJ, Lee EO, Ahn KS, Choi SH, Jung SJ, Kim JY, Baek N, Kim SH.	Biol Pharm Bull. 2009	PMID:19571375
The soluble form of the cancer-associated L1 cell adhesion molecule is a pro-angiogenic factor.	Friedli A, Fischer E, Novak-Hofer I, Cohrs S, Ballmer-Hofer K, Schubiger PA, Schibli R, GrÃ¼nberg J.	Int J Biochem Cell Biol. 2009	PMID:19401151
Pim-3 is expressed in endothelial cells and promotes vascular tube formation.	Zhang P, Wang H, Min X, Wang Y, Tang J, Cheng J, Li D, Chen X, Cheng F, Wang N, Yang H.	J Cell Physiol. 2009	PMID:19229879
Angiogenic activity in sera of patients with systemic lupus erythematosus.	Sakly N, Mirshahi P, Ducros E, Soria J, Ghedira I, Mirshahi M.	Lupus. 2009	PMID:19502266
Influence of adult mesenchymal stem cells on in vitro vascular formation.	Sorrell JM, Baber MA, Caplan AI.	Tissue Eng Part A. 2009	PMID:19196139 | PMCID:PMC2792097
Reconstruction of engineered uterine tissues containing smooth muscle layer in collagen/matrigel scaffold in vitro.	LÃ¼ SH, Wang HB, Liu H, Wang HP, Lin QX, Li DX, Song YX, Duan CM, Feng LX, Wang CY.	Tissue Eng Part A. 2009	PMID:19061433
Anti-angiogenic effects and mechanisms of polysaccharides from Antrodia cinnamomea with different molecular weights.	Yang CM, Zhou YJ, Wang RJ, Hu ML.	J Ethnopharmacol. 2009	PMID:19501273
In vitro and in vivo anti-angiogenic activities and inhibition of hormone-dependent and -independent breast cancer cells by ceramide methylaminoethylphosphonate.	Chintalapati M, Truax R, Stout R, Portier R, Losso JN.	J Agric Food Chem. 2009	PMID:19476359
The 15(S)-hydroxyeicosatetraenoic acid-induced angiogenesis requires Janus kinase 2-signal transducer and activator of transcription-5B-dependent expression of interleukin-8.	Cheranov SY, Wang D, Kundumani-Sridharan V, Karpurapu M, Zhang Q, Chava KR, Rao GN.	Blood. 2009	PMID:19349617 | PMCID:PMC2700333
Visfatin activates eNOS via Akt and MAP kinases and improves endothelial cell function and angiogenesis in vitro and in vivo: translational implications for atherosclerosis.	Lovren F, Pan Y, Shukla PC, Quan A, Teoh H, Szmitko PE, Peterson MD, Gupta M, Al-Omran M, Verma S.	Am J Physiol Endocrinol Metab. 2009	PMID:19351806
Role of the low-affinity leukotriene B4 receptor BLT2 in VEGF-induced angiogenesis.	Kim GY, Lee JW, Cho SH, Seo JM, Kim JH.	Arterioscler Thromb Vasc Biol. 2009	PMID:19286633
ADAM17 promotes breast cancer cell malignant phenotype through EGFR-PI3K-AKT activation.	Zheng X, Jiang F, Katakowski M, Zhang ZG, Lu QE, Chopp M.	Cancer Biol Ther. 2009	PMID:19395875 | PMCID:PMC2766867

Secreted frizzle-related protein 2 stimulates angiogenesis via a calcineurin/NFAT signaling pathway.	Courtwright A, Siamakpour-Reihani S, Arbiser JL, Banet N, Hilliard E, Fried L, Livasy C, Ketelsen D, Nepal DB, Perou CM, Patterson C, Klauber-Demore N.	Cancer Res. 2009	PMID:19458075 | PMCID:PMC2699405
Vasculogenic mimicry of acute leukemic bone marrow stromal cells.	Mirshahi P, Rafii A, Vincent L, Berthaut A, Varin R, Kalantar G, Marzac C, Calandini OA, Marie JP, Soria C, Soria J, Mirshahi M.	Leukemia. 2009	PMID:19340002
Expression of Robo4 in the fibrovascular membranes from patients with proliferative diabetic retinopathy and its role in RF/6A and RPE cells.	Huang L, Yu W, Li X, Xu Y, Niu L, He X, Dong J, Yan Z.	Mol Vis. 2009	PMID:19495426 | PMCID:PMC2689875
Insulin-like growth factor binding protein-7 (IGFBP7) blocks vascular endothelial cell growth factor (VEGF)-induced angiogenesis in human vascular endothelial cells.	Tamura K, Hashimoto K, Suzuki K, Yoshie M, Kutsukake M, Sakurai T.	Eur J Pharmacol. 2009	PMID:19374835
Ex vivo expanded haematopoietic progenitor cells improve dermal wound healing by paracrine mechanisms.	Templin C, Grote K, Schledzewski K, Ghadri JR, Schnabel S, Napp LC, Schieffer B, Kurzen H, Goerdt S, Landmesser U, Koenen W, Faulhaber J.	Exp Dermatol. 2009	PMID:19320744
IL-1alpha secreted by colon cancer cells enhances angiogenesis: the relationship between IL-1alpha release and tumor cells' potential for liver metastasis.	Matsuo Y, Sawai H, Ma J, Xu D, Ochi N, Yasuda A, Takahashi H, Funahashi H, Takeyama H.	J Surg Oncol. 2009	PMID:19204921
Ceramide kinase deficiency impairs microendothelial cell angiogenesis in vitro.	Niwa S, Graf C, Bornancin F.	Microvasc Res. 2009	PMID:19323974
Ginger's (Zingiber officinale Roscoe) inhibition of rat colonic adenocarcinoma cells proliferation and angiogenesis in vitro.	Brown AC, Shah C, Liu J, Pham JT, Zhang JG, Jadus MR.	Phytother Res. 2009	PMID:19117330
Stilbene glycosides are natural product inhibitors of FGF-2-induced angiogenesis.	Hussain S, Slevin M, Ahmed N, West D, Choudhary MI, Naz H, Gaffney J.	BMC Cell Biol. 2009	PMID:19389252 | PMCID:PMC2678990
Anti-angiogenic and vascular disrupting effects of C9, a new microtubule-depolymerizing agent.	Ren X, Dai M, Lin LP, Li PK, Ding J.	Br J Pharmacol. 2009	PMID:19302593 | PMCID:PMC2697741
Cortisol inactivation by 11beta-hydroxysteroid dehydrogenase-2 may enhance endometrial angiogenesis via reduced thrombospondin-1 in heavy menstruation.	Rae M, Mohamad A, Price D, Hadoke PW, Walker BR, Mason JI, Hillier SG, Critchley HO.	J Clin Endocrinol Metab. 2009	PMID:19158196
The effect of brain-derived neurotrophic factor on angiogenesis.	Sun C, Hu Y, Chu Z, Huang J, Zhang L.	J Huazhong Univ Sci Technolog Med Sci. 2009	PMID:19399393
Vascular endothelial growth factor C facilitates immune tolerance and endovascular activity of human uterine NK cells at the maternal-fetal interface.	Kalkunte SS, Mselle TF, Norris WE, Wira CR, Sentman CL, Sharma S.	J Immunol. 2009	PMID:19299706 | PMCID:PMC3616376
Fibroblast growth factor 2 promotes endothelial differentiation of adipose tissue-derived stem cells.	Ning H, Liu G, Lin G, Yang R, Lue TF, Lin CS.	J Sex Med. 2009	PMID:19207272 | PMCID:PMC2893032
Newly developed PPAR-alpha agonist (R)-K-13675 inhibits the secretion of inflammatory markers without affecting cell proliferation or tube formation.	Kitajima K, Miura S, Mastuo Y, Uehara Y, Saku K.	Atherosclerosis. 2009	PMID:18606415
Anti-sphingosine-1-phosphate monoclonal antibodies inhibit angiogenesis and sub-retinal fibrosis in a murine model of laser-induced choroidal neovascularization.	Caballero S, Swaney J, Moreno K, Afzal A, Kielczewski J, Stoller G, Cavalli A, Garland W, Hansen G, Sabbadini R, Grant MB.	Exp Eye Res. 2009	PMID:18723015
Development of a novel antimicrobial peptide, AG-30, with angiogenic properties.	Nishikawa T, Nakagami H, Maeda A, Morishita R, Miyazaki N, Ogawa T, Tabata Y, Kikuchi Y, Hayashi H, Tatsu Y, Yumoto N, Tamai K, Tomono K, Kaneda Y.	J Cell Mol Med. 2009	PMID:18410525
A novel role for activating transcription factor-2 in 15(S)-hydroxyeicosatetraenoic acid-induced angiogenesis.	Zhao T, Wang D, Cheranov SY, Karpurapu M, Chava KR, Kundumani-Sridharan V, Johnson DA, Penn JS, Rao GN.	J Lipid Res. 2009	PMID:18849464 | PMCID:PMC2638108
The anti-angiogenic effects of 1-furan-2-yl-3-pyridin-2-yl-propenone are mediated through the suppression of both VEGF production and VEGF-induced signaling.	Park BC, Park SY, Lee JS, Mousa SA, Kim JT, Kwak MK, Kang KW, Lee ES, Choi HG, Yong CS, Kim JA.	Vascul Pharmacol. 2009	PMID:19068239
Signal transducers and activators of transcription mediate fibroblast growth factor-induced vascular endothelial morphogenesis.	Yang X, Qiao D, Meyer K, Friedl A.	Cancer Res. 2009	PMID:19176400 | PMCID:PMC2650265
CXCL8/IL-8 and CXCL12/SDF-1alpha co-operatively promote invasiveness and angiogenesis in pancreatic cancer.	Matsuo Y, Ochi N, Sawai H, Yasuda A, Takahashi H, Funahashi H, Takeyama H, Tong Z, Guha S.	Int J Cancer. 2009	PMID:19035451 | PMCID:PMC2684108
Sulforaphane stimulates activation of proapoptotic protein bax leading to apoptosis of endothelial progenitor cells.	Nishikawa T, Tsuno NH, Tsuchiya T, Yoneyama S, Yamada J, Shuno Y, Okaji Y, Tanaka J, Kitayama J, Takahashi K, Nagawa H.	Ann Surg Oncol. 2009	PMID:19034579
Anti-angiogenic and anti-metastatic effects of beta-1,3-D-glucan purified from Hanabiratake, Sparassis crispa.	Yamamoto K, Kimura T, Sugitachi A, Matsuura N.	Biol Pharm Bull. 2009	PMID:19182386

Cooperative effect of roscovitine and irradiation targets angiogenesis and induces vascular destabilization in human breast carcinoma.	Maggiorella L, Aubel C, Haton C, Milliat F, Connault E, Opolon P, Deutsch E, Bourhis J.	Cell Prolif. 2009	PMID:19143762
Identification of an aberrant cell line among human adipose tissue-derived stem cell isolates.	Ning H, Liu G, Lin G, Garcia M, Li LC, Lue TF, Lin CS.	Differentiation. 2009	PMID:19281777 | PMCID:PMC2893016
Active involvement of Robo1 and Robo4 in filopodia formation and endothelial cell motility mediated via WASP and other actin nucleation-promoting factors.	Sheldon H, Andre M, Legg JA, Heal P, Herbert JM, Sainson R, Sharma AS, Kitajewski JK, Heath VL, Bicknell R.	FASEB J. 2009	PMID:18948384
Caveolin-1 and Rac regulate endothelial capillary-like tubular formation and fenestral contraction in sinusoidal endothelial cells.	Yokomori H, Oda M, Yoshimura K, Nagai T, Fujimaki K, Watanabe S, Hibi T.	Liver Int. 2009	PMID:19067793
Antimicrobial human beta-defensin-2 stimulates migration, proliferation and tube formation of human umbilical vein endothelial cells.	Baroni A, Donnarumma G, Paoletti I, Longanesi-Cattani I, Bifulco K, Tufano MA, Carriero MV.	Peptides. 2009	PMID:19041917
Title	Description	ShortDetails	Identifiers
Morelloflavone, a biflavonoid, inhibits tumor angiogenesis by targeting rho GTPases and extracellular signal-regulated kinase signaling pathways.	Pang X, Yi T, Yi Z, Cho SG, Qu W, Pinkaew D, Fujise K, Liu M.	Cancer Res. 2009	PMID:19147565 | PMCID:PMC2662342
The endothelial cell tube formation assay on basement membrane turns 20: state of the science and the art.	Arnaoutova I, George J, Kleinman HK, Benton G.	Angiogenesis. 2009	PMID:19399631
Cell surface nucleolin antagonist causes endothelial cell apoptosis and normalization of tumor vasculature.	Fogal V, Sugahara KN, Ruoslahti E, Christian S.	Angiogenesis. 2009	PMID:19225898
Des-gamma-carboxy prothrombin stimulates human vascular endothelial cell growth and migration.	Wang SB, Cheng YN, Cui SX, Zhong JL, Ward SG, Sun LR, Chen MH, Kokudo N, Tang W, Qu XJ.	Clin Exp Metastasis. 2009	PMID:19263229
Anti-angiogenic property of zoledronic acid by inhibition of endothelial progenitor cell differentiation.	Yamada J, Tsuno NH, Kitayama J, Tsuchiya T, Yoneyama S, Asakage M, Okaji Y, Shuno Y, Nishikawa T, Tanaka J, Takahashi K, Nagawa H.	J Surg Res. 2009	PMID:18619615
Hypoxia-inducible factor-1alpha and -2alpha additively promote endothelial vasculogenic properties.	Ben-Shoshan J, Maysel-Auslender S, Luboshits G, Barshack I, Polak-Charcon S, Tzahor E, Keren G, George J.	J Vasc Res. 2009	PMID:19077391
Tube formation: an in vitro matrigel angiogenesis assay.	Ponce ML.	Methods Mol Biol. 2009	PMID:19301671
Targeting of integrin-linked kinase with a small interfering RNA inhibits endothelial cell migration, proliferation and tube formation in vitro.	Guo L, Yu W, Li X, Zhao G, He P.	Ophthalmic Res. 2009	PMID:19672130
In vitro assay of angiogenesis: inhibition of capillary tube formation.	McGonigle S, Shifrin V.	Curr Protoc Pharmacol. 2008	PMID:22294219
Glioma cells enhance endothelial progenitor cell angiogenesis via VEGFR-2, not VEGFR-1.	Zhang J, Zhao P, Fu Z, Chen X, Liu N, Lu A, Li R, Shi L, Pu P, Kang C, You Y.	Oncol Rep. 2008	PMID:19020728
Tumor endothelial cell targeted cyclic RGD-modified heparin derivative: inhibition of angiogenesis and tumor growth.	Park K, Kim YS, Lee GY, Park RW, Kim IS, Kim SY, Byun Y.	Pharm Res. 2008	PMID:18581207
Glioblastoma-secreted factors induce IGFBP7 and angiogenesis by modulating Smad-2-dependent TGF-beta signaling.	Pen A, Moreno MJ, Durocher Y, Deb-Rinker P, Stanimirovic DB.	Oncogene. 2008	PMID:18711401
Epoxyeicosatrienoic acids are part of the VEGF-activated signaling cascade leading to angiogenesis.	Webler AC, Michaelis UR, Popp R, Barbosa-Sicard E, Murugan A, Falck JR, Fisslthaler B, Fleming I.	Am J Physiol Cell Physiol. 2008	PMID:18787075 | PMCID:PMC2584984
Progesterone inhibits human endothelial cell proliferation through a p53-dependent pathway.	Hsu SP, Ho PY, Juan SH, Liang YC, Lee WS.	Cell Mol Life Sci. 2008	PMID:18850315
Circulating endothelial progenitor cells as a pathogenetic marker of moyamoya disease.	Jung KH, Chu K, Lee ST, Park HK, Kim DH, Kim JH, Bahn JJ, Song EC, Kim M, Lee SK, Roh JK.	J Cereb Blood Flow Metab. 2008	PMID:18612318
Nondenatured soy extracts reduce UVB-induced skin damage via multiple mechanisms.	Chen N, Scarpa R, Zhang L, Seiberg M, Lin CB.	Photochem Photobiol. 2008	PMID:18627522
T1alpha/podoplanin is essential for capillary morphogenesis in lymphatic endothelial cells.	Navarro A, Perez RE, Rezaiekhaligh M, Mabry SM, Ekekezie II.	Am J Physiol Lung Cell Mol Physiol. 2008	PMID:18658274
Gene expression profiles by microarray analysis during matrigel-induced tube formation in a human extravillous trophoblast cell line: comparison with endothelial cells.	Fukushima K, Murata M, Hachisuga M, Tsukimori K, Seki H, Takeda S, Kato K, Wake N.	Placenta. 2008	PMID:18783822
In vitro and in vivo evidence for lack of endovascular remodeling by third trimester trophoblasts.	Kalkunte S, Lai Z, Tewari N, Chichester C, Romero R, Padbury J, Sharma S.	Placenta. 2008	PMID:18775564 | PMCID:PMC3611242
Anti-angiogenic effect of high doses of ascorbic acid.	Mikirova NA, Ichim TE, Riordan NH.	J Transl Med. 2008	PMID:18789157 | PMCID:PMC2562367

Oxidized low density lipoprotein impairs endothelial progenitor cell function by downregulation of E-selectin and integrin alpha(v)beta5.	Di Santo S, Diehm N, Ortmann J, VÃ¶lzmann J, Yang Z, Keo HH, Baumgartner I, Kalka C.	Biochem Biophys Res Commun. 2008	PMID:18590706
ECSM2, an endothelial specific filamin a binding protein that mediates chemotaxis.	Armstrong LJ, Heath VL, Sanderson S, Kaur S, Beesley JF, Herbert JM, Legg JA, Poulsom R, Bicknell R.	Arterioscler Thromb Vasc Biol. 2008	PMID:18556573
Interferon regulatory factor-1 (IRF-1) regulates VEGF-induced angiogenesis in HUVECs.	Lee JH, Chun T, Park SY, Rho SB.	Biochim Biophys Acta. 2008	PMID:18472010
Overexpression of netrin-1 induces neovascularization in the adult mouse brain.	Fan Y, Shen F, Chen Y, Hao Q, Liu W, Su H, Young WL, Yang GY.	J Cereb Blood Flow Metab. 2008	PMID:18461079 | PMCID:PMC2581494
Tie2-R849W mutant in venous malformations chronically activates a functional STAT1 to modulate gene expression.	Hu HT, Huang YH, Chang YA, Lee CK, Jiang MJ, Wu LW.	J Invest Dermatol. 2008	PMID:18401423
Inhibition of protein kinase CK2 suppresses angiogenesis and hematopoietic stem cell recruitment to retinal neovascularization sites.	Kramerov AA, Saghizadeh M, Caballero S, Shaw LC, Li Calzi S, Bretner M, Montenarh M, Pinna LA, Grant MB, Ljubimov AV.	Mol Cell Biochem. 2008	PMID:18612802 | PMCID:PMC2913688
Sphingosine-1-phosphate promotes lymphangiogenesis by stimulating S1P1/Gi/PLC/Ca2+ signaling pathways.	Yoon CM, Hong BS, Moon HG, Lim S, Suh PG, Kim YK, Chae CB, Gho YS.	Blood. 2008	PMID:18541717 | PMCID:PMC2515114
Dual functions of a monoclonal antibody against cell surface F1F0 ATP synthase on both HUVEC and tumor cells.	Zhang X, Gao F, Yu LL, Peng Y, Liu HH, Liu JY, Yin M, Ni J.	Acta Pharmacol Sin. 2008	PMID:18664327
Prolonged use of high-dose morphine impairs angiogenesis and mobilization of endothelial progenitor cells in mice.	Lam CF, Chang PJ, Huang YS, Sung YH, Huang CC, Lin MW, Liu YC, Tsai YC.	Anesth Analg. 2008	PMID:18633053
Tumor anti-angiogenic effect and mechanism of action of delta-tocotrienol.	Shibata A, Nakagawa K, Sookwong P, Tsuzuki T, Oikawa S, Miyazawa T.	Biochem Pharmacol. 2008	PMID:18599020
Human endothelial precursor cells express tumor endothelial marker 1/endosialin/CD248.	Bagley RG, Rouleau C, St Martin T, Boutin P, Weber W, Ruzek M, Honma N, Nacht M, Shankara S, Kataoka S, Ishida I, Roberts BL, Teicher BA.	Mol Cancer Ther. 2008	PMID:18723498
The effects of growth factors on the proliferation and in vitro angiogenesis of human macular inner choroidal endothelial cells.	Browning AC, Dua HS, Amoaku WM.	Br J Ophthalmol. 2008	PMID:18577655
Antimigratory effect of TK1-2 is mediated in part by interfering with integrin alpha2beta1.	Kim HK, Oh DS, Lee SB, Ha JM, Joe YA.	Mol Cancer Ther. 2008	PMID:18645023
Antiangiogenic vinflunine affects EB1 localization and microtubule targeting to adhesion sites.	HonorÃ© S, Pagano A, Gauthier G, Bourgarel-Rey V, Verdier-Pinard P, Civiletti K, Kruczynski A, Braguer D.	Mol Cancer Ther. 2008	PMID:18645018
Deguelin inhibits human hepatocellular carcinoma by antiangiogenesis and apoptosis.	Lee JH, Lee DH, Lee HS, Choi JS, Kim KW, Hong SS.	Oncol Rep. 2008	PMID:18575727
Effect of nicotine on human umbilical vein endothelial cells (HUVECs) migration and angiogenesis.	Park YJ, Lee T, Ha J, Jung IM, Chung JK, Kim SJ.	Vascul Pharmacol. 2008	PMID:18571475
The first but not the second thrombospondin type 1 repeat of ADAMTS5 functions as an angiogenesis inhibitor.	Sharghi-Namini S, Fan H, Sulochana KN, Potturi P, Xiang W, Chong YS, Wang Z, Yang H, Ge R.	Biochem Biophys Res Commun. 2008	PMID:18433719
An essential role for SRC-activated STAT-3 in 14,15-EET-induced VEGF expression and angiogenesis.	Cheranov SY, Karpurapu M, Wang D, Zhang B, Venema RC, Rao GN.	Blood. 2008	PMID:18408167 | PMCID:PMC2424155
Thrombin up-regulates cathepsin D which enhances angiogenesis, growth, and metastasis.	Hu L, Roth JM, Brooks P, Luty J, Karpatkin S.	Cancer Res. 2008	PMID:18559512
p190B RhoGAP regulates endothelial-cell-associated proteolysis through MT1-MMP and MMP2.	Guegan F, Tatin F, Leste-Lasserre T, Drutel G, Genot E, Moreau V.	J Cell Sci. 2008	PMID:18505793
Androgen receptor is causally involved in the homeostasis of the human prostate endothelial cell.	Godoy A, Watts A, Sotomayor P, Montecinos VP, Huss WJ, Onate SA, Smith GJ.	Endocrinology. 2008	PMID:18292195 | PMCID:PMC2408806
KR-31831, benzopyran derivative, inhibits VEGF-induced angiogenesis of HUVECs through suppressing KDR expression.	Park SY, Seo EH, Song HS, Jung SY, Lee YK, Yi KY, Yoo SE, Kim YJ.	Int J Oncol. 2008	PMID:18497993
A fraction of methylene chloride from Geum japonicum Thunberg inhibits tumor metastatic and angiogenic potential.	Heo JC, Son M, Woo SU, Kweon MA, Yoon EK, Lee HK, Choi WS, Cho KJ, Lee SH.	Oncol Rep. 2008	PMID:18497943
Estrogen-induced redox sensitive Id3 signaling controls the growth of vascular cells.	Felty Q, Porther N.	Atherosclerosis. 2008	PMID:18281048
Activation of sphingosine kinase-1 mediates induction of endothelial cell proliferation and angiogenesis by epoxyeicosatrienoic acids.	Yan G, Chen S, You B, Sun J.	Cardiovasc Res. 2008	PMID:18192241

Protein kinase C alpha promotes angiogenic activity of human endothelial cells via induction of vascular endothelial growth factor.	Xu H, Czerwinski P, Hortmann M, Sohn HY, FÃ¶rstermann U, Li H.	Cardiovasc Res. 2008	PMID:18056764
Clodronate inhibits angiogenesis in vitro and in vivo.	Ribatti D, Maruotti N, Nico B, Longo V, Mangieri D, Vacca A, Cantatore FP.	Oncol Rep. 2008	PMID:18425365
Circulating endothelial progenitor cells as a new marker of endothelial dysfunction or repair in acute stroke.	Chu K, Jung KH, Lee ST, Park HK, Sinn DI, Kim JM, Kim DH, Kim JH, Kim SJ, Song EC, Kim M, Lee SK, Roh JK.	Stroke. 2008	PMID:18356550
Role of JNK in network formation of human lung microvascular endothelial cells.	Medhora M, Dhanasekaran A, Pratt PF Jr, Cook CR, Dunn LK, Gruenloh SK, Jacobs ER.	Am J Physiol Lung Cell Mol Physiol. 2008	PMID:18263671 | PMCID:PMC2904476
Transcription factor Erg regulates angiogenesis and endothelial apoptosis through VE-cadherin.	Birdsey GM, Dryden NH, Amsellem V, Gebhardt F, Sahnan K, Haskard DO, Dejana E, Mason JC, Randi AM.	Blood. 2008	PMID:18195090 | PMCID:PMC2275018
Title	Description	ShortDetails	Identifiers
Angiogenic potential of 3-nitro-4-hydroxy benzene arsonic acid (roxarsone).	Basu P, Ghosh RN, Grove LE, Klei L, Barchowsky A.	Environ Health Perspect. 2008	PMID:18414637 | PMCID:PMC2290980
The Dickkopf-homolog 3 is expressed in tumor endothelial cells and supports capillary formation.	Untergasser G, Steurer M, Zimmermann M, Hermann M, Kern J, Amberger A, Gastl G, Gunsilius E.	Int J Cancer. 2008	PMID:18033687
Xanthine oxidase interaction with vascular endothelial growth factor in human endothelial cell angiogenesis.	Kou B, Ni J, Vatish M, Singer DR.	Microcirculation. 2008	PMID:18386220
Aquaporin 1 is required for hypoxia-inducible angiogenesis in human retinal vascular endothelial cells.	Kaneko K, Yagui K, Tanaka A, Yoshihara K, Ishikawa K, Takahashi K, Bujo H, Sakurai K, Saito Y.	Microvasc Res. 2008	PMID:18275976
Hypoxia inducible factor 1 alpha regulates matrigel-induced endovascular differentiation under normoxia in a human extravillous trophoblast cell line.	Fukushima K, Murata M, Hachisuga M, Tsukimori K, Seki H, Takeda S, Asanoma K, Wake N.	Placenta. 2008	PMID:18342368
Characterizing endothelial cells derived from the murine embryonic stem cell line CCE.	Fathi F, Kermani AJ, Pirmoradi L, Mowla SJ, Asahara T.	Rejuvenation Res. 2008	PMID:18393656
Inhibition of telomerase in the endothelial cells disrupts tumor angiogenesis in glioblastoma xenografts.	Falchetti ML, Mongiardi MP, Fiorenzo P, Petrucci G, Pierconti F, D'Agnano I, D'Alessandris G, Alessandri G, Gelati M, Ricci-Vitiani L, Maira G, Larocca LM, Levi A, Pallini R.	Int J Cancer. 2008	PMID:18027853
Identification and characterization of small-molecule inhibitors of Tie2 kinase.	Liu J, Lin TH, Cole AG, Wen R, Zhao L, Brescia MR, Jacob B, Hussain Z, Appell KC, Henderson I, Webb ML.	FEBS Lett. 2008	PMID:18267118
Evidence for the involvement of miRNA in redox regulated angiogenic response of human microvascular endothelial cells.	Shilo S, Roy S, Khanna S, Sen CK.	Arterioscler Thromb Vasc Biol. 2008	PMID:18258815
Small interfering RNA knockdown of mini-TyrRS and mini-TrpRS effects angiogenesis in human umbilical vein endothelial cells in hypoxic culture.	Zeng R, Chen YC, Zeng Z, Liu R, Qiang O, Jiang XF, Liu XX, Li X, Wang HY.	Cytotechnology. 2008	PMID:19002860 | PMCID:PMC2553632
In vitro hyperglycemia or a diabetic intrauterine environment reduces neonatal endothelial colony-forming cell numbers and function.	Ingram DA, Lien IZ, Mead LE, Estes M, Prater DN, Derr-Yellin E, DiMeglio LA, Haneline LS.	Diabetes. 2008	PMID:18086900
Quercetin inhibits choroidal and retinal angiogenesis in vitro.	Chen Y, Li XX, Xing NZ, Cao XG.	Graefes Arch Clin Exp Ophthalmol. 2008	PMID:18087712
Wnt2 acts as a cell type-specific, autocrine growth factor in rat hepatic sinusoidal endothelial cells cross-stimulating the VEGF pathway.	Klein D, Demory A, Peyre F, Kroll J, Augustin HG, Helfrich W, Kzhyshkowska J, Schledzewski K, Arnold B, Goerdt S.	Hepatology. 2008	PMID:18302287
Regulation of VEGF-mediated angiogenesis by the Akt/PKB substrate Girdin.	Kitamura T, Asai N, Enomoto A, Maeda K, Kato T, Ishida M, Jiang P, Watanabe T, Usukura J, Kondo T, Costantini F, Murohara T, Takahashi M.	Nat Cell Biol. 2008	PMID:18264090
A comparison of the tube forming potentials of early and late endothelial progenitor cells.	Mukai N, Akahori T, Komaki M, Li Q, Kanayasu-Toyoda T, Ishii-Watabe A, Kobayashi A, Yamaguchi T, Abe M, Amagasa T, Morita I.	Exp Cell Res. 2008	PMID:18083163
Tumor endothelial cell tube formation model for determining anti-angiogenic activity of a tRNA synthetase cytokine.	Zhou Q, Kiosses WB, Liu J, Schimmel P.	Methods. 2008	PMID:18241800
Cheiradone: a vascular endothelial cell growth factor receptor antagonist.	Hussain S, Slevin M, Mesaik MA, Choudhary MI, Elosta AH, Matou S, Ahmed N, West D, Gaffney J.	BMC Cell Biol. 2008	PMID:18230134 | PMCID:PMC2248182
Antiproliferative and antimetastatic effects of the ethanolic extract of Phellinus igniarius (Linnearus: Fries) Quelet.	Song TY, Lin HC, Yang NC, Hu ML.	J Ethnopharmacol. 2008	PMID:17936529

Dihydrotanshinone I inhibits angiogenesis both in vitro and in vivo.	Bian W, Chen F, Bai L, Zhang P, Qin W.	Acta Biochim Biophys Sin (Shanghai). 2008	PMID:18180848
Tetraiodothyroacetic acid, a small molecule integrin ligand, blocks angiogenesis induced by vascular endothelial growth factor and basic fibroblast growth factor.	Mousa SA, Bergh JJ, Dier E, Rebbaa A, O'Connor LJ, Yalcin M, Aljada A, Dyskin E, Davis FB, Lin HY, Davis PJ.	Angiogenesis. 2008	PMID:18080776
Extracellular ATP is a pro-angiogenic factor for pulmonary artery vasa vasorum endothelial cells.	Gerasimovskaya EV, Woodward HN, Tucker DA, Stenmark KR.	Angiogenesis. 2008	PMID:18071915 | PMCID:PMC2480488
Capsiate, a nonpungent capsaicin-like compound, inhibits angiogenesis and vascular permeability via a direct inhibition of Src kinase activity.	Pyun BJ, Choi S, Lee Y, Kim TW, Min JK, Kim Y, Kim BD, Kim JH, Kim TY, Kim YM, Kwon YG.	Cancer Res. 2008	PMID:18172315
Synergism of biochemical and mechanical stimuli in the differentiation of human placenta-derived multipotent cells into endothelial cells.	Wu CC, Chao YC, Chen CN, Chien S, Chen YC, Chien CC, Chiu JJ, Linju Yen B.	J Biomech. 2008	PMID:18190919
Interleukin-6 stimulates circulating blood-derived endothelial progenitor cell angiogenesis in vitro.	Fan Y, Ye J, Shen F, Zhu Y, Yeghiazarians Y, Zhu W, Chen Y, Lawton MT, Young WL, Yang GY.	J Cereb Blood Flow Metab. 2008	PMID:17519976 | PMCID:PMC2581498
PRDM6 is enriched in vascular precursors during development and inhibits endothelial cell proliferation, survival, and differentiation.	Wu Y, Ferguson JE 3rd, Wang H, Kelley R, Ren R, McDonough H, Meeker J, Charles PC, Wang H, Patterson C.	J Mol Cell Cardiol. 2008	PMID:17662997 | PMCID:PMC2683064
z-Guggulsterone, a constituent of Ayurvedic medicinal plant Commiphora mukul, inhibits angiogenesis in vitro and in vivo.	Xiao D, Singh SV.	Mol Cancer Ther. 2008	PMID:18202020
Notch pathway modulation on bone marrow-derived vascular precursor cells regulates their angiogenic and wound healing potential.	Caiado F, Real C, Carvalho T, Dias S.	PLoS One. 2008	PMID:19015735 | PMCID:PMC2582964
Endothelial capillary tube formation and cell proliferation induced by tumor cells are affected by low molecular weight heparins and unfractionated heparin.	Marchetti M, Vignoli A, Russo L, Balducci D, Pagnoncelli M, Barbui T, Falanga A.	Thromb Res. 2008	PMID:17692905
Gambogic acid inhibits angiogenesis through suppressing vascular endothelial growth factor-induced tyrosine phosphorylation of KDR/Flk-1.	Lu N, Yang Y, You QD, Ling Y, Gao Y, Gu HY, Zhao L, Wang XT, Guo QL.	Cancer Lett. 2007	PMID:17920764
Anti-angiogenic properties of a sulindac analogue.	Pyriochou A, Tsigkos S, Vassilakopoulos T, Cottin T, Zhou Z, Gourzoulidou E, Roussos C, Waldmann H, Giannis A, Papapetropoulos A.	Br J Pharmacol. 2007	PMID:17965739 | PMCID:PMC2189991
PEDF-derived synthetic peptides exhibit antitumor activity in an orthotopic model of human osteosarcoma.	Ek ET, Dass CR, Contreras KG, Choong PF.	J Orthop Res. 2007	PMID:17600821
Involvement of gamma-secretase in postnatal angiogenesis.	Hayashi H, Nakagami H, Takami Y, Sato N, Saito Y, Nishikawa T, Mori M, Koriyama H, Tamai K, Morishita R, Kaneda Y.	Biochem Biophys Res Commun. 2007	PMID:17888873
16-kDa prolactin inhibits endothelial cell migration by down-regulating the Ras-Tiam1-Rac1-Pak1 signaling pathway.	Lee SH, Kunz J, Lin SH, Yu-Lee LY.	Cancer Res. 2007	PMID:18006851
JunB is required for IgE-mediated degranulation and cytokine release of mast cells.	Textor B, Licht AH, Tuckermann JP, Jessberger R, Razin E, Angel P, Schorpp-Kistner M, Hartenstein B.	J Immunol. 2007	PMID:17982078
Stress-mediated hormetic modulation of aging, wound healing, and angiogenesis in human cells.	Rattan SI, Sejersen H, Fernandes RA, Luo W.	Ann N Y Acad Sci. 2007	PMID:18056960
Sulfated polymannuroguluronate, a novel anti-AIDS drug candidate, inhibits HIV-1 Tat-induced angiogenesis in Kaposi's sarcoma cells.	Lu CX, Li J, Sun YX, Qi X, Wang QJ, Xin XL, Geng MY.	Biochem Pharmacol. 2007	PMID:17868650
Identification of a novel role of T cells in postnatal vasculogenesis: characterization of endothelial progenitor cell colonies.	Hur J, Yang HM, Yoon CH, Lee CS, Park KW, Kim JH, Kim TY, Kim JY, Kang HJ, Chae IH, Oh BH, Park YB, Kim HS.	Circulation. 2007	PMID:17909106
Differential regulation of thrombospondin-1 expression and antiangiogenesis of ECV304 cells by trichostatin A and helixor A.	Hong S, Chang SY, Yeom DH, Kang JH, Hong KJ.	Anticancer Drugs. 2007	PMID:17704650
Inhibition of Jun NH2-terminal kinases suppresses the growth of experimental head and neck squamous cell carcinoma.	Gross ND, Boyle JO, Du B, Kekatpure VD, Lantowski A, Thaler HT, Weksler BB, Subbaramaiah K, Dannenberg AJ.	Clin Cancer Res. 2007	PMID:17908987
IB05204, a dichloropyridodithienotriazine, inhibits angiogenesis in vitro and in vivo.	MartÃnez-Poveda B, MuÃ±oz-ChÃ¡puli R, RodrÃguez-Nieto S, Quintela JM, FernÃ¡ndez A, Medina MA, Quesada AR.	Mol Cancer Ther. 2007	PMID:17938261
IL-20 is an arteriogenic cytokine that remodels collateral networks and improves functions of ischemic hind limbs.	Tritsaris K, Myren M, Ditlev SB, HÃ¼bschmann MV, van der Blom I, Hansen AJ, Olsen UB, Cao R, Zhang J, Jia T, Wahlberg E, Dissing S, Cao Y.	Proc Natl Acad Sci U S A. 2007	PMID:17878297 | PMCID:PMC1978488
Poly(ADP-ribose) polymerase (PARP) inhibition or PARP-1 gene deletion reduces angiogenesis.	Tentori L, Lacal PM, Muzi A, Dorio AS, Leonetti C, Scarsella M, Ruffini F, Xu W, Min W, Stoppacciaro A, Colarossi C, Wang ZQ, Zhang J, Graziani G.	Eur J Cancer. 2007	PMID:17714938

Circulating endothelial progenitor cells in patients with cardiac syndrome X.	Shmilovich H, Deutsch V, Roth A, Miller H, Keren G, George J.	Heart. 2007	PMID:17194713 | PMCID:PMC1955034
Protein C inhibitor inhibits breast cancer cell growth, metastasis and angiogenesis independently of its protease inhibitory activity.	Asanuma K, Yoshikawa T, Hayashi T, Akita N, Nakagawa N, Hamada Y, Nishioka J, Kamada H, Gabazza EC, Ido M, Uchida A, Suzuki K.	Int J Cancer. 2007	PMID:17450526
Establishment and characterization of Fabry disease endothelial cells with an extended lifespan.	Shen JS, Meng XL, Schiffmann R, Brady RO, Kaneski CR.	Mol Genet Metab. 2007	PMID:17644384 | PMCID:PMC2063578
In vivo inhibition of angiogenesis by interleukin-13 gene therapy in a rat model of rheumatoid arthritis.	Haas CS, Amin MA, Ruth JH, Allen BL, Ahmed S, Pakozdi A, Woods JM, Shahrara S, Koch AE.	Arthritis Rheum. 2007	PMID:17665443
Neridronate inhibits angiogenesis in vitro and in vivo.	Ribatti D, Nico B, Mangieri D, Maruotti N, Longo V, Vacca A, Cantatore FP.	Clin Rheumatol. 2007	PMID:17106617
PI-88 and novel heparan sulfate mimetics inhibit angiogenesis.	Ferro V, Dredge K, Liu L, Hammond E, Bytheway I, Li C, Johnstone K, Karoli T, Davis K, Copeman E, Gautam A.	Semin Thromb Hemost. 2007	PMID:17629854
The establishment of murine blood outgrowth endothelial cells and observations relevant to gene therapy.	Somani A, Nguyen J, Milbauer LC, Solovey A, Sajja S, Hebbel RP.	Transl Res. 2007	PMID:17585861
Curcumin inhibits the formation of capillary-like tubes by rat lymphatic endothelial cells.	Matsuo M, Sakurai H, Koizumi K, Saiki I.	Cancer Lett. 2007	PMID:17197075
Title	Description	ShortDetails	Identifiers
Functional implication of BMP4 expression on angiogenesis in malignant melanoma.	Rothhammer T, Bataille F, Spruss T, Eissner G, Bosserhoff AK.	Oncogene. 2007	PMID:17173062
Optimal reactive oxygen species concentration and p38 MAP kinase are required for coronary collateral growth.	Rocic P, Kolz C, Reed R, Potter B, Chilian WM.	Am J Physiol Heart Circ Physiol. 2007	PMID:17308014
Inhibition of adenovirus-mediated human MAGE-D1 on angiogenesis in vitro and in vivo.	Shen WG, Xue QY, Zhu J, Hu BS, Zhang Y, Wu YD, Su Q.	Mol Cell Biochem. 2007	PMID:17149546
Knockdown of endothelial NOS by lentivirus-mediated short hairpin RNA in hemangioendothelioma cells increases proliferation and tumor formation.	Chen Z, Joshi S, Kauser K, Brooks AR.	Nitric Oxide. 2007	PMID:17452114
Visfatin promotes angiogenesis by activation of extracellular signal-regulated kinase 1/2.	Kim SR, Bae SK, Choi KS, Park SY, Jun HO, Lee JY, Jang HO, Yun I, Yoon KH, Kim YJ, Yoo MA, Kim KW, Bae MK.	Biochem Biophys Res Commun. 2007	PMID:17408594
Calcitonin gene-related peptide (CALCA) is a proangiogenic growth factor in the human placental development.	Dong YL, Reddy DM, Green KE, Chauhan MS, Wang HQ, Nagamani M, Hankins GD, Yallampalli C.	Biol Reprod. 2007	PMID:17267696
15(S)-hydroxyeicosatetraenoic acid-induced angiogenesis requires STAT3-dependent expression of VEGF.	Srivastava K, Kundumani-Sridharan V, Zhang B, Bajpai AK, Rao GN.	Cancer Res. 2007	PMID:17483346
Inhibition of angiogenesis and invasion by 3,3'-diindolylmethane is mediated by the nuclear factor-kappaB downstream target genes MMP-9 and uPA that regulated bioavailability of vascular endothelial growth factor in prostate cancer.	Kong D, Li Y, Wang Z, Banerjee S, Sarkar FH.	Cancer Res. 2007	PMID:17409440
Insulin-like growth factor binding protein-3 induces angiogenesis through IGF-I- and SphK1-dependent mechanisms.	Granata R, Trovato L, Lupia E, Sala G, Settanni F, Camussi G, Ghidoni R, Ghigo E.	J Thromb Haemost. 2007	PMID:17388800
Characterization of endothelial-like cells derived from human mesenchymal stem cells.	Liu JW, Dunoyer-Geindre S, Serre-Beinier V, Mai G, Lambert JF, Fish RJ, Pernod G, Buehler L, Bounameaux H, Kruithof EK.	J Thromb Haemost. 2007	PMID:17229052
Impairment of myocardial angiogenic response in the absence of osteopontin.	Zhao X, Johnson JN, Singh K, Singh M.	Microcirculation. 2007	PMID:17454675
[Isolation, culture and identification of two types of endothelial progenitor cells from peripheral blood in rabbits].	Xiao FY, Zhang HQ, Yu H, Yang DY, Huang WJ, Zhou H.	Xi Bao Yu Fen Zi Mian Yi Xue Za Zhi. 2007	PMID:17428381
Stromal cell-derived factor 1 promotes angiogenesis via a heme oxygenase 1-dependent mechanism.	Deshane J, Chen S, Caballero S, Grochot-Przeczek A, Was H, Li Calzi S, Lach R, Hock TD, Chen B, Hill-Kapturczak N, Siegal GP, Dulak J, Jozkowicz A, Grant MB, Agarwal A.	J Exp Med. 2007	PMID:17339405 | PMCID:PMC1855437
Effects of camptothecin on tumor cell proliferation and angiogenesis when coupled to a bombesin analog used as a targeted delivery vector.	Sun LC, Luo J, Mackey VL, Fuselier JA, Coy DH.	Anticancer Drugs. 2007	PMID:17264768
(-)-Epigallocatechin-3-gallate inhibits growth of gastric cancer by reducing VEGF production and angiogenesis.	Zhu BH, Zhan WH, Li ZR, Wang Z, He YL, Peng JS, Cai SR, Ma JP, Zhang CH.	World J Gastroenterol. 2007	PMID:17451194

Receptor activator of nuclear factor (NF)-kappaB ligand (RANKL) increases vascular permeability: impaired permeability and angiogenesis in eNOS-deficient mice.	Min JK, Cho YL, Choi JH, Kim Y, Kim JH, Yu YS, Rho J, Mochizuki N, Kim YM, Oh GT, Kwon YG.	Blood. 2007	PMID:17038532
Deletion of tetraspanin Cd151 results in decreased pathologic angiogenesis in vivo and in vitro.	Takeda Y, Kazarov AR, Butterfield CE, Hopkins BD, Benjamin LE, Kaipainen A, Hemler ME.	Blood. 2007	PMID:17023588 | PMCID:PMC1794066
[Regulation of axon guidance cue netrin-1 on angiogenesis].	Yang Y, Zou L, Wang Y, Zhang JX.	Zhonghua Yi Xue Za Zhi. 2007	PMID:17459228
Adenovirus-mediated expression of tissue factor pathway inhibitor-2 inhibits endothelial cell migration and angiogenesis.	Ivanciu L, Gerard RD, Tang H, Lupu F, Lupu C.	Arterioscler Thromb Vasc Biol. 2007	PMID:17138934
Stability of angiogenic agents, ginsenoside Rg1 and Re, isolated from Panax ginseng: in vitro and in vivo studies.	Yu LC, Chen SC, Chang WC, Huang YC, Lin KM, Lai PH, Sung HW.	Int J Pharm. 2007	PMID:16962729
CD151 gene delivery increases eNOS activity and induces ECV304 migration, proliferation and tube formation.	Zheng ZZ, Liu ZX.	Acta Pharmacol Sin. 2007	PMID:17184584
T-cadherin suppresses angiogenesis in vivo by inhibiting migration of endothelial cells.	Rubina K, Kalinina N, Potekhina A, Efimenko A, Semina E, Poliakov A, Wilkinson DG, Parfyonova Y, Tkachuk V.	Angiogenesis. 2007	PMID:17486418
Porphyromonas gingivalis, periodontal pathogen, lipopolysaccharide induces angiogenesis via extracellular signal-regulated kinase 1/2 activation in human vascular endothelial cells.	Koo TH, Jun HO, Bae SK, Kim SR, Moon CP, Jeong SK, Kim WS, Kim GC, Jang HO, Yun I, Kim KW, Bae MK.	Arch Pharm Res. 2007	PMID:17328240
Activation of PPARbeta/delta induces endothelial cell proliferation and angiogenesis.	Piqueras L, Reynolds AR, Hodivala-Dilke KM, Alfranca A, Redondo JM, Hatae T, Tanabe T, Warner TD, Bishop-Bailey D.	Arterioscler Thromb Vasc Biol. 2007	PMID:17068288
Establishment of a functional ovine fetoplacental artery endothelial cell line with a prolonged life span.	Song Y, Zheng J.	Biol Reprod. 2007	PMID:17005940 | PMCID:PMC2711505
Establishment and characterization of SV40 large T antigen-immortalized cell lines derived from fetal bovine brain tissues after prolonged cryopreservation.	Takenouchi T, Iwamaru Y, Sato M, Yokoyama T, Shinagawa M, Kitani H.	Cell Biol Int. 2007	PMID:17049468
Direct comparison of human mesenchymal stem cells derived from adipose tissues and bone marrow in mediating neovascularization in response to vascular ischemia.	Kim Y, Kim H, Cho H, Bae Y, Suh K, Jung J.	Cell Physiol Biochem. 2007	PMID:17982269
Plasticity of human adipose stem cells to perform adipogenic and endothelial differentiation.	Wosnitza M, Hemmrich K, Groger A, GrÃ¤ber S, Pallua N.	Differentiation. 2007	PMID:17244018
Bridging extended nerve defects with an artifcial nerve graft containing Schwann cells pre-seeded on polyglactin filaments.	Lohmeyer JA, Shen ZL, Walter GF, Berger A.	Int J Artif Organs. 2007	PMID:17295194
Antiangiogenic effect of bile acid acylated heparin derivative.	Park K, Kim YS, Lee GY, Nam JO, Lee SK, Park RW, Kim SY, Kim IS, Byun Y.	Pharm Res. 2007	PMID:17109210
The release of nitric oxide from S-nitrosothiols promotes angiogenesis.	Al-Ani B, Hewett PW, Ahmed S, Cudmore M, Fujisawa T, Ahmad S, Ahmed A.	PLoS One. 2006	PMID:17183652 | PMCID:PMC1762402
Low molecular weight fucoidan increases VEGF165-induced endothelial cell migration by enhancing VEGF165 binding to VEGFR-2 and NRP1.	Lake AC, Vassy R, Di Benedetto M, Lavigne D, Le Visage C, Perret GY, Letourneur D.	J Biol Chem. 2006	PMID:17028197
An oriental herbal cocktail, ka-mi-kae-kyuk-tang, exerts anti-cancer activities by targeting angiogenesis, apoptosis and metastasis.	Lee HJ, Lee EO, Rhee YH, Ahn KS, Li GX, Jiang C, LÃ¼ J, Kim SH.	Carcinogenesis. 2006	PMID:16777983
Identification of ABR-215050 as lead second generation quinoline-3-carboxamide anti-angiogenic agent for the treatment of prostate cancer.	Isaacs JT, Pili R, Qian DZ, Dalrymple SL, Garrison JB, Kyprianou N, BjÃ¶rk A, Olsson A, Leanderson T.	Prostate. 2006	PMID:16955399
Inhibition of gastric cancer cells associated angiogenesis by 15d-prostaglandin J2 through the downregulation of angiopoietin-1.	Fu YG, Sung JJ, Wu KC, Bai AH, Chan MC, Yu J, Fan DM, Leung WK.	Cancer Lett. 2006	PMID:16412567
Aminopeptidase N (APN/CD13) is selectively expressed in vascular endothelial cells and plays multiple roles in angiogenesis.	Fukasawa K, Fujii H, Saitoh Y, Koizumi K, Aozuka Y, Sekine K, Yamada M, Saiki I, Nishikawa K.	Cancer Lett. 2006	PMID:16466852
Antiangiogenesis response of endothelial cells to the antitumour drug 10-methoxy-9-nitrocamptothecin.	Yang X, Luo P, Yang B, He Q.	Pharmacol Res. 2006	PMID:16931034
KR-31831, a new synthetic anti-ischemic agent, inhibits in vivo and in vitro angiogenesis.	Yi EY, Park SY, Song HS, Son MJ, Yi KY, Yoo SE, Kim YJ.	Exp Mol Med. 2006	PMID:17079866
Malignant hematopoietic cells induce an increased expression of VEGFR-1 and VEGFR-3 on bone marrow endothelial cells via AKT and mTOR signalling pathways.	Mirshahi P, Toprak SK, Faussat AM, Dubrulle S, Marie JP, Soria C, Soria J, Mirshahi M.	Biochem Biophys Res Commun. 2006	PMID:16959214
Potent inhibition of Lewis lung cancer growth by heyneanol A from the roots of Vitis amurensis through apoptotic and anti-angiogenic activities.	Lee EO, Lee HJ, Hwang HS, Ahn KS, Chae C, Kang KS, Lu J, Kim SH.	Carcinogenesis. 2006	PMID:16675471

[Effects of triptolide and TNF-alpha on the expression of VEGF in Raji cells and on angiogenesis in ECV304 cells].	Cui GH, Chen WH, Xue KY, Liu F, Chen Y.	Zhongguo Shi Yan Xue Ye Xue Za Zhi. 2006	PMID:17096908
Neuropeptide Y induces migration, proliferation, and tube formation of endothelial cells bimodally via Y1, Y2, and Y5 receptors.	Movafagh S, Hobson JP, Spiegel S, Kleinman HK, Zukowska Z.	FASEB J. 2006	PMID:16891622
The angiosuppressive effects of 20(R)- ginsenoside Rg3.	Yue PY, Wong DY, Wu PK, Leung PY, Mak NK, Yeung HW, Liu L, Cai Z, Jiang ZH, Fan TP, Wong RN.	Biochem Pharmacol. 2006	PMID:16793023
Inhibition of angiogenesis by interleukin-4 gene therapy in rat adjuvant-induced arthritis.	Haas CS, Amin MA, Allen BB, Ruth JH, Haines GK 3rd, Woods JM, Koch AE.	Arthritis Rheum. 2006	PMID:16869003
Circulating endothelial progenitor cells from healthy smokers exhibit impaired functional activities.	Michaud SE, Dussault S, Haddad P, Groleau J, Rivard A.	Atherosclerosis. 2006	PMID:16288934
VEGF-A regulates the expression of VEGF-C in human retinal pigment epithelial cells.	Zhao B, Ma A, Cai J, Boulton M.	Br J Ophthalmol. 2006	PMID:16687456 | PMCID:PMC1857212
Antisense to cyclin D1 inhibits vascular endothelial growth factor-stimulated growth of vascular endothelial cells: implication of tumor vascularization.	Yasui M, Yamamoto H, Ngan CY, Damdinsuren B, Sugita Y, Fukunaga H, Gu J, Maeda M, Takemasa I, Ikeda M, Fujio Y, Sekimoto M, Matsuura N, Weinstein IB, Monden M.	Clin Cancer Res. 2006	PMID:16899623
Benfotiamine counteracts glucose toxicity effects on endothelial progenitor cell differentiation via Akt/FoxO signaling.	Marchetti V, Menghini R, Rizza S, Vivanti A, Feccia T, Lauro D, Fukamizu A, Lauro R, Federici M.	Diabetes. 2006	PMID:16873685
Erythropoietin improves myocardial performance in doxorubicin-induced cardiomyopathy.	Hamed S, Barshack I, Luboshits G, Wexler D, Deutsch V, Keren G, George J.	Eur Heart J. 2006	PMID:16731534
Identification of HIV-1 Tat peptides for future therapeutic angiogenesis.	Ismail M, Henklein P, Huang X, Braumann C, RÃ¼ckert RI, Dubiel W.	Eur J Haematol. 2006	PMID:16800839
Title	Description	ShortDetails	Identifiers
Cigarette smoke exposure impairs VEGF-induced endothelial cell migration: role of NO and reactive oxygen species.	Michaud SE, Dussault S, Groleau J, Haddad P, Rivard A.	J Mol Cell Cardiol. 2006	PMID:16806264
Biological and molecular characterization of a canine hemangiosarcoma-derived cell line.	Thamm DH, Dickerson EB, Akhtar N, Lewis R, Auerbach R, Helfand SC, MacEwen EG.	Res Vet Sci. 2006	PMID:16256156
[Involvement of AKT/eNOS in brain derived neurotrophic factor-induced angiogenesis].	Wang YD, Hu Y, Sun CY, He WJ, Zhang XP.	Zhonghua Xue Ye Xue Za Zhi. 2006	PMID:17172126
Negative regulation of endothelial morphogenesis and angiogenesis by S1P2 receptor.	Inoki I, Takuwa N, Sugimoto N, Yoshioka K, Takata S, Kaneko S, Takuwa Y.	Biochem Biophys Res Commun. 2006	PMID:16756949
Alphastatin downregulates vascular endothelial cells sphingosine kinase activity and suppresses tumor growth in nude mice bearing human gastric cancer xenografts.	Chen L, Li T, Li R, Wei B, Peng Z.	World J Gastroenterol. 2006	PMID:16830360
Involvement of endothelial CD44 during in vivo angiogenesis.	Cao G, Savani RC, Fehrenbach M, Lyons C, Zhang L, Coukos G, Delisser HM.	Am J Pathol. 2006	PMID:16816384 | PMCID:PMC1698758
Grateloupia longifolia polysaccharide inhibits angiogenesis by downregulating tissue factor expression in HMEC-1 endothelial cells.	Zhang C, Yang F, Zhang XW, Wang SC, Li MH, Lin LP, Ding J.	Br J Pharmacol. 2006	PMID:16715123 | PMCID:PMC1617078
Calpain-2 regulation of VEGF-mediated angiogenesis.	Su Y, Cui Z, Li Z, Block ER.	FASEB J. 2006	PMID:16816119
Anti-angiogenic action of plasma hyaluronan binding protein in human umbilical vein endothelial cells.	Jeon JW, Song HS, Moon EJ, Park SY, Son MJ, Jung SY, Kim JT, Nam DH, Choi-Miura NH, Kim KW, Kim YJ.	Int J Oncol. 2006	PMID:16773202
Blockade of angiogenesis by small molecule antagonists to protease-activated receptor-1: association with endothelial cell growth suppression and induction of apoptosis.	Zania P, Kritikou S, Flordellis CS, Maragoudakis ME, Tsopanoglou NE.	J Pharmacol Exp Ther. 2006	PMID:16595737
Inhibition of in vivo angiogenesis by N-beta-alanyl-5-S-glutathionyl-3,4-dihydroxyphenylalanine.	Nishikawa T, Akiyama N, Kunimasa K, Oikawa T, Ishizuka M, Tsujimoto M, Natori S.	Eur J Pharmacol. 2006	PMID:16725138
Modulation of angiogenic processes in cultured endothelial cells by low density lipoproteins subfractions from patients with familial hypercholesterolemia.	Tai MH, Kuo SM, Liang HT, Chiou KR, Lam HC, Hsu CM, Pownall HJ, Chen HH, Huang MT, Yang CY.	Atherosclerosis. 2006	PMID:16185697
Emodin inhibits vascular endothelial growth factor-A-induced angiogenesis by blocking receptor-2 (KDR/Flk-1) phosphorylation.	Kwak HJ, Park MJ, Park CM, Moon SI, Yoo DH, Lee HC, Lee SH, Kim MS, Lee HW, Shin WS, Park IC, Rhee CH, Hong SI.	Int J Cancer. 2006	PMID:16388516
Splenic endothelial cell lines support development of dendritic cells from bone marrow.	Despars G, O'Neill HC.	Stem Cells. 2006	PMID:16769761
Relationship between prostaglandin E2 and vascular endothelial growth factor (VEGF) in	Tamura K, Sakurai T, Kogo H.	Vascul Pharmacol. 2006	PMID:16651031

angiogenesis in human vascular endothelial cells.			
ACTIBIND, an actin-binding fungal T2-RNase with antiangiogenic and anticarcinogenic characteristics.	Roiz L, Smirnoff P, Bar-Eli M, Schwartz B, Shoseyov O.	Cancer. 2006	PMID:16586499
Cyclooxygenase-2 overexpression in human basal cell carcinoma cell line increases antiapoptosis, angiogenesis, and tumorigenesis.	Tjiu JW, Liao YH, Lin SJ, Huang YL, Tsai WL, Chu CY, Kuo ML, Jee SH.	J Invest Dermatol. 2006	PMID:16528365
Pericytes from human non-small cell lung carcinomas: an attractive target for anti-angiogenic therapy.	Bagley RG, Rouleau C, Morgenbesser SD, Weber W, Cook BP, Shankara S, Madden SL, Teicher BA.	Microvasc Res. 2006	PMID:16624341
Effects of 15d-PGJ(2) on VEGF-induced angiogenic activities and expression of VEGF receptors in endothelial cells.	Funovics P, Brostjan C, Nigisch A, Fila A, Grochot A, Mleczko K, Was H, Weigel G, Dulak J, Jozkowicz A.	Prostaglandins Other Lipid Mediat. 2006	PMID:16647637 | PMCID:PMC1463995
Subtractive transcriptomics: establishing polarity drives in vitro human endothelial morphogenesis.	Glesne DA, Zhang W, Mandava S, Ursos L, Buell ME, Makowski L, Rodi DJ.	Cancer Res. 2006	PMID:16618722
Intracellular proteolytic activity of cathepsin B is associated with capillary-like tube formation by endothelial cells in vitro.	Premzl A, Turk V, Kos J.	J Cell Biochem. 2006	PMID:16315320
Signal transducer and activator of transcription 1 activation in endothelial cells is a negative regulator of angiogenesis.	Battle TE, Lynch RA, Frank DA.	Cancer Res. 2006	PMID:16585190
Adipokine resistin promotes in vitro angiogenesis of human endothelial cells.	Mu H, Ohashi R, Yan S, Chai H, Yang H, Lin P, Yao Q, Chen C.	Cardiovasc Res. 2006	PMID:16515776
Thymosin beta10 inhibits cell migration and capillary-like tube formation of human coronary artery endothelial cells.	Mu H, Ohashi R, Yang H, Wang X, Li M, Lin P, Yao Q, Chen C.	Cell Motil Cytoskeleton. 2006	PMID:16496302
Vasohibin is up-regulated by VEGF in the retina and suppresses VEGF receptor 2 and retinal neovascularization.	Shen J, Yang X, Xiao WH, Hackett SF, Sato Y, Campochiaro PA.	FASEB J. 2006	PMID:16473886
Interleukin-20 promotes angiogenesis in a direct and indirect manner.	Hsieh MY, Chen WY, Jiang MJ, Cheng BC, Huang TY, Chang MS.	Genes Immun. 2006	PMID:16511554
Osteoblast-conditioned media stimulate membrane vesicle shedding in prostate cancer cells.	Millimaggi D, Festuccia C, Angelucci A, D'Ascenzo S, Rucci N, Flati S, Bologna M, Teti A, Pavan A, Dolo V.	Int J Oncol. 2006	PMID:16525640
IP-10 blocks vascular endothelial growth factor-induced endothelial cell motility and tube formation via inhibition of calpain.	Bodnar RJ, Yates CC, Wells A.	Circ Res. 2006	PMID:16484616
Antiangiogenic concentrations of vinflunine increase the interphase microtubule dynamics and decrease the motility of endothelial cells.	Pourroy B, HonorÃ© S, Pasquier E, Bourgarel-Rey V, Kruczynski A, Briand C, Braguer D.	Cancer Res. 2006	PMID:16540678
Reduced angiogenic responses in adult Endoglin heterozygous mice.	Jerkic M, RodrÃguez-Barbero A, Prieto M, Toporsian M, Pericacho M, Rivas-Elena JV, Obreo J, Wang A, PÃ©rez-Barriocanal F, ArÃ©valo M, BernabÃ©u C, Letarte M, LÃ³pez-Novoa JM.	Cardiovasc Res. 2006	PMID:16405930
Human CC chemokine CCL23 enhances expression of matrix metalloproteinase-2 and invasion of vascular endothelial cells.	Son KN, Hwang J, Kwon BS, Kim J.	Biochem Biophys Res Commun. 2006	PMID:16378600
Targeting tumor angiogenesis with histone deacetylase inhibitors: the hydroxamic acid derivative LBH589.	Qian DZ, Kato Y, Shabbeer S, Wei Y, Verheul HM, Salumbides B, Sanni T, Atadja P, Pili R.	Clin Cancer Res. 2006	PMID:16428510
Fibroblast growth factor-2 is a downstream mediator of phosphatidylinositol 3-kinase-Akt signaling in 14,15-epoxyeicosatrienoic acid-induced angiogenesis.	Zhang B, Cao H, Rao GN.	J Biol Chem. 2006	PMID:16286479
Sulforaphane induces inhibition of human umbilical vein endothelial cells proliferation by apoptosis.	Asakage M, Tsuno NH, Kitayama J, Tsuchiya T, Yoneyama S, Yamada J, Okaji Y, Kaisaki S, Osada T, Takahashi K, Nagawa H.	Angiogenesis. 2006	PMID:16821112
Acute-phase serum amyloid A stimulation of angiogenesis, leukocyte recruitment, and matrix degradation in rheumatoid arthritis through an NF-kappaB-dependent signal transduction pathway.	Mullan RH, Bresnihan B, Golden-Mason L, Markham T, O'Hara R, FitzGerald O, Veale DJ, Fearon U.	Arthritis Rheum. 2006	PMID:16385502
Hypoxia increases VEGF-A production by prostate cancer and bone marrow stromal cells and initiates paracrine activation of bone marrow endothelial cells.	Muir C, Chung LW, Carson DD, Farach-Carson MC.	Clin Exp Metastasis. 2006	PMID:16826426
Inhibition of tumor angiogenesis by Brahma Rasayana (BR).	Thangapazham RL, Sharma A, Gaddipati JP, Singh AK, Maheshwari RK.	J Exp Ther Oncol. 2006	PMID:17228520
Effects of TNF-alpha and curcumin on the expression of VEGF in Raji and U937 cells and on	Chen WH, Chen Y, Cui GH.	Chin Med J (Engl). 2005	PMID:16438902

angiogenesis in ECV304 cells.			
Hyperforin, a bio-active compound of St. John's Wort, is a new inhibitor of angiogenesis targeting several key steps of the process.	MartÃnez-Poveda B, Quesada AR, Medina MA.	Int J Cancer. 2005	PMID:15981212
[Inhibitory effects of Scutellaria barbatae D. Don on tumor angiogenesis and its mechanism].	Zhang NN, Bu P, Zhu HH, Shen WG.	Ai Zheng. 2005	PMID:16351792
Cytochrome P450 epoxygenases 2C8 and 2C9 are implicated in hypoxia-induced endothelial cell migration and angiogenesis.	Michaelis UR, Fisslthaler B, Barbosa-Sicard E, Falck JR, Fleming I, Busse R.	J Cell Sci. 2005	PMID:16291720
Anti-angiogenic effects of a nutrient mixture on human umbilical vein endothelial cells.	Roomi MW, Ivanov V, Kalinovsky T, Niedzwiecki A, Rath M.	Oncol Rep. 2005	PMID:16273231
[Arachidonic acid epoxygenases and their metabolites promote angiogenesis].	Wang Y, Wang JN, Liu ZJ, Wei X, Xiao X, Wang DW.	Zhonghua Xin Xue Guan Bing Za Zhi. 2005	PMID:16563285
PI3-kinase activation by GM-CSF in endothelium is upstream of Jak/Stat pathway: role of alphaGMR.	Dhar-Mascareno M, Pedraza A, Golde DW.	Biochem Biophys Res Commun. 2005	PMID:16202975
Hypoxia of endothelial cells leads to MMP-2-dependent survival and death.	Ben-Yosef Y, Miller A, Shapiro S, Lahat N.	Am J Physiol Cell Physiol. 2005	PMID:16210427
Leptin receptor and functional effects of leptin in human endothelial progenitor cells.	Wolk R, Deb A, Caplice NM, Somers VK.	Atherosclerosis. 2005	PMID:15950978
Bone morphogenetic protein 2 (BMP-2) and induction of tumor angiogenesis.	Raida M, Clement JH, Leek RD, Ameri K, Bicknell R, Niederwieser D, Harris AL.	J Cancer Res Clin Oncol. 2005	PMID:16136355
[6]-Gingerol, a pungent ingredient of ginger, inhibits angiogenesis in vitro and in vivo.	Kim EC, Min JK, Kim TY, Lee SJ, Yang HO, Han S, Kim YM, Kwon YG.	Biochem Biophys Res Commun. 2005	PMID:16081047
Calcitonin stimulates multiple stages of angiogenesis by directly acting on endothelial cells.	Chigurupati S, Kulkarni T, Thomas S, Shah G.	Cancer Res. 2005	PMID:16166333
Inhibition of the phosphatidylinositol 3-kinase/Akt pathway by inositol pentakisphosphate results in antiangiogenic and antitumor effects.	Maffucci T, Piccolo E, Cumashi A, Iezzi M, Riley AM, Saiardi A, Godage HY, Rossi C, Broggini M, Iacobelli S, Potter BV, Innocenti P, Falasca M.	Cancer Res. 2005	PMID:16166311
Title	Description	ShortDetails	Identifiers
Docosahexaenoic acid induces apoptosis in proliferating human endothelial cells.	Kim HJ, Vosseler CA, Weber PC, Erl W.	J Cell Physiol. 2005	PMID:15795939
15(S)-hydroxyeicosatetraenoic acid induces angiogenesis via activation of PI3K-Akt-mTOR-S6K1 signaling.	Zhang B, Cao H, Rao GN.	Cancer Res. 2005	PMID:16103079
C-reactive protein decreases expression of VEGF receptors and neuropilins and inhibits VEGF165-induced cell proliferation in human endothelial cells.	Yang H, Nan B, Yan S, Li M, Yao Q, Chen C.	Biochem Biophys Res Commun. 2005	PMID:15975559
Penta-O-galloyl-beta-D-glucose suppresses tumor growth via inhibition of angiogenesis and stimulation of apoptosis: roles of cyclooxygenase-2 and mitogen-activated protein kinase pathways.	Huh JE, Lee EO, Kim MS, Kang KS, Kim CH, Cha BC, Surh YJ, Kim SH.	Carcinogenesis. 2005	PMID:15845650
Arachidonic acid epoxygenase metabolites stimulate endothelial cell growth and angiogenesis via mitogen-activated protein kinase and phosphatidylinositol 3-kinase/Akt signaling pathways.	Wang Y, Wei X, Xiao X, Hui R, Card JW, Carey MA, Wang DW, Zeldin DC.	J Pharmacol Exp Ther. 2005	PMID:15840765
Peptides derived from human decorin leucine-rich repeat 5 inhibit angiogenesis.	Sulochana KN, Fan H, Jois S, Subramanian V, Sun F, Kini RM, Ge R.	J Biol Chem. 2005	PMID:15923192
Proapoptotic, antimigratory, antiproliferative, and antiangiogenic effects of commercial C-reactive protein on various human endothelial cell types in vitro: implications of contaminating presence of sodium azide in commercial preparation.	Liu C, Wang S, Deb A, Nath KA, Katusic ZS, McConnell JP, Caplice NM.	Circ Res. 2005	PMID:15976313
Tumor necrosis factor and vascular endothelial growth factor induce endothelial integrin repertories, regulating endovascular differentiation and apoptosis in a human extravillous trophoblast cell line.	Fukushima K, Miyamoto S, Tsukimori K, Kobayashi H, Seki H, Takeda S, Kensuke E, Ohtani K, Shibuya M, Nakano H.	Biol Reprod. 2005	PMID:15788755
YC-1 [3-(5'-hydroxymethyl-2'-furyl)-1-benzyl indazole] inhibits endothelial cell functions induced by angiogenic factors in vitro and angiogenesis in vivo models.	Pan SL, Guh JH, Peng CY, Wang SW, Chang YL, Cheng FC, Chang JH, Kuo SC, Lee FY, Teng CM.	J Pharmacol Exp Ther. 2005	PMID:15784655
CXCL16 is a novel angiogenic factor for human umbilical vein endothelial cells.	Zhuge X, Murayama T, Arai H, Yamauchi R, Tanaka M, Shimaoka T, Yonehara S, Kume N, Yokode M, Kita T.	Biochem Biophys Res Commun.	PMID:15883016

		2005	
Characterization of heparin affin regulatory peptide signaling in human endothelial cells.	Polykratis A, Katsoris P, Courty J, Papadimitriou E.	J Biol Chem. 2005	PMID:15797857
Effective angiostatic treatment in a murine metastatic and orthotopic hepatoma model.	Raskopf E, Dzienisowicz C, Hilbert T, Rabe C, Leifeld L, Wernert N, Sauerbruch T, Prieto J, Qian C, Caselmann WH, Schmitz V.	Hepatology. 2005	PMID:15915456
Anti-angiogenic and anti-tumor apoptotic activities of a topoisomerase II inhibiting agent SJ-8026.	Yi EY, Jeong EJ, Kang DW, Joo JH, Kwon HS, Lee SH, Park SK, Chung SG, Cho EH, Kim YJ.	Int J Oncol. 2005	PMID:15870877
Wilfoside K1N isolated from Cynanchum wilfordii inhibits angiogenesis and tumor cell invasion.	Kim MS, Baek JH, Park JA, Hwang BY, Kim SE, Lee JJ, Kim KW.	Int J Oncol. 2005	PMID:15870866
Anti-angiogenic activity of resveratrol, a natural compound from medicinal plants.	Cao Y, Fu ZD, Wang F, Liu HY, Han R.	J Asian Nat Prod Res. 2005	PMID:15621628
Combination treatment significantly enhances the efficacy of antitumor therapy by preferentially targeting angiogenesis.	Kumar P, Benedict R, Urzua F, Fischbach C, Mooney D, Polverini P.	Lab Invest. 2005	PMID:15864318
Regulation of tumor angiogenesis by fastatin, the fourth FAS1 domain of betaig-h3, via alphavbeta3 integrin.	Nam JO, Jeong HW, Lee BH, Park RW, Kim IS.	Cancer Res. 2005	PMID:15899806
Study for anti-angiogenic activities of polysaccharides isolated from Antrodia cinnamomea in endothelial cells.	Cheng JJ, Huang NK, Chang TT, Wang DL, Lu MK.	Life Sci. 2005	PMID:15850596
Antiangiogenic activity of 11,11'-dideoxyverticillin, a natural product isolated from the fungus Shiraia bambusicola.	Chen Y, Zhang YX, Li MH, Zhao WM, Shi YH, Miao ZH, Zhang XW, Lin LP, Ding J.	Biochem Biophys Res Commun. 2005	PMID:15766573
Thrombospondin-1 mimetic peptide inhibitors of angiogenesis and tumor growth: design, synthesis, and optimization of pharmacokinetics and biological activities.	Haviv F, Bradley MF, Kalvin DM, Schneider AJ, Davidson DJ, Majest SM, McKay LM, Haskell CJ, Bell RL, Nguyen B, Marsh KC, Surber BW, Uchic JT, Ferrero J, Wang YC, Leal J, Record RD, Hodde J, Badylak SF, Lesniewski RR, Henkin J.	J Med Chem. 2005	PMID:15828822
Antiangiogenic activity of beta-eudesmol in vitro and in vivo.	Tsuneki H, Ma EL, Kobayashi S, Sekizaki N, Maekawa K, Sasaoka T, Wang MW, Kimura I.	Eur J Pharmacol. 2005	PMID:15840394
Evidence of human thrombomodulin domain as a novel angiogenic factor.	Shi CS, Shi GY, Chang YS, Han HS, Kuo CH, Liu C, Huang HC, Chang YJ, Chen PS, Wu HL.	Circulation. 2005	PMID:15795324
Anti-angiogenic properties of plaunotol.	Kawai K, Tsuno NH, Kitayama J, Okaji Y, Yazawa K, Asakage M, Yamashita H, Watanabe T, Takahashi K, Nagawa H.	Anticancer Drugs. 2005	PMID:15746576
3,3'-Diindolylmethane inhibits angiogenesis and the growth of transplantable human breast carcinoma in athymic mice.	Chang X, Tou JC, Hong C, Kim HA, Riby JE, Firestone GL, Bjeldanes LF.	Carcinogenesis. 2005	PMID:15661811
Antitumor effects of in vivo caveolin gene delivery are associated with the inhibition of the proangiogenic and vasodilatory effects of nitric oxide.	Brouet A, DeWever J, Martinive P, Havaux X, Bouzin C, Sonveaux P, Feron O.	FASEB J. 2005	PMID:15623570
A natural compound (ginsenoside Re) isolated from Panax ginseng as a novel angiogenic agent for tissue regeneration.	Huang YC, Chen CT, Chen SC, Lai PH, Liang HC, Chang Y, Yu LC, Sung HW.	Pharm Res. 2005	PMID:15846472
KSHV-GPCR and CXCR2 transforming capacity and angiogenic responses are mediated through a JAK2-STAT3-dependent pathway.	Burger M, Hartmann T, Burger JA, Schraufstatter I.	Oncogene. 2005	PMID:15688008
Effect of an oversulfated exopolysaccharide on angiogenesis induced by fibroblast growth factor-2 or vascular endothelial growth factor in vitro.	Matou S, Colliec-Jouault S, Galy-Fauroux I, Ratiskol J, Sinquin C, Guezennec J, Fischer AM, Helley D.	Biochem Pharmacol. 2005	PMID:15710353
T-cell factor-4-dependent up-regulation of fibronectin is involved in fibroblast growth factor-2-induced tube formation by endothelial cells.	Kanda S, Miyata Y, Kanetake H.	J Cell Biochem. 2005	PMID:15578569
Use of embryonic stem cell-derived endothelial cells as a cell source to generate vessel structures in vitro.	McCloskey KE, Gilroy ME, Nerem RM.	Tissue Eng. 2005	PMID:15869428
The homeobox gene Gax inhibits angiogenesis through inhibition of nuclear factor-kappaB-dependent endothelial cell gene expression.	Patel S, Leal AD, Gorski DH.	Cancer Res. 2005	PMID:15735029
Silibinin strongly inhibits growth and survival of human endothelial cells via cell cycle arrest and downregulation of survivin, Akt and NF-kappaB: implications for angioprevention and antiangiogenic therapy.	Singh RP, Dhanalakshmi S, Agarwal C, Agarwal R.	Oncogene. 2005	PMID:15558015
[Study on the high expression of brain-derived neurotrophic factor in multiple myeloma patients	Hu Y, Sun CY, Wang YD, Wei WN, Wu T, He WJ, Zhao S.	Zhongguo Shi Yan	PMID:15748446

and its possible mechanism].		Xue Ye Xue Za Zhi. 2005	
A bone-derived mixture of TGF beta-superfamily members forms a more mature vascular network than bFGF or TGF-beta 2 in vivo.	Roedersheimer M, West J, Huffer W, Harral J, Benedict J.	Angiogenesis. 2005	PMID:16400522
Elucidation of the mechanisms underlying the angiogenic effects of ginsenoside Rg(1) in vivo and in vitro.	Yue PY, Wong DY, Ha WY, Fung MC, Mak NK, Yeung HW, Leung HW, Chan K, Liu L, Fan TP, Wong RN.	Angiogenesis. 2005	PMID:16328162
The anti-angiogenic effect of sinomenine.	Kok TW, Yue PY, Mak NK, Fan TP, Liu L, Wong RN.	Angiogenesis. 2005	PMID:16132613
Action of fenretinide (4-HPR) on ovarian cancer and endothelial cells.	Golubkov V, Garcia A, Markland FS.	Anticancer Res. 2005	PMID:15816545
Transplantation of bone marrow stromal cell-derived Schwann cells promotes axonal regeneration and functional recovery after complete transection of adult rat spinal cord.	Kamada T, Koda M, Dezawa M, Yoshinaga K, Hashimoto M, Koshizuka S, Nishio Y, Moriya H, Yamazaki M.	J Neuropathol Exp Neurol. 2005	PMID:15715083
Molecular isolation and characterization of a soluble isoform of activated leukocyte cell adhesion molecule that modulates endothelial cell function.	Ikeda K, Quertermous T.	J Biol Chem. 2004	PMID:15496415
Matrix GLA protein stimulates VEGF expression through increased transforming growth factor-beta1 activity in endothelial cells.	BostrÃ¶m K, Zebboudj AF, Yao Y, Lin TS, Torres A.	J Biol Chem. 2004	PMID:15456771
Apelin is a novel angiogenic factor in retinal endothelial cells.	Kasai A, Shintani N, Oda M, Kakuda M, Hashimoto H, Matsuda T, Hinuma S, Baba A.	Biochem Biophys Res Commun. 2004	PMID:15530405
Proteomic analysis of homocysteine inhibition of microvascular endothelial cell angiogenesis.	Shastry S, Tyagi N, Hayden MR, Tyagi SC.	Cell Mol Biol (Noisy-le-grand). 2004	PMID:15704257
GM-CSF induces expression of soluble VEGF receptor-1 from human monocytes and inhibits angiogenesis in mice.	Eubank TD, Roberts R, Galloway M, Wang Y, Cohn DE, Marsh CB.	Immunity. 2004	PMID:15589171
A novel method for isolation of endothelial cells and macrophages from murine tumors based on Ac-LDL uptake and CD16 expression.	Okaji Y, Tsuno NH, Kitayama J, Saito S, Takahashi T, Kawai K, Yazawa K, Asakage M, Tsuchiya T, Sakurai D, Tsuchiya N, Tokunaga K, Takahashi K, Nagawa H.	J Immunol Methods. 2004	PMID:15627623
Interleukin-6 induced basic fibroblast growth factor-dependent angiogenesis in basal cell carcinoma cell line via JAK/STAT3 and PI3-kinase/Akt pathways.	Jee SH, Chu CY, Chiu HC, Huang YL, Tsai WL, Liao YH, Kuo ML.	J Invest Dermatol. 2004	PMID:15610530
Isolation and characterization of conditionally immortalized mouse glomerular endothelial cell lines.	Rops AL, van der Vlag J, Jacobs CW, Dijkman HB, Lensen JF, Wijnhoven TJ, van den Heuvel LP, van Kuppevelt TH, Berden JH.	Kidney Int. 2004	PMID:15569308
Vascular endothelial growth factor- and thrombin-induced termination factor, Down syndrome critical region-1, attenuates endothelial cell proliferation and angiogenesis.	Minami T, Horiuchi K, Miura M, Abid MR, Takabe W, Noguchi N, Kohro T, Ge X, Aburatani H, Hamakubo T, Kodama T, Aird WC.	J Biol Chem. 2004	PMID:15448146
Triple combination of irradiation, chemotherapy (pemetrexed), and VEGFR inhibition (SU5416) in human endothelial and tumor cells.	Bischof M, Abdollahi A, Gong P, Stoffregen C, Lipson KE, Debus JU, Weber KJ, Huber PE.	Int J Radiat Oncol Biol Phys. 2004	PMID:15519795
Anti-angiogenic activity of inositol hexaphosphate (IP6).	Vucenik I, Passaniti A, Vitolo MI, Tantivejkul K, Eggleton P, Shamsuddin AM.	Carcinogenesis. 2004	PMID:15297368
Inhibition of matrix metalloproteinase-9 reduces in vitro invasion and angiogenesis in human microvascular endothelial cells.	Jadhav U, Chigurupati S, Lakka SS, Mohanam S.	Int J Oncol. 2004	PMID:15492832
Title	Description	ShortDetails	Identifiers
Dose-dependent effect of dehydroepiandrosterone, but not of its sulphate ester, on angiogenesis.	Varet J, Vincent L, Akwa Y, Mirshahi P, Lahary A, Legrand E, Opolon P, Mishal Z, Baulieu EE, Soria J, Soria C, Li H.	Eur J Pharmacol. 2004	PMID:15464086
Antitumor activities of a novel indolin-2-ketone compound, Z24: more potent inhibition on bFGF-induced angiogenesis and bcl-2 over-expressing cancer cells.	Wang LL, Li JJ, Zheng ZB, Liu HY, Du GJ, Li S.	Eur J Pharmacol. 2004	PMID:15464084
Cigarette smoke extract inhibits angiogenesis of pulmonary artery endothelial cells: the role of calpain.	Su Y, Cao W, Han Z, Block ER.	Am J Physiol Lung Cell Mol Physiol. 2004	PMID:15180919
Cytoskeletal rearrangement and caspase activation in sphingosine 1-phosphate-induced lung capillary tube formation.	Linz-McGillem LA, Moitra J, Garcia JG.	Stem Cells Dev. 2004	PMID:15588507
Pseudolarix acid B inhibits angiogenesis by antagonizing the vascular endothelial growth factor-mediated anti-apoptotic effect.	Tan WF, Zhang XW, Li MH, Yue JM, Chen Y, Lin LP, Ding J.	Eur J Pharmacol. 2004	PMID:15381043

The histone deacetylase inhibitor NVP-LAQ824 inhibits angiogenesis and has a greater antitumor effect in combination with the vascular endothelial growth factor receptor tyrosine kinase inhibitor PTK787/ZK222584.	Qian DZ, Wang X, Kachhap SK, Kato Y, Wei Y, Zhang L, Atadja P, Pili R.	Cancer Res. 2004	PMID:15374977
Hedgehog-interacting protein is highly expressed in endothelial cells but down-regulated during angiogenesis and in several human tumors.	Olsen CL, Hsu PP, Glienke J, Rubanyi GM, Brooks AR.	BMC Cancer. 2004	PMID:15294024 | PMCID:PMC512291
Simvastatin suppresses coronary artery endothelial tube formation by disrupting Ras/Raf/ERK signaling.	Miura S, Matsuo Y, Saku K.	Atherosclerosis. 2004	PMID:15262179
Interaction of cortactin and Arp2/3 complex is required for sphingosine-1-phosphate-induced endothelial cell remodeling.	Li Y, Uruno T, Haudenschild C, Dudek SM, Garcia JG, Zhan X.	Exp Cell Res. 2004	PMID:15242766
Anti-angiogenic and anti-tumor apoptotic activities of SJ-8002, a new piperazine derivative.	Yi EY, Jeong EJ, Song HS, Lee MS, Kang DW, Joo JH, Kwon HS, Lee SH, Park SK, Chung SG, Cho EH, Kim YJ.	Int J Oncol. 2004	PMID:15254733
3-Hydroxy-3-methylglutaryl-coenzyme A reductase inhibitor (pravastatin) inhibits endothelial cell proliferation dependent on G1 cell cycle arrest.	Asakage M, Tsuno NH, Kitayama J, Kawai K, Okaji Y, Yazawa K, Kaisaki S, Takahashi K, Nagawa H.	Anticancer Drugs. 2004	PMID:15205608
Gabexate mesilate inhibits colon cancer growth, invasion, and metastasis by reducing matrix metalloproteinases and angiogenesis.	Yoon WH, Jung YJ, Kim TD, Li G, Park BJ, Kim JY, Lee YC, Kim JM, Park JI, Park HD, No ZS, Lim K, Hwang BD, Kim YS.	Clin Cancer Res. 2004	PMID:15240544
Interleukin-6 increases vascular endothelial growth factor and angiogenesis in gastric carcinoma.	Huang SP, Wu MS, Shun CT, Wang HP, Lin MT, Kuo ML, Lin JT.	J Biomed Sci. 2004	PMID:15153787
Desmoplakin is required for microvascular tube formation in culture.	Zhou X, Stuart A, Dettin LE, Rodriguez G, Hoel B, Gallicano GI.	J Cell Sci. 2004	PMID:15190119
Mucosal angiogenesis regulation by CXCR4 and its ligand CXCL12 expressed by human intestinal microvascular endothelial cells.	Heidemann J, Ogawa H, Rafiee P, LÃ¼gering N, Maaser C, Domschke W, Binion DG, Dwinell MB.	Am J Physiol Gastrointest Liver Physiol. 2004	PMID:14764445
Maintenance of G1 checkpoint controls in telomerase-immortalized endothelial cells.	Freedman DA, Folkman J.	Cell Cycle. 2004	PMID:15118409
Anti-angiogenic action of the C-terminal domain of tenomodulin that shares homology with chondromodulin-I.	Oshima Y, Sato K, Tashiro F, Miyazaki J, Nishida K, Hiraki Y, Tano Y, Shukunami C.	J Cell Sci. 2004	PMID:15150318
Species-specific urokinase receptor ligands reduce glioma growth and increase survival primarily by an antiangiogenesis mechanism.	Bu X, Khankaldyyan V, Gonzales-Gomez I, Groshen S, Ye W, Zhuo S, Pons J, Stratton JR, Rosenberg S, Laug WE.	Lab Invest. 2004	PMID:15094713
Anti-proliferation effect of 3-amino-2-imino-3,4-dihydro-2H-1,3-benzothiazin-4-one (BJ-601) on human vascular endothelial cells: G0/G1 p21-associated cell cycle arrest.	Yu CH, Wu J, Su YF, Ho PY, Liang YC, Sheu MT, Lee WS.	Biochem Pharmacol. 2004	PMID:15130767
1,2,3,4,6-Penta-O-galloyl-beta-D-glucose blocks endothelial cell growth and tube formation through inhibition of VEGF binding to VEGF receptor.	Lee SJ, Lee HM, Ji ST, Lee SR, Mar W, Gho YS.	Cancer Lett. 2004	PMID:15105050
Quinoxaline 1,4-dioxides are novel angiogenesis inhibitors that potentiate antitumor effects of ionizing radiation.	Gali-Muhtasib H, Sidani M, Geara F, Mona AD, Al-Hmaira J, Haddadin MJ, Zaatari G.	Int J Oncol. 2004	PMID:15067333
An anti-VEGF ribozyme embedded within the adenoviral VAI sequence inhibits glioblastoma cell angiogenic potential in vitro.	CiafrÃ¨ SA, Niola F, Wannenes F, Farace MG.	J Vasc Res. 2004	PMID:15084786
Nucleosomes bind fibroblast growth factor-2 for increased angiogenesis in vitro and in vivo.	Tanner JE, FortÃ© A, Panchal C.	Mol Cancer Res. 2004	PMID:15192121
An anti-proliferative gene BTG1 regulates angiogenesis in vitro.	Iwai K, Hirata K, Ishida T, Takeuchi S, Hirase T, Rikitake Y, Kojima Y, Inoue N, Kawashima S, Yokoyama M.	Biochem Biophys Res Commun. 2004	PMID:15033446
Treatment of colorectal and hepatocellular carcinomas by adenoviral mediated gene transfer of endostatin and angiostatin-like molecule in mice.	Schmitz V, Wang L, Barajas M, Gomar C, Prieto J, Qian C.	Gut. 2004	PMID:15016752 | PMCID:PMC1774006
Signal transduction pathway in endothelial dysfunction.	Wilasrusmee C, Shah G, Kittur S, Halverson A, Bruch D, Kittur D.	Surg Infect (Larchmt). 2004	PMID:15142418
Adenovirus E4 gene promotes selective endothelial cell survival and angiogenesis via activation of the vascular endothelial-cadherin/Akt signaling pathway.	Zhang F, Cheng J, Hackett NR, Lam G, Shido K, Pergolizzi R, Jin DK, Crystal RG, Rafii S.	J Biol Chem. 2004	PMID:14660586
Murine endothelial cell lines as models of tumor endothelial cells.	Walter-Yohrling J, Morgenbesser S, Rouleau C, Bagley R, Callahan M, Weber W, Teicher BA.	Clin Cancer Res. 2004	PMID:15041739
Heparin affin regulatory peptide binds to vascular endothelial growth factor (VEGF) and inhibits VEGF-induced angiogenesis.	HÃ©roult M, Bernard-Pierrot I, DelbÃ© J, Hamma-Kourbali Y, Katsoris P, Barritault D, Papadimitriou E, Plouet J, Courty J.	Oncogene. 2004	PMID:15001987

Effects of adrenomedullin on endothelial cells in the multistep process of angiogenesis: involvement of CRLR/RAMP2 and CRLR/RAMP3 receptors.	Fernandez-Sauze S, Delfino C, Mabrouk K, Dussert C, Chinot O, Martin PM, Grisoli F, Ouafik L, Boudouresque F.	Int J Cancer. 2004	PMID:14712479
Bone morphogenetic protein-2 stimulates angiogenesis in developing tumors.	Langenfeld EM, Langenfeld J.	Mol Cancer Res. 2004	PMID:15037653
Valproic acid inhibits angiogenesis in vitro and in vivo.	Michaelis M, Michaelis UR, Fleming I, Suhan T, Cinatl J, Blaheta RA, Hoffmann K, Kotchetkov R, Busse R, Nau H, Cinatl J Jr.	Mol Pharmacol. 2004	PMID:14978230
Anti-angiogenic efficacy of grape seed extract in endothelial cells.	Agarwal C, Singh RP, Dhanalakshmi S, Agarwal R.	Oncol Rep. 2004	PMID:14767522
Angiomotin expression promotes hemangioendothelioma invasion.	Levchenko T, Bratt A, Arbiser JL, Holmgren L.	Oncogene. 2004	PMID:14730344
The neuropeptide secretoneurin acts as a direct angiogenic cytokine in vitro and in vivo.	Kirchmair R, Gander R, Egger M, Hanley A, Silver M, Ritsch A, Murayama T, Kaneider N, Sturm W, Kearny M, Fischer-Colbrie R, Kircher B, Gaenzer H, Wiedermann CJ, Ropper AH, Losordo DW, Patsch JR, Schratzberger P.	Circulation. 2004	PMID:14970115
An angiogenesis inhibitor E7820 shows broad-spectrum tumor growth inhibition in a xenograft model: possible value of integrin alpha2 on platelets as a biological marker.	Semba T, Funahashi Y, Ono N, Yamamoto Y, Sugi NH, Asada M, Yoshimatsu K, Wakabayashi T.	Clin Cancer Res. 2004	PMID:14977846
Prostate cancer prevention by silibinin.	Singh RP, Agarwal R.	Curr Cancer Drug Targets. 2004	PMID:14965263
Capsaicin inhibits in vitro and in vivo angiogenesis.	Min JK, Han KY, Kim EC, Kim YM, Lee SW, Kim OH, Kim KW, Gho YS, Kwon YG.	Cancer Res. 2004	PMID:14744780
Inhibition of lymphangiogenesis-related properties of murine lymphatic endothelial cells and lymph node metastasis of lung cancer by the matrix metalloproteinase inhibitor MMI270.	Nakamura ES, Koizumi K, Kobayashi M, Saiki I.	Cancer Sci. 2004	PMID:14720323
New murine model of spontaneous autologous tissue engineering, combining an arteriovenous pedicle with matrix materials.	Cronin KJ, Messina A, Knight KR, Cooper-White JJ, Stevens GW, Penington AJ, Morrison WA.	Plast Reconstr Surg. 2004	PMID:14707645
Functional characterization of sphingosine 1-phosphate receptor agonist in human endothelial cells.	Butler J, Lana D, Round O, LaMontagne K.	Prostaglandins Other Lipid Mediat. 2004	PMID:15165029
Inhibitory effect of caffeic acid phenethyl ester on angiogenesis, tumor invasion, and metastasis.	Liao HF, Chen YY, Liu JJ, Hsu ML, Shieh HJ, Liao HJ, Shieh CJ, Shiao MS, Chen YJ.	J Agric Food Chem. 2003	PMID:14690372
Troponin I peptide (Glu94-Leu123), a cartilage-derived angiogenesis inhibitor: in vitro and in vivo effects on human endothelial cells and on pancreatic cancer.	Kern BE, Balcom JH, Antoniu BA, Warshaw AL, FernÃ¡ndez-del Castillo C.	J Gastrointest Surg. 2003	PMID:14675705
An N-terminal 80 kDa recombinant fragment of human thrombospondin-2 inhibits vascular endothelial growth factor induced endothelial cell migration in vitro and tumor growth and angiogenesis in vivo.	Noh YH, Matsuda K, Hong YK, Kunstfeld R, Riccardi L, Koch M, Oura H, Dadras SS, Streit M, Detmar M.	J Invest Dermatol. 2003	PMID:14675207
Diverse origin and function of cells with endothelial phenotype obtained from adult human blood.	Gulati R, Jevremovic D, Peterson TE, Chatterjee S, Shah V, Vile RG, Simari RD.	Circ Res. 2003	PMID:14605020
A vascular endothelial growth factor high affinity receptor 1-specific peptide with antiangiogenic activity identified using a phage display peptide library.	El-Mousawi M, Tchistiakova L, Yurchenko L, Pietrzynski G, Moreno M, Stanimirovic D, Ahmad D, Alakhov V.	J Biol Chem. 2003	PMID:12954624
Heparin inhibition of endothelial cell proliferation and organization is dependent on molecular weight.	Khorana AA, Sahni A, Altland OD, Francis CW.	Arterioscler Thromb Vasc Biol. 2003	PMID:12920044
Radiation induced-tubulogenesis in endothelial cells is antagonized by the antiangiogenic properties of green tea polyphenol (-) epigallocatechin-3-gallate.	Annabi B, Lee YT, Martel C, Pilorget A, Bahary JP, BÃ©liveau R.	Cancer Biol Ther. 2003	PMID:14688468
Amifostine has antiangiogenic properties in vitro by changing the redox status of human endothelial cells.	Giannopoulou E, Papadimitriou E.	Free Radic Res. 2003	PMID:14703731
ACE inhibition actively promotes cell survival by altering gene expression.	Hamdi HK, Castellon R.	Biochem Biophys Res Commun. 2003	PMID:14559246
Title	Description	ShortDetails	Identifiers
Nuclear translocation of phosphorylated STAT3 is essential for vascular endothelial growth factor-induced human dermal microvascular endothelial cell migration and tube formation.	Yahata Y, Shirakata Y, Tokumaru S, Yamasaki K, Sayama K, Hanakawa Y, Detmar M, Hashimoto K.	J Biol Chem. 2003	PMID:12874294
Angiogenic role of adrenomedullin through activation of Akt, mitogen-activated protein kinase, and focal adhesion kinase in endothelial cells.	Kim W, Moon SO, Sung MJ, Kim SH, Lee S, So JN, Park SK.	FASEB J. 2003	PMID:12897063

Aspirin and salicylate inhibit colon cancer medium- and VEGF-induced endothelial tube formation: correlation with suppression of cyclooxygenase-2 expression.	Shtivelband MI, Juneja HS, Lee S, Wu KK.	J Thromb Haemost. 2003	PMID:14521608
Vinblastine inhibits the angiogenic response induced by adrenomedullin in vitro and in vivo.	Ribatti D, Guidolin D, Conconi MT, Nico B, Baiguera S, Parnigotto PP, Vacca A, Nussdorfer GG.	Oncogene. 2003	PMID:14508526
CEP-7055: a novel, orally active pan inhibitor of vascular endothelial growth factor receptor tyrosine kinases with potent antiangiogenic activity and antitumor efficacy in preclinical models.	Ruggeri B, Singh J, Gingrich D, Angeles T, Albom M, Yang S, Chang H, Robinson C, Hunter K, Dobrzanski P, Jones-Bolin S, Pritchard S, Aimone L, Klein-Szanto A, Herbert JM, Bono F, Schaeffer P, Casellas P, Bourie B, Pili R, Isaacs J, Ator M, et al.	Cancer Res. 2003	PMID:14522925
Endothelial precursor cells as a model of tumor endothelium: characterization and comparison with mature endothelial cells.	Bagley RG, Walter-Yohrling J, Cao X, Weber W, Simons B, Cook BP, Chartrand SD, Wang C, Madden SL, Teicher BA.	Cancer Res. 2003	PMID:14522911
Dimethylarginine dimethylaminohydrolase activity modulates ADMA levels, VEGF expression, and cell phenotype.	Smith CL, Birdsey GM, Anthony S, Arrigoni FI, Leiper JM, Vallance P.	Biochem Biophys Res Commun. 2003	PMID:12927816
Targeted disruption of endothelial cell-selective adhesion molecule inhibits angiogenic processes in vitro and in vivo.	Ishida T, Kundu RK, Yang E, Hirata K, Ho YD, Quertermous T.	J Biol Chem. 2003	PMID:12819200
Arginine deiminase: a potential inhibitor of angiogenesis and tumour growth.	Park IS, Kang SW, Shin YJ, Chae KY, Park MO, Kim MY, Wheatley DN, Min BH.	Br J Cancer. 2003	PMID:12942125 | PMCID:PMC2394481
M-CSF induces vascular endothelial growth factor production and angiogenic activity from human monocytes.	Eubank TD, Galloway M, Montague CM, Waldman WJ, Marsh CB.	J Immunol. 2003	PMID:12928417
Low molecular weight fucoidan and heparin enhance the basic fibroblast growth factor-induced tube formation of endothelial cells through heparan sulfate-dependent alpha6 overexpression.	Chabut D, Fischer AM, Colliec-Jouault S, Laurendeau I, Matou S, Le Bonniec B, Helley D.	Mol Pharmacol. 2003	PMID:12920206
[Celastrol in the inhibition of neovascularization].	Huang YL, Zhou YX, Zhou D, Xu QN, Ye M, Sun CF, Du ZW.	Zhonghua Zhong Liu Za Zhi. 2003	PMID:14575562
Migration inhibitory factor mediates angiogenesis via mitogen-activated protein kinase and phosphatidylinositol kinase.	Amin MA, Volpert OV, Woods JM, Kumar P, Harlow LA, Koch AE.	Circ Res. 2003	PMID:12881477
Glucose-6-phosphate dehydrogenase modulates vascular endothelial growth factor-mediated angiogenesis.	Leopold JA, Walker J, Scribner AW, Voetsch B, Zhang YY, Loscalzo AJ, Stanton RC, Loscalzo J.	J Biol Chem. 2003	PMID:12777375
AMP-activated protein kinase (AMPK) signaling in endothelial cells is essential for angiogenesis in response to hypoxic stress.	Nagata D, Mogi M, Walsh K.	J Biol Chem. 2003	PMID:12788940
Novel proangiogenic effect of factor XIII associated with suppression of thrombospondin 1 expression.	Dardik R, Solomon A, Loscalzo J, Eskaraev R, Bialik A, Goldberg I, Schiby G, Inbal A.	Arterioscler Thromb Vasc Biol. 2003	PMID:12805075
beta1-integrin-ligand disengagement induces in vitro capillary tube disruption mediated by p38 MAPK activity.	Da Silva MS, Siddiqui J, Halverson A, Wilasrusmee C, Bruch D, Kittur DS.	Surgery. 2003	PMID:12947314
Ras/Raf1-dependent signal in sphingosine-1-phosphate-induced tube formation in human coronary artery endothelial cells.	Miura S, Tanigawa H, Matsuo Y, Fujino M, Kawamura A, Saku K.	Biochem Biophys Res Commun. 2003	PMID:12821130
Identification of the alphavbeta3 integrin-interacting motif of betaig-h3 and its anti-angiogenic effect.	Nam JO, Kim JE, Jeong HW, Lee SJ, Lee BH, Choi JY, Park RW, Park JY, Kim IS.	J Biol Chem. 2003	PMID:12704192
SU5416 and SU6668 attenuate the angiogenic effects of radiation-induced tumor cell growth factor production and amplify the direct anti-endothelial action of radiation in vitro.	Abdollahi A, Lipson KE, Han X, Krempien R, Trinh T, Weber KJ, Hahnfeldt P, Hlatky L, Debus J, Howlett AR, Huber PE.	Cancer Res. 2003	PMID:12839971
Matrix metalloproteinase regulation of sphingosine-1-phosphate-induced angiogenic properties of bone marrow stromal cells.	Annabi B, Thibeault S, Lee YT, Bousquet-Gagnon N, Eliopoulos N, Barrette S, Galipeau J, BÃ©liveau R.	Exp Hematol. 2003	PMID:12842709
A culture system using human foreskin fibroblasts as feeder cells allows production of human embryonic stem cells.	Hovatta O, Mikkola M, Gertow K, StrÃ¶mberg AM, Inzunza J, Hreinsson J, Rozell B, Blennow E, AndÃ¤ng M, Ahrlund-Richter L.	Hum Reprod. 2003	PMID:12832363
Antiangiogenic property of human thrombin.	Chan B, Merchan JR, Kale S, Sukhatme VP.	Microvasc Res. 2003	PMID:12826069
Suppression of angiogenesis and tumor growth by adenoviral-mediated gene transfer of pigment epithelium-derived factor.	Wang L, Schmitz V, Perez-Mediavilla A, Izal I, Prieto J, Qian C.	Mol Ther. 2003	PMID:12842430
Efficacy of dendrimer-mediated angiostatin and TIMP-2 gene delivery on inhibition of tumor growth and angiogenesis: in vitro and in vivo studies.	Vincent L, Varet J, Pille JY, Bompais H, Opolon P, Maksimenko A, Malvy C, Mirshahi M, Lu H, Vannier JP,	Int J Cancer. 2003	PMID:12704680

	Soria C, Li H.		
Angiogenic actions of angiopoietin-1 require endothelium-derived nitric oxide.	Babaei S, Teichert-Kuliszewska K, Zhang Q, Jones N, Dumont DJ, Stewart DJ.	Am J Pathol. 2003	PMID:12759249 | PMCID:PMC1868142
Structural elements of kallistatin required for inhibition of angiogenesis.	Miao RQ, Chen V, Chao L, Chao J.	Am J Physiol Cell Physiol. 2003	PMID:12734113
Tetraarsenic oxide, a novel orally administrable angiogenesis inhibitor.	Park MJ, Park IC, Bae IJ, Seo KM, Lee SH, Hong SI, Eun CK, Zhang W, Rhee CH.	Int J Oncol. 2003	PMID:12738993
Antiangiogenic effect of KR-31372 by apoptosis via mediation of mitochondrial KATP channel opening and the phosphatase and tensin homolog deleted from chromosome 10 phosphorylation.	Kim KY, Shin YW, Kim SO, Lim H, Yoo SE, Hong KW.	J Pharmacol Exp Ther. 2003	PMID:12626642
Src and phosphatidylinositol 3-kinase mediate soluble E-selectin-induced angiogenesis.	Kumar P, Amin MA, Harlow LA, Polverini PJ, Koch AE.	Blood. 2003	PMID:12522014
Inhibition of endothelial cell activation by the homeobox gene Gax.	Gorski DH, Leal AJ.	J Surg Res. 2003	PMID:12842453
KR-31372 inhibits KDR/Flk-1 tyrosine phosphorylation via K+(ATP) channel opening in its antiangiogenic effect.	Kim KY, Kim SO, Lim H, Yoo SE, Hong KW.	Eur J Pharmacol. 2003	PMID:12681433
Clinical significance of aminopeptidase N/CD13 expression in human pancreatic carcinoma.	Ikeda N, Nakajima Y, Tokuhara T, Hattori N, Sho M, Kanehiro H, Miyake M.	Clin Cancer Res. 2003	PMID:12684426
Role of endothelin-1 in microvascular dysfunction caused by cyclosporin A.	Wilasrusmee C, Da Silva M, Siddiqui J, Bruch D, Kittur S, Wilasrusmee S, Kittur DS.	J Am Coll Surg. 2003	PMID:12691936
In vitro and in vivo induction of antiangiogenic activity by plasminogen activators and captopril.	Merchan JR, Chan B, Kale S, Schnipper LE, Sukhatme VP.	J Natl Cancer Inst. 2003	PMID:12618504
Tissue-specific distributions of alternatively spliced human PECAM-1 isoforms.	Wang Y, Su X, Sorenson CM, Sheibani N.	Am J Physiol Heart Circ Physiol. 2003	PMID:12433657
Oncogenic transformation induces tumor angiogenesis: a role for PAR1 activation.	Yin YJ, Salah Z, Maoz M, Even Ram SC, Ochayon S, Neufeld G, Katzav S, Bar-Shavit R.	FASEB J. 2003	PMID:12554695
Betacellulin induces angiogenesis through activation of mitogen-activated protein kinase and phosphatidylinositol 3'-kinase in endothelial cell.	Kim HS, Shin HS, Kwak HJ, Cho CH, Lee CO, Koh GY.	FASEB J. 2003	PMID:12475887
Neuropeptide Y: a novel mechanism for ischemic angiogenesis.	Zukowska Z, Grant DS, Lee EW.	Trends Cardiovasc Med. 2003	PMID:12586445
Different inhibitors of plasmin differentially affect angiostatin production and angiogenesis.	Hatziapostolou M, Katsoris P, Papadimitriou E.	Eur J Pharmacol. 2003	PMID:12535853
Epoxygenase-driven angiogenesis in human lung microvascular endothelial cells.	Medhora M, Daniels J, Mundey K, Fisslthaler B, Busse R, Jacobs ER, Harder DR.	Am J Physiol Heart Circ Physiol. 2003	PMID:12388259
Anti-angiogenic activity of contortrostatin, a disintegrin from Agkistrodon contortrix contortrix snake venom.	Golubkov V, Hawes D, Markland FS.	Angiogenesis. 2003	PMID:15041797
Alprostadil suppresses angiogenesis in vitro and in vivo in the murine Matrigel plug assay.	Cattaneo MG, Pola S, DehÃ² V, Sanguini AM, Vicentini LM.	Br J Pharmacol. 2003	PMID:12540529 | PMCID:PMC1573673
Photoconstructs of nerve guidance prosthesis using photoreactive gelatin as a scaffold.	GÃ¡mez E, Ikezaki K, Fukui M, Matsuda T.	Cell Transplant. 2003	PMID:12953922
Angiogenesis assays: a critical overview.	Auerbach R, Lewis R, Shinners B, Kubai L, Akhtar N.	Clin Chem. 2003	PMID:12507958
Trefoil peptides as proangiogenic factors in vivo and in vitro: implication of cyclooxygenase-2 and EGF receptor signaling.	Rodrigues S, Van Aken E, Van Bocxlaer S, Attoub S, Nguyen QD, Bruyneel E, Westley BR, May FE, Thim L, Mareel M, Gespach C, Emami S.	FASEB J. 2003	PMID:12522107
Hypoxia promotes murine bone-marrow-derived stromal cell migration and tube formation.	Annabi B, Lee YT, Turcotte S, Naud E, Desrosiers RR, Champagne M, Eliopoulos N, Galipeau J, BÃ©liveau R.	Stem Cells. 2003	PMID:12743328
Inhibition of human cancer cell growth and metastasis in nude mice by oral intake of modified citrus pectin.	Nangia-Makker P, Hogan V, Honjo Y, Baccarini S, Tait L, Bresalier R, Raz A.	J Natl Cancer Inst. 2002	PMID:12488479
Deficiency in endothelial nitric oxide synthase impairs myocardial angiogenesis.	Zhao X, Lu X, Feng Q.	Am J Physiol Heart Circ Physiol. 2002	PMID:12388304
A novel strategy for the generation of angiostatic kringle regions from a precursor derived from plasminogen.	Schmitz V, Wang L, Barajas M, Peng D, Prieto J, Qian C.	Gene Ther. 2002	PMID:12424612
Title	Description	ShortDetails	Identifiers
CRIM1 is involved in endothelial cell capillary formation in vitro and is expressed in blood vessels in vivo.	Glienke J, Sturz A, Menrad A, Thierauch KH.	Mech Dev. 2002	PMID:12464430

Cerebral capillary endothelial cell mitogenesis and morphogenesis induced by astrocytic epoxyeicosatrienoic Acid.	Zhang C, Harder DR.	Stroke. 2002	PMID:12468797
[Hemangioblastic characteristics of fetal bone marrow-derived Flk1+ CD34- cells].	Guo H, Zhao ZG, Liu JW, Chen HS, Zhao CH.	Zhongguo Yi Xue Ke Xue Yuan Xue Bao. 2002	PMID:12905689
Roles of cell adhesion molecules in tumor angiogenesis induced by cotransplantation of cancer and endothelial cells to nude rats.	Tei K, Kawakami-Kimura N, Taguchi O, Kumamoto K, Higashiyama S, Taniguchi N, Toda K, Kawata R, Hisa Y, Kannagi R.	Cancer Res. 2002	PMID:12414659
Combined chemo/anti-angiogenic cancer therapy against Lewis lung carcinoma (3LL) pulmonary metastases.	Datta A, Kitson RP, Xue Y, al-Atrash G, Mazar AP, Jones TR, Goldfarb RH.	In Vivo. 2002	PMID:12494889
Recombinant arginine deiminase as a potential anti-angiogenic agent.	Beloussow K, Wang L, Wu J, Ann D, Shen WC.	Cancer Lett. 2002	PMID:12065090
Histidine-proline-rich glycoprotein has potent antiangiogenic activity mediated through the histidine-proline-rich domain.	Juarez JC, Guan X, Shipulina NV, Plunkett ML, Parry GC, Shaw DE, Zhang JC, Rabbani SA, McCrae KR, Mazar AP, Morgan WT, DoÃ±ate F.	Cancer Res. 2002	PMID:12235005
FEEL-1, a novel scavenger receptor with in vitro bacteria-binding and angiogenesis-modulating activities.	Adachi H, Tsujimoto M.	J Biol Chem. 2002	PMID:12077138
Fully human antibodies to MCAM/MUC18 inhibit tumor growth and metastasis of human melanoma.	Mills L, Tellez C, Huang S, Baker C, McCarty M, Green L, Gudas JM, Feng X, Bar-Eli M.	Cancer Res. 2002	PMID:12208768
Both protein activation and gene expression are involved in early vascular tube formation in vitro.	Grove AD, Prabhu VV, Young BL, Lee FC, Kulpa V, Munson PJ, Kohn EC.	Clin Cancer Res. 2002	PMID:12231549
Modulation of radiation response and tumor-induced angiogenesis after epidermal growth factor receptor inhibition by ZD1839 (Iressa).	Huang SM, Li J, Armstrong EA, Harari PM.	Cancer Res. 2002	PMID:12154033
The antifungal drug ciclopirox inhibits deoxyhypusine and proline hydroxylation, endothelial cell growth and angiogenesis in vitro.	Clement PM, Hanauske-Abel HM, Wolff EC, Kleinman HK, Park MH.	Int J Cancer. 2002	PMID:12115536
An attempt to promote neo-vascularization by employing a newly synthesized inhibitor of protein tyrosine phosphatase.	Soeda S, Shimada T, Koyanagi S, Yokomatsu T, Murano T, Shibuya S, Shimeno H.	FEBS Lett. 2002	PMID:12135741
Multifunctional anti-angiogenic activity of the cyclic peroxide ANO-2 with antitumor activity.	Arakawa K, Endo Y, Kimura M, Yoshida T, Kitaoka T, Inakazu T, Nonami Y, Abe M, Masuyama A, Nojima M, Sasaki T.	Int J Cancer. 2002	PMID:12115573
[Anti-angiogenesis effects of aldosterone antagonist diuretics].	Guggino S, Weltin D, Chapelon D, Imbs JL, Stephan D.	Arch Mal Coeur Vaiss. 2002	PMID:12365088
Inhibition of angiogenesis in vitro by alphav integrin-directed antisense oligonucleotides.	Kronenwett R, GrÃ¤f T, Nedbal W, Weber M, Steidl U, Rohr UP, MÃ¶hler T, Haas R.	Cancer Gene Ther. 2002	PMID:12082459
Carp oil or oleic acid, but not linoleic acid or linolenic acid, inhibits tumor growth and metastasis in Lewis lung carcinoma-bearing mice.	Kimura Y.	J Nutr. 2002	PMID:12097695
Rac regulates endothelial morphogenesis and capillary assembly.	Connolly JO, Simpson N, Hewlett L, Hall A.	Mol Biol Cell. 2002	PMID:12134084 | PMCID:PMC117328
Primitive endothelial cell lines from the porcine embryonic yolk sac.	Plendl J, Gilligan BJ, Wang SJ, Lewis R, Shinners B, Vandenbroeck K, Auerbach R.	In Vitro Cell Dev Biol Anim. 2002	PMID:12513121
Eicosanoid regulation of vascular endothelial growth factor expression and angiogenesis in microvessel endothelial cells.	Mezentsev A, Seta F, Dunn MW, Ono N, Falck JR, Laniado-Schwartzman M.	J Biol Chem. 2002	PMID:11901160
Hypoxia upregulates Bcl-2 expression and suppresses interferon-gamma induced antiangiogenic activity in human tumor derived endothelial cells.	Wang JH, Wu QD, Bouchier-Hayes D, Redmond HP.	Cancer. 2002	PMID:12173346
A new in vitro model to study endothelial injury.	Wilasrusmee C, Da Silva M, Singh B, Kittur S, Siddiqui J, Bruch D, Wilasrusmee S, Kittur DS.	J Surg Res. 2002	PMID:12020132
Involvement of human PECAM-1 in angiogenesis and in vitro endothelial cell migration.	Cao G, O'Brien CD, Zhou Z, Sanders SM, Greenbaum JN, Makrigiannakis A, DeLisser HM.	Am J Physiol Cell Physiol. 2002	PMID:11940533
Green tea constituent epigallocatechin-3-gallate inhibits angiogenic differentiation of human endothelial cells.	Singh AK, Seth P, Anthony P, Husain MM, Madhavan S, Mukhtar H, Maheshwari RK.	Arch Biochem Biophys. 2002	PMID:12054484
Molecular inhibition of angiogenesis and metastatic potential in human squamous cell carcinomas after epidermal growth factor receptor blockade.	Huang SM, Li J, Harari PM.	Mol Cancer Ther. 2002	PMID:12479268
Endothelial cells derived from human embryonic stem cells.	Levenberg S, Golub JS, Amit M, Itskovitz-Eldor J, Langer R.	Proc Natl Acad Sci U S A. 2002	PMID:11917100 | PMCID:PMC123658

Combined anti-fetal liver kinase 1 monoclonal antibody and continuous low-dose doxorubicin inhibits angiogenesis and growth of human soft tissue sarcoma xenografts by induction of endothelial cell apoptosis.	Zhang L, Yu D, Hicklin DJ, Hannay JA, Ellis LM, Pollock RE.	Cancer Res. 2002	PMID:11929822
Vascular endothelial cells that express dystroglycan are involved in angiogenesis.	Hosokawa H, Ninomiya H, Kitamura Y, Fujiwara K, Masaki T.	J Cell Sci. 2002	PMID:11896196
Betulinic acid inhibits growth factor-induced in vitro angiogenesis via the modulation of mitochondrial function in endothelial cells.	Kwon HJ, Shim JS, Kim JH, Cho HY, Yum YN, Kim SH, Yu J.	Jpn J Cancer Res. 2002	PMID:11985792
TNF-related activation-induced cytokine (TRANCE) induces angiogenesis through the activation of Src and phospholipase C (PLC) in human endothelial cells.	Kim YM, Kim YM, Lee YM, Kim HS, Kim JD, Choi Y, Kim KW, Lee SY, Kwon YG.	J Biol Chem. 2002	PMID:11741951
DNAzymes to beta 1 and beta 3 mRNA down-regulate expression of the targeted integrins and inhibit endothelial cell capillary tube formation in fibrin and matrigel.	Cieslak M, Niewiarowska J, Nawrot M, Koziolkiewicz M, Stec WJ, Cierniewski CS.	J Biol Chem. 2002	PMID:11675378
Antiangiogenic activity of aeroplysinin-1, a brominated compound isolated from a marine sponge.	RodrÃguez-Nieto S, GonzÃ¡lez-Iriarte M, Carmona R, MuÃ±oz-ChÃ¡puli R, Medina MA, Quesada AR.	FASEB J. 2002	PMID:11772945
Connective tissue growth factor binds vascular endothelial growth factor (VEGF) and inhibits VEGF-induced angiogenesis.	Inoki I, Shiomi T, Hashimoto G, Enomoto H, Nakamura H, Makino K, Ikeda E, Takata S, Kobayashi K, Okada Y.	FASEB J. 2002	PMID:11744618
Angiotensinogen and its cleaved derivatives inhibit angiogenesis.	CÃ©lÃ©rier J, Cruz A, LamandÃ© N, Gasc JM, Corvol P.	Hypertension. 2002	PMID:11847188
[Cloning of a novel gene, ANGPTL4 and the functional study in angiogenesis].	Zhu H, Li J, Qin W, Yang Y, He X, Wan D, Gu J.	Zhonghua Yi Xue Za Zhi. 2002	PMID:11953136
Histone deacetylase inhibitor FK228 inhibits tumor angiogenesis.	Kwon HJ, Kim MS, Kim MJ, Nakajima H, Kim KW.	Int J Cancer. 2002	PMID:11774279
Comparative study of tube assembly in three-dimensional collagen matrix and on Matrigel coats.	MontaÃ±ez E, Casaroli-Marano RP, VilarÃ³ S, Pagan R.	Angiogenesis. 2002	PMID:12831057
Involvement of endothelial nitric oxide in sphingosine-1-phosphate-induced angiogenesis.	Rikitake Y, Hirata K, Kawashima S, Ozaki M, Takahashi T, Ogawa W, Inoue N, Yokoyama M.	Arterioscler Thromb Vasc Biol. 2002	PMID:11788469
The chemopreventive agent oltipraz possesses potent antiangiogenic activity in vitro, ex vivo, and in vivo and inhibits tumor xenograft growth.	Ruggeri BA, Robinson C, Angeles T, Wilkinson J 4th, Clapper ML.	Clin Cancer Res. 2002	PMID:11801568
Hsp90 and caveolin are key targets for the proangiogenic nitric oxide-mediated effects of statins.	Brouet A, Sonveaux P, Dessy C, Moniotte S, Balligand JL, Feron O.	Circ Res. 2001	PMID:11701613
Regulation of human cytotrophoblast morphogenesis by hepatocyte growth factor/scatter factor.	Dokras A, Gardner LM, Seftor EA, Hendrix MJ.	Biol Reprod. 2001	PMID:11566754
Fibrocytes induce an angiogenic phenotype in cultured endothelial cells and promote angiogenesis in vivo.	Hartlapp I, Abe R, Saeed RW, Peng T, Voelter W, Bucala R, Metz CN.	FASEB J. 2001	PMID:11641248
Differential involvement of the hyaluronan (HA) receptors CD44 and receptor for HA-mediated motility in endothelial cell function and angiogenesis.	Savani RC, Cao G, Pooler PM, Zaman A, Zhou Z, DeLisser HM.	J Biol Chem. 2001	PMID:11448954
Vascular endothelial growth factor receptor-1 modulates vascular endothelial growth factor-mediated angiogenesis via nitric oxide.	Bussolati B, Dunk C, Grohman M, Kontos CD, Mason J, Ahmed A.	Am J Pathol. 2001	PMID:11549592 | PMCID:PMC1850457
Evidence of IL-18 as a novel angiogenic mediator.	Park CC, Morel JC, Amin MA, Connors MA, Harlow LA, Koch AE.	J Immunol. 2001	PMID:11466388
Plasminogen activator inhibitor-1 promotes angiogenesis by stimulating endothelial cell migration toward fibronectin.	Isogai C, Laug WE, Shimada H, Declerck PJ, Stins MF, Durden DL, Erdreich-Epstein A, DeClerck YA.	Cancer Res. 2001	PMID:11454712
Identification of the anti-angiogenic site within vascular basement membrane-derived tumstatin.	Maeshima Y, Manfredi M, Reimer C, Holthaus KA, Hopfer H, Chandamuri BR, Kharbanda S, Kalluri R.	J Biol Chem. 2001	PMID:11278365
Inhibition of vascular endothelial cells by 1,4-phenylenebis (methylene)selenocyanate--a novel chemopreventive organoselenium compound.	Schumacher JJ, Upadhyaya P, Ramakrishnan S.	Anticancer Res. 2001	PMID:11497282
Rhodostomin, a snake venom disintegrin, inhibits angiogenesis elicited by basic fibroblast growth factor and suppresses tumor growth by a selective alpha(v)beta(3) blockade of endothelial cells.	Yeh CH, Peng HC, Yang RS, Huang TF.	Mol Pharmacol. 2001	PMID:11306719
Angiogenic activity of pyruvic acid in in vivo and in vitro angiogenesis models.	Lee MS, Moon EJ, Lee SW, Kim MS, Kim KW, Kim YJ.	Cancer Res. 2001	PMID:11309282
Title	Description	ShortDetails	Identifiers
HARP induces angiogenesis in vivo and in vitro: implication of N or C terminal peptides.	Papadimitriou E, Polykratis A, Courty J, Koolwijk P, Heroult M, Katsoris P.	Biochem Biophys Res Commun. 2001	PMID:11264008

Extracellular matrix-derived angiogenic factor(s) inhibit endothelial cell proliferation, enhance differentiation, and stimulate angiogenesis in vivo.	Akhta N, Carlso S, Pesarini A, Ambulos N, Passaniti A.	Endothelium. 2001	PMID:11824474
In vitro matrigel angiogenesis assays.	Ponce ML.	Methods Mol Med. 2001	PMID:21340921
The ephrin-A1 ligand and its receptor, EphA2, are expressed during tumor neovascularization.	Ogawa K, Pasqualini R, Lindberg RA, Kain R, Freeman AL, Pasquale EB.	Oncogene. 2000	PMID:11146556
Monomethyl selenium--specific inhibition of MMP-2 and VEGF expression: implications for angiogenic switch regulation.	Jiang C, Ganther H, Lu J.	Mol Carcinog. 2000	PMID:11170262
Effects of microenvironmental extracellular pH and extracellular matrix proteins on angiostatin's activity and on intracellular pH.	Wahl ML, Grant DS.	Gen Pharmacol. 2000	PMID:11888684
Expression of antisense to integrin subunit beta 3 inhibits microvascular endothelial cell capillary tube formation in fibrin.	Dallabrida SM, De Sousa MA, Farrell DH.	J Biol Chem. 2000	PMID:10922359
Antiangiogenesis efficacy of nitric oxide donors.	Powell JA, Mohamed SN, Kerr JS, Mousa SA.	J Cell Biochem. 2000	PMID:11029757
Anti-angiogenic potential of a cancer chemopreventive flavonoid antioxidant, silymarin: inhibition of key attributes of vascular endothelial cells and angiogenic cytokine secretion by cancer epithelial cells.	Jiang C, Agarwal R, LÃ¼ J.	Biochem Biophys Res Commun. 2000	PMID:11006131
Endothelial-like cells from the bovine placental cotyledon.	Feng S, Peter AT, Asem EK.	In Vitro Cell Dev Biol Anim. 2000	PMID:11149752
Angiogenesis inhibition by transdominant mutant Ets-1.	Nakano T, Abe M, Tanaka K, Shineha R, Satomi S, Sato Y.	J Cell Physiol. 2000	PMID:10867651
Anti-angiogenic activity of torilin, a sesquiterpene compound isolated from Torilis japonica.	Kim MS, Lee YM, Moon EJ, Kim SE, Lee JJ, Kim KW.	Int J Cancer. 2000	PMID:10861486
Hepatocyte growth factor enhances MMP activity in human endothelial cells.	Wang H, Keiser JA.	Biochem Biophys Res Commun. 2000	PMID:10860849
Oversulfated fucoidan inhibits the basic fibroblast growth factor-induced tube formation by human umbilical vein endothelial cells: its possible mechanism of action.	Soeda S, Kozako T, Iwata K, Shimeno H.	Biochim Biophys Acta. 2000	PMID:10838166
Interleukin 6 promotes vasculogenesis of murine brain microvessel endothelial cells.	Fee D, Grzybicki D, Dobbs M, Ihyer S, Clotfelter J, Macvilay S, Hart MN, Sandor M, Fabry Z.	Cytokine. 2000	PMID:10843741
Anti-angiogenic cues from vascular basement membrane collagen.	Colorado PC, Torre A, Kamphaus G, Maeshima Y, Hopfer H, Takahashi K, Volk R, Zamborsky ED, Herman S, Sarkar PK, Ericksen MB, Dhanabal M, Simons M, Post M, Kufe DW, Weichselbaum RR, Sukhatme VP, Kalluri R.	Cancer Res. 2000	PMID:10811134
Eicosanoid regulation of angiogenesis: role of endothelial arachidonate 12-lipoxygenase.	Nie D, Tang K, Diglio C, Honn KV.	Blood. 2000	PMID:10733500
Rho-kinase inhibitor retards migration and in vivo dissemination of human prostate cancer cells.	Somlyo AV, Bradshaw D, Ramos S, Murphy C, Myers CE, Somlyo AP.	Biochem Biophys Res Commun. 2000	PMID:10720471
Inhibition of diclofenac formulated in hyaluronan on angiogenesis in vitro and its intraocular tolerance in the rabbit eye.	Shen WY, Constable IJ, Chelva E, Rakoczy PE.	Graefes Arch Clin Exp Ophthalmol. 2000	PMID:10796042
Inhibition of angiogenesis by blockers of volume-regulated anion channels.	Manolopoulos VG, Liekens S, Koolwijk P, Voets T, Peters E, Droogmans G, Lelkes PI, De Clercq E, Nilius B.	Gen Pharmacol. 2000	PMID:10974418
Defective epithelial-mesenchymal interactions dictate the organogenesis of tracheoesophageal fistula.	Crisera CA, Grau JB, Maldonado TS, Kadison AS, Longaker MT, Gittes GK.	Pediatr Surg Int. 2000	PMID:10898225
Sphingosine 1-phosphate induces angiogenesis: its angiogenic action and signaling mechanism in human umbilical vein endothelial cells.	Lee OH, Kim YM, Lee YM, Moon EJ, Lee DJ, Kim JH, Kim KW, Kwon YG.	Biochem Biophys Res Commun. 1999	PMID:10544002
Angiogenic activity of human soluble intercellular adhesion molecule-1.	Gho YS, Kleinman HK, Sosne G.	Cancer Res. 1999	PMID:10537287
Treatment with sulfasalazine or sulfapyridine, but not 5-aminosalicyclic acid, inhibits basic fibroblast growth factor-induced endothelial cell chemotaxis.	Volin MV, Harlow LA, Woods JM, Campbell PL, Amin MA, Tokuhira M, Koch AE.	Arthritis Rheum. 1999	PMID:10513809
Human IL-3 stimulates endothelial cell motility and promotes in vivo new vessel formation.	Dentelli P, Del Sorbo L, Rosso A, Molinar A, Garbarino G, Camussi G, Pegoraro L, Brizzi MF.	J Immunol. 1999	PMID:10438956

Title	Description	ShortDetails	Identifiers
Identification of endothelial cell binding sites on the laminin gamma 1 chain.	Ponce ML, Nomizu M, Delgado MC, Kuratomi Y, Hoffman MP, Powell S, Yamada Y, Kleinman HK, Malinda KM.	Circ Res. 1999	PMID:10189356
Phosphorothioate oligodeoxynucleotides inhibit basic fibroblast growth factor-induced angiogenesis in vitro and in vivo.	Kitajima I, Unoki K, Maruyama I.	Antisense Nucleic Acid Drug Dev. 1999	PMID:10355829
Endothelial cell apoptosis in capillary network remodeling.	Pollman MJ, Naumovski L, Gibbons GH.	J Cell Physiol. 1999	PMID:9989782
B1 integrin activation inhibits in vitro tube formation: effects on cell migration, vacuole coalescence and lumen formation.	Gamble J, Meyer G, Noack L, Furze J, Matthias L, Kovach N, Harlant J, Vadas M.	Endothelium. 1999	PMID:10599558
Identification of laminin alpha1 and beta1 chain peptides active for endothelial cell adhesion, tube formation, and aortic sprouting.	Malinda KM, Nomizu M, Chung M, Delgado M, Kuratomi Y, Yamada Y, Kleinman HK, Ponce ML.	FASEB J. 1999	PMID:9872929
In vitro endothelial differentiation of long-term cultured murine embryonic yolk sac cells induced by matrigel.	Li J, Wei Y, Wagner TE.	Stem Cells. 1999	PMID:10195567
Accutin, a new disintegrin, inhibits angiogenesis in vitro and in vivo by acting as integrin alphavbeta3 antagonist and inducing apoptosis.	Yeh CH, Peng HC, Huang TF.	Blood. 1998	PMID:9787163
Evidence that platelets promote tube formation by endothelial cells on matrigel.	Pipili-Synetos E, Papadimitriou E, Maragoudakis ME.	Br J Pharmacol. 1998	PMID:9863654 | PMCID:PMC1565699
Inhibition of tumor angiogenesis by roxithromycin, a 14-membered ring macrolide antibiotic.	Yatsunami J, Tsuruta N, Hara N, Hayashi S.	Cancer Lett. 1998	PMID:9851245
Early events in angiogenesis: cloning an alpha-prolyl 4-hydroxylase-like gene.	Cockerill GW, Varcoe L, Meyer GT, Vadas MA, Gamble JR.	Int J Oncol. 1998	PMID:9683799
Neuropeptide Y: a novel angiogenic factor from the sympathetic nerves and endothelium.	Zukowska-Grojec Z, Karwatowska-Prokopczuk E, Rose W, Rone J, Movafagh S, Ji H, Yeh Y, Chen WT, Kleinman HK, Grouzmann E, Grant DS.	Circ Res. 1998	PMID:9686758
The proteasome is involved in angiogenesis.	Oikawa T, Sasaki T, Nakamura M, Shimamura M, Tanahashi N, Omura S, Tanaka K.	Biochem Biophys Res Commun. 1998	PMID:9600100
Differential effects of hepatocyte growth factor isoforms on epithelial and endothelial tubulogenesis.	Montesano R, Soriano JV, Malinda KM, Ponce ML, Bafico A, Kleinman HK, Bottaro DP, Aaronson SA.	Cell Growth Differ. 1998	PMID:9607557
Inhibition of angiogenic differentiation of human umbilical vein endothelial cells by curcumin.	Thaloor D, Singh AK, Sidhu GS, Prasad PV, Kleinman HK, Maheshwari RK.	Cell Growth Differ. 1998	PMID:9563850
Clotrimazole, an imidazole antimycotic, is a potent inhibitor of angiogenesis.	Takahashi H, Abe M, Sugawara T, Tanaka K, Saito Y, Fujimura S, Shibuya M, Sato Y.	Jpn J Cancer Res. 1998	PMID:9617351
On the possible role of reactive oxygen species in angiogenesis.	Lelkes PI, Hahn KL, Sukovich DA, Karmiol S, Schmidt DH.	Adv Exp Med Biol. 1998	PMID:9889904
A new synthetic matrix metalloproteinase inhibitor modulates both angiogenesis and urokinase type plasminogen activator activity.	Shono T, Motoyama M, Tatsumi K, Ulbrich N, Iwamoto Y, Kuwano M, Ono M.	Angiogenesis. 1998	PMID:14517452
Constitutive expression of E- and P-selectin cognate ligands in human endothelial cells.	Palma C, Bellarosa D, Nardelli F, Mannori G, Manzini S.	Mediators Inflamm. 1998	PMID:9705609 | PMCID:PMC1781838
Inhibitory effect of lead on tube formation by cultured human vascular endothelial cells.	Ueda D, Kishimoto T, Dekio S, Tada M.	Hum Cell. 1997	PMID:9573489
Inhibitory effect of oversulfated fucoidan on tube formation by human vascular endothelial cells.	Soeda S, Shibata Y, Shimeno H.	Biol Pharm Bull. 1997	PMID:9401718
Tranilast inhibits the proliferation, chemotaxis and tube formation of human microvascular endothelial cells in vitro and angiogenesis in vivo.	Isaji M, Miyata H, Ajisawa Y, Takehana Y, Yoshimura N.	Br J Pharmacol. 1997	PMID:9401770 | PMCID:PMC1565049
Involvement of endothelial PECAM-1/CD31 in angiogenesis.	DeLisser HM, Christofidou-Solomidou M, Strieter RM, Burdick MD, Robinson CS, Wexler RS, Kerr JS, Garlanda C, Merwin JR, Madri JA, Albelda SM.	Am J Pathol. 1997	PMID:9284815 | PMCID:PMC1857836
Human omental microvascular endothelial and mesothelial cells: characterization of two distinct mesodermally derived epithelial cells.	Chung-Welch N, Patton WF, Shepro D, Cambria RP.	Microvasc Res. 1997	PMID:9327382
Thrombin promotes endothelial cell alignment in Matrigel in vitro and angiogenesis in vivo.	Haralabopoulos GC, Grant DS, Kleinman HK, Maragoudakis ME.	Am J Physiol. 1997	PMID:9252462
Thrombospondin-1, a natural inhibitor of angiogenesis, regulates platelet-endothelial cell adhesion molecule-1 expression and endothelial cell morphogenesis.	Sheibani N, Newman PJ, Frazier WA.	Mol Biol Cell. 1997	PMID:9243511 | PMCID:PMC276156

Advanced glycation end products-driven angiogenesis in vitro. Induction of the growth and tube formation of human microvascular endothelial cells through autocrine vascular endothelial growth factor.	Yamagishi Si, Yonekura H, Yamamoto Y, Katsuno K, Sato F, Mita I, Ooka H, Satozawa N, Kawakami T, Nomura M, Yamamoto H.	J Biol Chem. 1997	PMID:9079706
The role of cadherin-catenin-cytoskeleton complex in angiogenesis: antisense oligonucleotide of plakoglobin promotes angiogenesis in vitro, and protein kinase C (PKC) enhances angiogenesis through the plakoglobin signaling pathway.	Nagashima H, Okada M, Hidai C, Hosoda S, Kasanuki H, Kawana M.	Heart Vessels. 1997	PMID:9476558
Clarithromycin is a potent inhibitor of tumor-induced angiogenesis.	Yatsunami J, Turuta N, Wakamatsu K, Hara N, Hayashi S.	Res Exp Med (Berl). 1997	PMID:9440137
Ets-1 regulates angiogenesis by inducing the expression of urokinase-type plasminogen activator and matrix metalloproteinase-1 and the migration of vascular endothelial cells.	Iwasaka C, Tanaka K, Abe M, Sato Y.	J Cell Physiol. 1996	PMID:8952701
Cadmium injures tube formation by cultured human vascular endothelial cells.	Kishimoto T, Ueda D, Isobe M, Tada M.	Hum Cell. 1996	PMID:9183655
Sulfated malto-oligosaccharides bind to basic FGF, inhibit endothelial cell proliferation, and disrupt endothelial cell tube formation.	Foxall C, Wei Z, Schaefer ME, Casabonne M, Fugedi P, Peto C, Castellot JJ Jr, Brandley BK.	J Cell Physiol. 1996	PMID:8816920
Inhibition of endothelial cell differentiation on a glycosylated reconstituted basement membrane complex.	Kuzuya M, Satake S, Miura H, Hayashi T, Iguchi A.	Exp Cell Res. 1996	PMID:8806437
Microvascular endothelial cells from E-selectin-deficient mice form tubes in vitro.	Gerritsen ME, Shen CP, Atkinson WJ, Padgett RC, Gimbrone MA Jr, Milstone DS.	Lab Invest. 1996	PMID:8765318
A quantitative assay using basement membrane extracts to study tumor angiogenesis in vivo.	Ito Y, Iwamoto Y, Tanaka K, Okuyama K, Sugioka Y.	Int J Cancer. 1996	PMID:8690516
Establishment of an immortalized Copenhagen rat bone marrow endothelial cell line.	Yamazaki K, Lehr JE, Rhim JS, Pienta KJ.	In Vivo. 1996	PMID:8839795
AAMP, a conserved protein with immunoglobulin and WD40 domains, regulates endothelial tube formation in vitro.	Beckner ME, Liotta LA.	Lab Invest. 1996	PMID:8683944
Urokinase receptor antagonists inhibit angiogenesis and primary tumor growth in syngeneic mice.	Min HY, Doyle LV, Vitt CR, Zandonella CL, Stratton-Thomas JR, Shuman MA, Rosenberg S.	Cancer Res. 1996	PMID:8625323
Matrigel induces thymosin beta 4 gene in differentiating endothelial cells.	Grant DS, Kinsella JL, Kibbey MC, LaFlamme S, Burbelo PD, Goldstein AL, Kleinman HK.	J Cell Sci. 1995	PMID:8719875
Plasminogen activators augment endothelial cell organization in vitro by two distinct pathways.	Schnaper HW, Barnathan ES, Mazar A, Maheshwari S, Ellis S, Cortez SL, Baricos WH, Kleinman HK.	J Cell Physiol. 1995	PMID:7559792
Effects of extracellular matrices on tube formation of cultured rat hepatic sinusoidal endothelial cells.	Shakado S, Sakisaka S, Noguchi K, Yoshitake M, Harada M, Mimura Y, Sata M, Tanikawa K.	Hepatology. 1995	PMID:7657305
Establishment of immortalized Copenhagen rat prostate endothelial cell lines.	Yamazaki K, Lehr JE, Rhim JS, Pienta KJ.	In Vivo. 1995	PMID:8900918
Angiogenesis: role of calcium-mediated signal transduction.	Kohn EC, Alessandro R, Spoonster J, Wersto RP, Liotta LA.	Proc Natl Acad Sci U S A. 1995	PMID:7533291 | PMCID:PMC42508
Methylmercury-injury effect on tube formation by cultured human vascular endothelial cells.	Kishimoto T, Oguri T, Tada M.	Cell Biol Toxicol. 1995	PMID:7600257
Estrogen promotes angiogenic activity in human umbilical vein endothelial cells in vitro and in a murine model.	Morales DE, McGowan KA, Grant DS, Maheshwari S, Bhartiya D, Cid MC, Kleinman HK, Schnaper HW.	Circulation. 1995	PMID:7530174
Expression of tissue-type plasminogen activator and its inhibitor couples with development of capillary network by human microvascular endothelial cells on Matrigel.	Ito K, Ryuto M, Ushiro S, Ono M, Sugenoya A, Kuraoka A, Shibata Y, Kuwano M.	J Cell Physiol. 1995	PMID:7822431
Effect of lead on tube formation by cultured human vascular endothelial cells.	Kishimoto T, Oguri T, Ueda D, Tada M.	Arch Toxicol. 1995	PMID:8572931
Inhibitory effect of methylmercury on migration and tube formation by cultured human vascular endothelial cells.	Kishimoto T, Oguri T, Abe M, Kajitani H, Tada M.	Arch Toxicol. 1995	PMID:7495372
Production of angiogenesis inhibitors and stimulators is modulated by cultured growth plate chondrocytes during in vitro differentiation: dependence on extracellular matrix assembly.	Descalzi Cancedda F, Melchiori A, Benelli R, Gentili C, Masiello L, Campanile G, Cancedda R, Albini A.	Eur J Cell Biol. 1995	PMID:7538466
Angiogenesis as a component of epithelial-mesenchymal interactions.	Grant DS, Rose RW, Kinsella JK, Kibbey MC.	EXS. 1995	PMID:8527897
Group B streptococci (GBS) injure lung endothelium in vitro: GBS invasion and GBS-induced eicosanoid production is greater with microvascular than with pulmonary artery cells.	Gibson RL, Soderland C, Henderson WR Jr, Chi EY, Rubens CE.	Infect Immun. 1995	PMID:7806366 | PMCID:PMC172988
Inhibitors of basement membrane collagen synthesis prevent endothelial cell alignment in matrigel in vitro and angiogenesis in vivo.	Haralabopoulos GC, Grant DS, Kleinman HK, Lelkes PI, Papaioannou SP, Maragoudakis ME.	Lab Invest. 1994	PMID:7526036
Characterization of a spontaneously transformed human endothelial cell line.	Cockerill GW, Meyer G, Noack L, Vadas MA, Gamble JR.	Lab Invest. 1994	PMID:7526034

Inhibition of angiogenesis by anthracyclines and titanocene dichloride.	Maragoudakis ME, Peristeris P, Missirlis E, Aletras A, Andriopoulou P, Haralabopoulos G.	Ann N Y Acad Sci. 1994	PMID:7526759
Lymphangiogenesis in vitro: formation of lymphatic capillary-like channels from confluent monolayers of lymphatic endothelial cells.	Leak LV, Jones M.	In Vitro Cell Dev Biol Anim. 1994	PMID:7987539
Opposing effects on modulation of angiogenesis by protein kinase C and cAMP-mediated pathways.	Tsopanoglou NE, Haralabopoulos GC, Maragoudakis ME.	J Vasc Res. 1994	PMID:7517701
Evidence that nitric oxide is an endogenous antiangiogenic mediator.	Pipili-Synetos E, Sakkoula E, Haralabopoulos G, Andriopoulou P, Peristeris P, Maragoudakis ME.	Br J Pharmacol. 1994	PMID:7517330 | PMCID:PMC1910111
Primary culture of microvascular endothelial cells from canine meniscus.	Miller RR, Rydell PA.	J Orthop Res. 1993	PMID:7506768
Type IV collagenase(s) and TIMPs modulate endothelial cell morphogenesis in vitro.	Schnaper HW, Grant DS, Stetler-Stevenson WG, Fridman R, D'Orazi G, Murphy AN, Bird RE, Hoythya M, Fuerst TR, French DL, et al.	J Cell Physiol. 1993	PMID:8344982
Role of protein kinase C in tumor necrosis factor induction of endothelial cell urokinase-type plasminogen activator.	Niedbala MJ, Stein-Picarella M.	Blood. 1993	PMID:7683925
Modulation of morphological differentiation of human endothelial cells in culture by the synthetic peptide YIGSR and cytochalasin B.	Kubota Y, Mizoguchi M.	Clin Exp Dermatol. 1993	PMID:8348717
Cytokine regulation of endothelial cell extracellular proteolysis.	Niedbala MJ.	Agents Actions Suppl. 1993	PMID:8356923
Cytokine activation of human macro- and microvessel-derived endothelial cells.	Gerritsen ME, Niedbala MJ, Szczepanski A, Carley WW.	Blood Cells. 1993	PMID:7906155
Isolation, cultivation, and partial characterization of microvascular endothelium derived from human lung.	Carley WW, Niedbala MJ, Gerritsen ME.	Am J Respir Cell Mol Biol. 1992	PMID:1333246
Interaction of endothelial cells with a laminin A chain peptide (SIKVAV) in vitro and induction of angiogenic behavior in vivo.	Grant DS, Kinsella JL, Fridman R, Auerbach R, Piasecki BA, Yamada Y, Zain M, Kleinman HK.	J Cell Physiol. 1992	PMID:1280280
In vitro model of angiogenesis using a human endothelium-derived permanent cell line: contributions of induced gene expression, G-proteins, and integrins.	Bauer J, Margolis M, Schreiner C, Edgell CJ, Azizkhan J, Lazarowski E, Juliano RL.	J Cell Physiol. 1992	PMID:1280276
Inhibition of angiogenesis in vitro and in ovo with an inhibitor of cellular protein kinases, MDL 27032.	Wright PS, Cross-Doersen D, Miller JA, Jones WD, Bitonti AJ.	J Cell Physiol. 1992	PMID:1380511
Tumor necrosis factor regulation of endothelial cell extracellular proteolysis: the role of urokinase plasminogen activator.	Niedbala MJ, Stein-Picarella M.	Biol Chem Hoppe Seyler. 1992	PMID:1381189
Protein kinase C regulates endothelial cell tube formation on basement membrane matrix, Matrigel.	Kinsella JL, Grant DS, Weeks BS, Kleinman HK.	Exp Cell Res. 1992	PMID:1370939
Tumor necrosis factor induction of endothelial cell urokinase-type plasminogen activator mediated proteolysis of extracellular matrix and its antagonism by gamma-interferon.	Niedbala MJ, Picarella MS.	Blood. 1992	PMID:1732009
Growth and differentiation of the cultured secretory cells of the cow oviduct on reconstituted basement membrane.	Joshi MS.	J Exp Zool. 1991	PMID:1940825
Intracellular mechanisms involved in basement membrane induced blood vessel differentiation in vitro.	Grant DS, Lelkes PI, Fukuda K, Kleinman HK.	In Vitro Cell Dev Biol. 1991	PMID:1856158
Differential effects of interferon gamma and alpha on in vitro model of angiogenesis.	Maheshwari RK, Srikantan V, Bhartiya D, Kleinman HK, Grant DS.	J Cell Physiol. 1991	PMID:1703547
A hemangioendothelioma-derived cell line: its use as a model for the study of endothelial cell biology.	Obeso J, Weber J, Auerbach R.	Lab Invest. 1990	PMID:2166185
Two different laminin domains mediate the differentiation of human endothelial cells into capillary-like structures in vitro.	Grant DS, Tashiro K, Segui-Real B, Yamada Y, Martin GR, Kleinman HK.	Cell. 1989	PMID:2528412
